# Beta-Band Stimulation of Dorsolateral Prefrontal Cortex Through tACS Alters Mental Time Travel and Prospective Memory, but Not Delay Discounting

**DOI:** 10.3390/brainsci16090899

**Published:** 2026-08-22

**Authors:** Alex Marson, Giorgia Cona

**Affiliations:** 1 Padova Neuroscience Center, University of Padova, Via Orus 2/B, 35129 Padova, Italy; alex.marson@phd.unipd.it; 2 Department of General Psychology, University of Padova, Via Venezia, 35131 Padova, Italy

**Keywords:** transcranial alternating current stimulation, dorsolateral prefrontal cortex, future cognition, mental time travel, delay discounting, prospective memory

## Abstract

**Highlights:**

**What are the main findings?**

- Beta-tACS over bilateral DLPFC selectively improves mental time travel accuracy for past and present reference points, leaving future projections unaffected.
- Beta-tACS increases delay discounting steepness and enhances time-based prospective memory precision, without altering clock-checking behavior.

**What are the implications of the main findings?**

- DLPFC beta oscillations support the maintenance of concrete, temporally proximal representations rather than a unitary future-cognition mechanism.

**Abstract:**

**Background**: Future-oriented cognition involves several neurocognitive processes putatively supported by the dorsolateral prefrontal cortex (DLPFC) and beta-band oscillations. This study aimed to corroborate the role of beta oscillations in the DLPFC during three future cognition tasks: mental time travel (MTT), delay discounting (DD), and time-based prospective memory (PM). **Methods**: Thirty-three participants (22–29 years) completed the three tasks under two conditions—active and sham stimulation—administered in a counterbalanced within-subjects design, one week apart. Beta-frequency transcranial alternating current stimulation (tACS; 22 Hz, 1.5 mA) was applied bilaterally over the DLPFC (F3–F4). **Results**: Beta-tACS was associated with dissociable effects across domains. In MTT, stimulation selectively improved accuracy for ordinality judgements anchored to past and present reference points (2014, 2024), without affecting future projections (2034) or reaction times. In DD, beta-tACS did not modify the steepness of temporal discounting in a statistically significant way. In PM, stimulation enhanced both dichotomous accuracy and continuous temporal precision, with no changes in clock-checking behavior. **Conclusions**: These findings provide preliminary evidence that suggests a possible role of DLPFC beta oscillations in supporting the maintenance and utilization of more concrete temporal representations across distinct temporal domains, resulting in anchoring in more available representations (MTT) and focus on temporal information (PM), challenging unitary accounts of prefrontal beta function and highlighting task-dependent neuromodulation effects within the future temporal domain.

## 1. Introduction

Future-related cognition encompasses a series of real-life processes, ranging from organizing one’s own behavior around the schedule of the day to complex long-term financial planning. The term “prospection” has been broadly used to denote these cognitive operations, all of which require a mental representation of future time. This umbrella concept comprises cognitive functions like future simulation, prediction, intention and planning, with varying degrees of reliance on semantic or episodic memory systems [1].

Given the shared domain of future-time representation and the ubiquity of prospection in our daily lives, researchers investigated whether these different processes might rely on a common neural architecture. While individual studies have mapped distinct neural substrates of specific future-related tasks, a recent meta-analysis examining shared activation patterns identified a neural posterior-to-anterior activation gradient over the frontal and prefrontal cortex (PFC), mirroring the concrete-to-abstract nature of the processing of future elements [2]. Furthermore, additional meta-analytic evidence suggests a link between future-related cognition and non-temporal aspects, such as navigation or theory of mind, finding a significant overlap with such domain-general areas [3].

Despite substantial progress in mapping the functional neuroanatomy of future processing, most existing research relies on correlational neuroimaging approaches, providing limited insight into causal relationships between neural activity and future-oriented cognition. The present study aims to address this limitation by investigating the role of beta oscillations in the dorsolateral prefrontal cortex (DLPFC) using transcranial alternating current stimulation (tACS) across three distinct temporal cognition tasks: mental time travel (MTT), delay discounting (DD), and time-based prospective memory (PM). The task choice relies on the fact that the taxonomy of prospection proposed by Szpunar and colleagues [1] identifies Future Thinking (including MTT), delay discounting, and prospective memory as core future-oriented functions. All these functions, in fact, require the processing and updating of future-oriented information. The following paragraphs provide an in-depth examination of these functions, clarifying why and how they involve the construction and processing of future-oriented representations. In addition, their neural and electrophysiological (EEG) underpinnings will be discussed, with particular emphasis on the role of our candidate investigation region, the dorsolateral prefrontal cortex (DLPFC), and our candidate oscillatory activity, beta oscillations, in each domain. In fact, we hypothesize that beta oscillatory activity over the DLPFC plays a domain-general role in future-related cognition and, as such, contributes to all the future-oriented functions.

To test this hypothesis, we employed transcranial alternating current stimulation (tACS), a non-invasive brain stimulation technique that delivers a sinusoidal electrical current at a predefined frequency and induces rhythmic fluctuations in cortical excitability. This technique is meant to enhance frequency-specific neural activity by aligning endogenous neural oscillations with the externally applied stimulation frequency in a process called entrainment [4,5]. Thus, it allows researchers to provide tentative evidence of causal relationships between frequency-specific neural oscillations in targeted brain regions and cognitive functions by selectively modulating power within a frequency band previously associated with the function of interest in the EEG literature [6].

### 1.1. Prospective Memory

Prospective memory (PM) is the ability to remember to execute a specific, pre-defined action in the future [7]. Such ability is often deployed in everyday life, when remembering to pick up one’s own child at school at a specific hour, turning the dish in the oven after 20 min, calling a friend for their birthday, or warning a neighbor on request when their package arrives in the flat. PM tasks differ fundamentally in their triggering mechanisms and can be categorized as either event-based or time-based [8]. Event-based PM requires individuals to perform an intended action upon encountering a specific environmental cue, whereas time-based PM demands self-initiated action execution at a predetermined time, regardless of external prompts—even though the support of an external clock can be admitted [8]. For instance, notifying a neighbor about a package delivery exemplifies event-based PM, as the action is triggered by the external cue of the package’s arrival at an unspecified time. Conversely, rotating a dish in the oven during cooking represents time-based PM, requiring internal time monitoring and self-initiated action after a specific interval has elapsed.

As such, a time-based prospective memory (time-based PM) task inherently entails constructing a representation of a future time. Cognitively, the most common model used to explain time-based PM processes is the Test-Wait-Test-Exit (TWTE) framework, which involves repeated cycles of time monitoring and waiting [9]. In this model, individuals periodically check whether the target time has been reached (test phase) and either continue recursively with ongoing activities while maintaining the intention in memory to check the time once again (wait phase) or, upon reaching the designated time point, execute the intended action (exit phase). For example, when remembering to turn a dish after 10 min of cooking, one would periodically check the time, continue with other activities, and finally perform the turning action when the specified duration has elapsed. Every testing action is a self-initiated process; therefore, top-down processes are implied both for the maintenance of the future-oriented intention and for the monitoring of the system state (i.e., elapsed time).

A meta-analytic study highlighted the crucial role of the dorsolateral prefrontal cortex (DLPFC) in both time-based and event-based prospective memory tasks, along with the pre-supplementary motor area (pre-SMA), frontal eye fields (FEF), superior parietal lobule (SPL), and precuneus [10]. Activity within this network was associated with increased top-down control processes supporting intention maintenance and strategic monitoring (i.e., checking elapsed time in time-based PM and monitoring the environment for target cues in event-based PM [10]). These findings informed the “Attention to Delayed Intentions” (AtoDI) model, which posits that a dorsal frontoparietal network allocates attentional resources to intention maintenance rather than to intention-irrelevant environmental processing [10].

Concerning non-invasive brain stimulation approaches targeting the DLPFC, contrasting results were found. For example, Bisiacchi and colleagues [11] applied repetitive transcranial magnetic stimulation (rTMS) to either the left or the right DLPFC and observed a selective impairment in event-based PM performance following right DLPFC stimulation during an early time window, but not during a later one. Based on this pattern of findings, the authors suggested that the right DLPFC is causally involved in strategic monitoring processes (namely the attentional and memory processes required to maintain the intention active in mind and monitor the environment for the presence of the PM cue). Other studies applied anodal transcranial direct current stimulation (tDCS) on the left DLPFC instead, expecting an improvement in strategic monitoring of PM cues when compared with a sham condition, but failed to find any significant stimulation effects [12,13].

Regarding frequency-specific electrophysiological correlates of prospective memory (PM), only a few studies have reported putative associations with different oscillatory bands. In an EEG study, a weaker theta power and suppression of alpha power in the anterior cingulate cortex (ACC) were found after a time check in a time-based PM task, an effect probably associated with attentional/control rather than time-estimation processes [14]. On the other hand, in event-based PM tasks, alpha suppression and theta increase in lateral frontal regions were shown to be associated with orientation to external cues and internal cues, with the former expressed more on lateral and the latter expressed more on medial areas of the prefrontal cortex, as pointed out by a recent MEG study [15]. Other authors also found a role of alpha/theta [16] and alpha/beta [17] oscillations in parietal regions. However, it seems frontal beta oscillations are also implied in PM, and specifically in time-based PM: a recent study by Santacesaria and colleagues [18] employed high-density EEG (hd-EEG) during an ecologically valid prospective memory task (i.e., watching a movie) that required either event-based or time-based responses. The authors reported a selective increase in high-beta spectral power density during time-based PM, potentially linking beta oscillations to the time-estimation demands of the task.

### 1.2. Mental Time Travel

Mental time travel (MTT) was originally defined as the ability to mentally relive past events and simulate and imagine future ones [19]. As such, it implies the capability of experiencing oneself as a subjectivity placed in an organized timeline of events [20]. Some authors have adopted a more consciousness-centered approach to the self-in-time, focusing on the phenomenological dimensions of what Tulving [21] termed autonoetic consciousness. Other authors have emphasized the role of episodic memory and the constructive/simulative nature of both remembering the past and imagining the future. When oriented toward the future, this conception of mental time travel (MTT) as “pre-living” events—analogous to how we “re-live” personal memories—is referred to as Episodic Future Thinking (EFT) [22]. Notwithstanding the different approaches to mental time travel (MTT), certain regions—most notably the dorsolateral prefrontal cortex (DLPFC)—appear to be consistently implicated across several underlying mechanisms, including self-projection, episodic memory, scene construction, and navigation. This convergence has led some authors to propose a general role for the DLPFC in self-projection and the maintenance of cognitive maps across multiple domains [23,24]. According to the framework of cognitive maps [25], the ability to mentally time travel will be probed in relation to the capability of the subjects to place and orient themselves in a metric representation of past and future events [26]. Namely, the temporal structure organizing future and past events that the subject mentally travels through is cognitively (and neurally) represented. In such a framework, a paradigm is employed in which participants are asked to project themselves into a certain point in the timeline and make judgments from there about target events that could happen before or after the projection reference point [26]. During such tasks, DLPFC activation has been associated with domain-specific mechanisms. In particular, activity in this region appears to index the absolute temporal distance of self-projection from the present moment [27]. In contrast, other studies have shown that left DLPFC activation tracks the relative distance of events within a cognitive map, computed from the point of self-projection in time [28]. Together, these findings suggest that the DLPFC may support a relative coordinate-indexing system that enables shifts from reasoning anchored in the “present self” to reasoning from a “past self” or “future self” perspective. So far, on the other hand, the literature exploring the role of brain oscillatory activity in mental time travel is limited. The role of neural oscillations in representing large-scale temporal distances—such as the distance of a target event within a mental time map spanning several years—has only recently been highlighted by a neurostimulation study conducted by D’Angelo and colleagues [29]. In this study, participants were presented with faces of different ages while receiving parietal transcranial alternating current stimulation (tACS) at beta or alpha frequencies, or sham stimulation. They were asked to evaluate the likelihood that the depicted individual had experienced specific target events within the past 10 years or would experience them within the subsequent 10 years. Under beta stimulation—but not alpha or sham—participants showed a significant shift toward younger faces when judging the likelihood that the depicted person had experienced events in the previous decade, despite the absence of any bias in age estimation. To date, this represents the first and only evidence linking beta oscillations to the perception of large-scale temporal distances, specifically demonstrating an association between increased parietal beta power and a reduction in the perceived temporal distance of past events. These findings extend previous research on Episodic Future Thinking (EFT), conducted independently of a classical mental time travel (MTT) paradigm, which reported enhanced beta oscillatory activity in frontopolar regions during future-episodic compared to past-episodic and non-episodic thinking [30]. Together, these results suggest a potential role for beta oscillations in large-scale future-oriented cognition.

### 1.3. Intertemporal Choice

Intertemporal choice refers to a decision-making process in which a person must weigh trade-offs between the immediate and delayed consequences of a given option [31]. For example, one might choose between spending a certain amount of money on a weekend getaway today or saving it for a longer holiday next year. Within the context of intertemporal choice, a robust phenomenon has consistently been observed: the tendency to devalue positive outcomes as a function of delay. This phenomenon is known as delay discounting [32]. Delay discounting has been widely studied, as it has attracted interest due to its cross-species occurrence, the simplicity and reliability of its measurement, and its associations with both psychological and life-relevant variables [33].

Literature has highlighted the contribution of three neural networks: the reward processing network—the ventromedial prefrontal cortex, ventral striatum, posterior cingulate cortex, which encodes the subjective value of a certain choice outcome; the prospection network—medial temporal lobe, praecuneus, dorsomedial prefrontal cortex—responsible for the construction of future scenarios; and the executive control network—dorsolateral prefrontal cortex and dorsal anterior cingulate cortex—responsible for complex reasoning like trade-offs [33]. The dorsolateral prefrontal cortex (DLPFC) therefore plays a critical role in intertemporal choice, and its involvement has been recently confirmed through a meta-analysis [2]. However, the precise functional contribution of the DLPFC remains still debated. Some researchers suggest that DLPFC activity reflects the cognitive demandingness of the decision-making process, as evidenced by its activation during both gain and loss discounting (i.e., when the trade-off pertains to negative consequences and therefore recruits the additional phenomenon of gain-loss asymmetry), with stronger activation observed in the latter condition [34]. Alternatively, DLPFC activation may index the degree of self-control required during choice, particularly as its left hemisphere shows increased activation when participants select larger-but-later rewards [35,36,37,38,39,40]. Causal evidence from neuromodulation studies further supports the self-control account but also introduces some considerations on lateralization: disrupting left, but not right, DLPFC activity with repetitive transcranial magnetic stimulation (rTMS) increases the selection of smaller-sooner rewards [41], even though the reverse effect is found when the same area is inhibited through cathodal tDCS stimulation. Additionally, individual differences in structural connectivity to DLPFC correlate significantly with individual differences in discount rates [42].

As for the oscillatory component of the underlying neural mechanisms, electrocorticography recordings from lateral PFC reveal that theta oscillations are significantly elevated during impulsive decisions, whereas beta oscillations predominate during non-impulsive choices, suggesting functional dissociation between frequency bands in decision outcomes [43]. Indeed, tACS-induced desynchronization of theta activity between frontal and parietal areas seems to induce more discounting [44]. Overall, oscillatory evidence suggests a role of frontal frequency dynamics in regulating intertemporal choice, though the specific contribution of beta activity remains underexplored. The beta profile, specifically, has not been investigated through neurostimulation yet, leaving a gap in the literature.

### 1.4. Time Perspective

A secondary aim of the present study concerns the exploratory investigation of personality correlates of task performance, irrespective of tACS condition. Although several personality dimensions are inherently temporal in nature—such as procrastination or pacing style—the systematic integration of personality measures with cognitive paradigms targeting the psychological experience of time remains relatively uncommon. The most widely used instrument in this domain is the Zimbardo Time Perspective Inventory (ZTPI) [45], which captures individual differences in the tendency to orient cognitively and emotionally toward the personal past, present, or future. Time perspective (TP) has been linked to a broad range of psychological and behavioral outcomes [46], yet its relationship with the specific cognitive tasks employed in the present study has received limited empirical attention. The most investigated, albeit still debated, association concerns delay discounting: higher scores on the Present Hedonistic scale (reflecting a tendency to prioritize immediate gratification over future consequences) and lower scores on the Future scale (reflecting goal-directed self-regulation) have been associated with steeper discounting rates [47,48,49]. By contrast, the relationship between TP and time-based prospective memory, or mental time travel, has received little to no systematic investigation [50,51].

Such sparse yet promising literature hints at a possible connection between temporal personality traits and the cognitive components of temporal cognition. To expand and clarify such a connection, the present study employs the Control-Affect-Time (CAT) Perspective Questionnaire [52] rather than the ZTPI, as it offers a more fine-grained and theoretically updated operationalization of time perspective.

### 1.5. The Present Study

Based on the literature described in previous paragraphs, we hypothesized that the DLPFC might play a role in each future-oriented function, making the DLPFC a potential good candidate for a transversally implied region for future-oriented cognition. Also, the candidate frequency band to be tested is beta oscillations, due to their reported DLPFC-located implication in time-based PM (likely connected to time-estimation processes) [18] and Intertemporal Choice (likely connected to executive processes) [43]. For MTT, finally, beta oscillations have proved relevant, even though they have been experimentally tested only in parietal areas so far [29]. More generally, beyond task-specific correlational evidence, a broader theoretical support comes from the “beta-as-status-quo” hypothesis [53], which proposes that beta oscillations serve to maintain currently active neural representations against disruptive inputs, paralleling the top-down maintenance role of DLPFC. Based on these accounts, it can be hypothesized that beta oscillations characterize all the three functions, each of which requires the sustained maintenance of a future-oriented representation against competing demands.

To test these hypotheses, participants will undergo the three tasks while receiving either a beta-tACS or a sham stimulation over bilateral DLPFC in a within-subjects design. This would lead to an experimental, selective increase in beta activity in the DLPFC and, in turn, to subsequent changes in participants’ behavior during such tasks. According to the aforementioned pieces of research, we expect that as compared with sham stimulation, beta-tACS would be associated with: (a) an increase in time-based PM, as a consequence of an increase in temporal-processing or even executive capabilities; (b) a possible increase in MTT performance, especially for future (and past) time frames of reference, as a consequence of an increase in the self-projection efficiency and/or map maintenance capability; (c) a greater preference for bigger-but-later rewards compared to smaller-but-immediate ones, as a result in increased executive control. According to previous correlational evidence, the hypothesis pertaining to time-based PM is the most robust, while the one concerning MTT is the most putative.

A more exploratory part of the study is dedicated to the investigation of the correlations between personality aspects related to time and temporal cognitive indicators.

## 2. Materials and Methods

### 2.1. Participants

A convenience sample of university students (*N* = 36) was accordingly recruited through word-of-mouth referrals. The sample size was determined based on comparable tACS studies in the literature [29]. No compensation was provided for participation. Inclusion criteria [54] required participants to have no previous experience with tES, no history of neurological or cardiac disorders, no current psychiatric conditions or scalp skin diseases, no current medication regime affecting cortical excitability, no metallic implants, pacemakers, drug infusion pumps, hearing aids, or neurostimulation devices. Pregnant individuals were excluded from participation. Participants were instructed to abstain from consuming more than three alcohol units, more than two cups of coffee, or any psychoactive substance the 24 h prior to each stimulation session.

Upon recruitment, participants received comprehensive information regarding the experimental protocol, procedural details, and safety considerations associated with transcranial alternating current stimulation (tACS). A list of contraindications was provided, and the absence of exclusion criteria was verified through structured interviews conducted by the experimenter prior to each session. Due to technical difficulties during response registration, valid data was collected for both tasks and time points for only *N* = 33 participants (~54% males, *M*_age_ = 23.58 years, *SD* = 1.15).

The study complies with the PECANS guidelines [55], although the authors did not pre-register hypotheses and analysis plans on a journal or public repository.

### 2.2. Stimulation Apparatus and Parameters

Transcranial electrical stimulation was delivered using a BrainSTIM Transcranial Stimulator (E.M.S. s.r.l., Bologna, Italy), a programmable bidirectional current transcranial stimulator (CE 0476). Two electrodes were housed in 35 cm^2^ sponges (7 × 5 cm) saturated with 0.9% saline solution. Stimulation consisted of a 22 Hz sinusoidal current delivered at an intensity of 1500 μA, with the waveform centered on zero current (i.e., without a DC offset) and with 15 s ramp-up and ramp-down phases. The device was programmed to prevent stimulation delivery when electrode impedance exceeded 5 kΩ, but it did not record impedances. In the sham condition, stimulation was delivered for 30 s using the same 22 Hz waveform and ramping parameters, while the active condition consisted of a continuous stimulation at 22.00 Hz with identical ramp parameters and intensity specifications. Stimulation was interrupted upon completion of all the tasks. Electrode placement was determined using anatomical landmarks corresponding to the F3 and F4 positions of the 10–20 international system, with distances measured using a tape measure. Target locations were marked with a skin-friendly marker, and the electrodes were placed with markers centered beneath each of them. When impedance levels exceeded acceptable thresholds, additional saline solution was applied on both sites using a syringe. Following successful impedance verification, participants underwent a 5 min habituation period, with the possibility to interrupt the stimulation if experiencing distress. On average, active stimulation sessions lasted 13.17 m (SD = 2.18 m, range = [9.56–22.26 m].

### 2.3. Experimental Procedure

At least 48 h prior to the initial session, participants received a list of events to memorize, with each event briefly described and associated with its corresponding date (year) and the specific wording to be used during the mental time travel (MTT) task. During the first session, the experimental paradigm and tACS procedure were explained again, informed consent was obtained, and a structured safety assessment was conducted to ensure safe participation in tACS stimulation.

**Figure 1:**
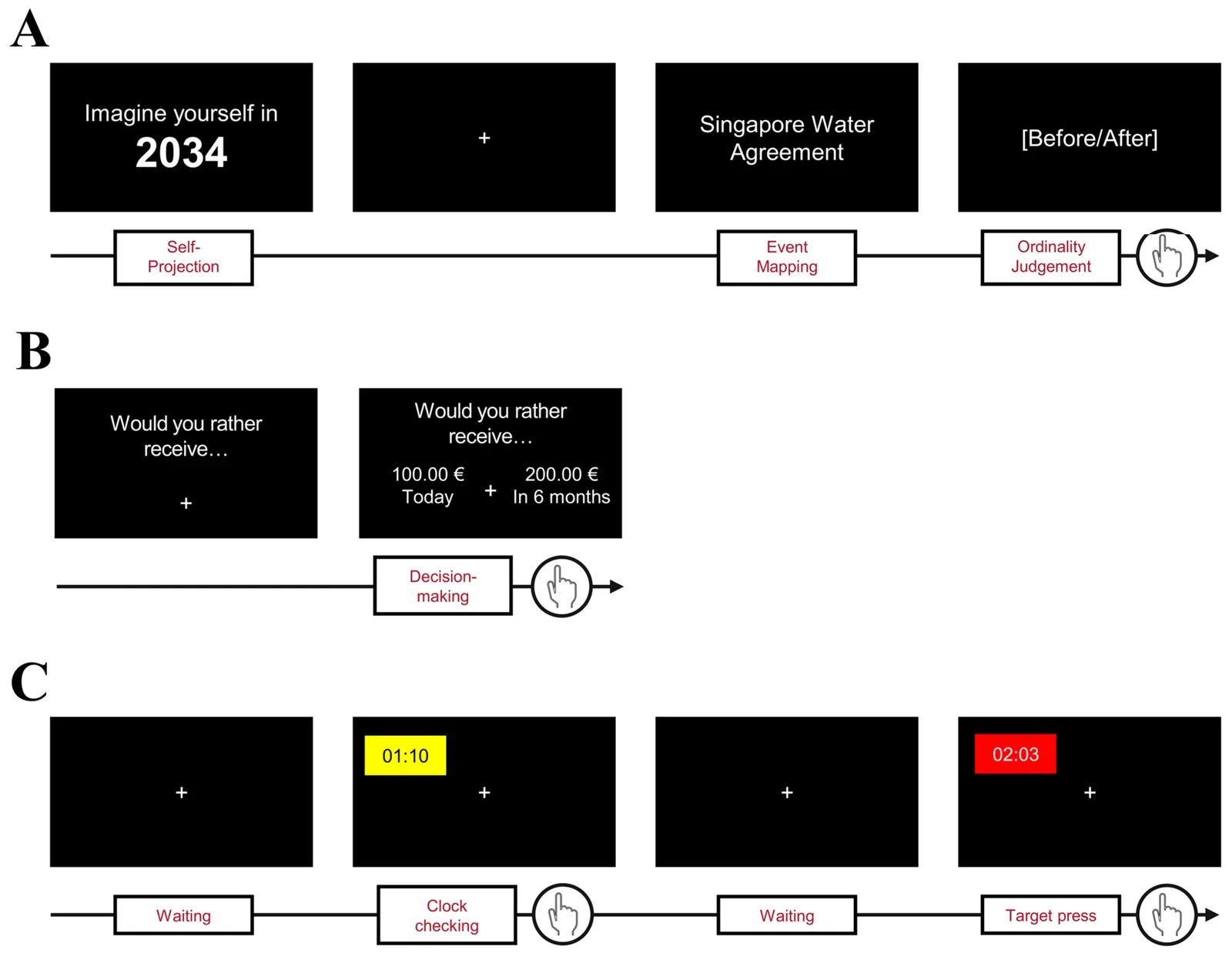
Structure of the experimental tasks. Panel (**A**): mental time travel (MTT) task—participants imagine themselves in a target reference year and, from there, judge whether a certain event happened/would happen before or after. Panel (**B**): delay discounting task (DD)—participants are offered a hypothetical choice between a smaller-but-immediate and a larger-but-delayed reward. Panel (**C**): time-based prospective memory task (time-based PM)—participants, while performing DD, are asked to press a button every 2 min. They can check the elapsed time from the last press through a temporary clock (yellow). The hand symbol indicates the possibility for the participant to press a button/and or provide a response.

The order of stimulation sessions (active vs. sham) was counterbalanced across participants and randomly assigned, who remained blinded to the condition (single-blind design). Three tasks were administered: the mental time travel (MTT) task and the delay discounting (DD) task coupled with the time-based prospective memory (PM) task. Task order was counterbalanced across participants and maintained consistently across stimulation sessions. Following task completion, participants completed questionnaires (see Section 2.4). The second session occurred one week later, after which participants also completed the Control-Affect-Time (CAT) Perspective Questionnaire. The tasks are illustrated in Figure 1.

#### 2.3.1. Mental Time Travel Task

Participants were presented with reference years (2014, 2024, or 2034) and instructed to mentally project themselves into those time points. The mental time travel task required participants to indicate whether a given presented event (e.g., “Los Angeles Olympic Games”) occurred before or after the reference year. The order and sequence of reference years were counterbalanced across participants and maintained consistently across stimulation sessions. Each reference presentation displayed a screen with the instruction “*Imagine you are in the [year]*:” followed by the target reference year, for a duration of 10 s, followed by a 1 s fixation point, and then the event to be judged, which remained visible for 5 s or until response (see Figure 1). All 24 events for each reference were presented in randomized order, separated by 1 s fixation points, with no repetition of the reference presentation. Following completion of all 24 events for a given reference, the subsequent reference year was presented.

Responses were recorded using the “6” and “b” keyboard keys, positioned vertically to eliminate spatial associations with temporal judgments. Key-response mappings (before/after) were randomized across participants and stimulation sessions. Upon task completion, participants were offered a brief rest period if the MTT was administered first.

Prior to the experimental trials, participants completed four practice events (“Los Angeles Olympic Games”, “Biggest supermoon”, “You learn to ride a bike”, “Koala gets extinct”), using a randomized reference point. The limited number of practice trials was implemented to minimize electrical stimulation exposure. Reaction times (RTs) and accuracy were recorded for each response. Accuracy was determined based on objective dates for semantic events, self-reported occurrence years for past episodic events, and culturally relevant predetermined standards for future episodic events (see Supplementary Materials).

#### 2.3.2. Delay Discounting Task

In each trial, participants saw the question “Would you rather receive…” at the top of the screen, with two monetary options displayed in the center (e.g., €200 now vs. €400 in 1 month). The choice screen remained visible for a maximum of 5 s or until a response. If participants failed to respond within the time limit, the immediate amount was represented in the subsequent trial. Following each response, the smaller-but-immediate reward value was adjusted according to a titration design with an established binary search algorithm [56]. In this procedure, the immediate reward amount in the first choice was set to 50% of the delayed reward amount; after each choice, the subsequent immediate reward amount was defined as the midpoint between the last accepted and the last rejected immediate reward values. When no immediate option had yet been accepted, the maximum value was used for the computation; conversely, when no immediate option had yet been rejected, zero was used. Through this iterative process, the immediate reward value converged toward the so-called indifference point, which theoretically represents the subjective value of the delayed reward—namely, the immediate amount at which the participant is equally likely to choose the immediate or the delayed option.

After six trials, the initial amounts were then represented, and the procedure was repeated with different delays for the delayed reward across all possible intervals (1 month, 6 months, 1 year, 3 years, 5 years, 10 years). Subsequently, the procedure was repeated with varying maximum delayed amounts (€400, €10,000, €40,000). The sequence of delays and maximum amounts remained constant across participants and conditions. Responses were recorded using left and right arrow keys, pressed with the right hand, corresponding respectively to left and right screen options to facilitate rapid dual-task performance. Upon completion, participants were offered a brief rest period if the DD Task was administered first. Prior to experimental trials, participants completed four practice trials with delay-amount combinations not used in the main task.

#### 2.3.3. Prospective Memory Task

The prospective memory (PM) task was administered concurrently with the delay discounting task. For its intrinsic nature, the PM task must be executed simultaneously with an ongoing activity. We decided to use the DD task as the ongoing task. This was possible because the DD task is not a performance-based task and allowed us to shorten the total duration of the tasks, thus maximizing the effects of the stimulation (as the effects of tACS tend to diminish over time). Participants were instructed to remember to press the “X” key every 2 min. Upon pressing “X”, elapsed time was displayed for 1 s in red, signaling cycle completion. Participants could monitor the elapsed time since the previous 2 min cycle by pressing the “W” key. When “W” was pressed, the current elapsed time appeared for 1 s in yellow. Response timestamps for both key presses were recorded.

### 2.4. Materials and Stimuli

MTT stimuli. The MTT task employed 53 events: 29 semantic events (unrelated to participants’ personal lives and pre-learned with associated dates) and 24 episodic events (likely to occur within participants’ lifespans). For each reference year, 12 semantic and 12 episodic events were presented, with some events repeated across time references (maximum of 2 repetitions for semantic events, maximum of 3 for episodic events). Semantic events were equally distributed before and after each reference year and evenly spaced temporally. Due to individual life course variability and participant age constraints, equal temporal distribution was not feasible for episodic events. Complete event lists, dates, temporal distribution, and mean valence and arousal ratings are provided in the Supplementary Materials. A complete list of items used in the MTT task can be found in File S1, in the Supplementary Materials.

Self-Assessment Mannequin (SAM). Valence and arousal for each MTT event were collected using the Self-Assessment Mannequin (SAM) [57]. For each event, participants can respond on a 5-point Likert scale in which each point is a pictorial representation of the level of arousal or valence. Additionally, participants reported whether they experienced a certain personal event and if so, at which age.

Control-Affect-Time Model of Time Perspective Questionnaire (CAT) [52]. A 39-item scale measuring time perspective across eight subscales: Future Anxiety, Future Proactive, Present Fatalistic, Present Hedonistic, and Present Resilient. Present Eudaimonic, Past Negative, Past Nostalgic. Responses are provided on a 5-point Likert scale (1 = “*Strongly Disagree*”; 5 = “*Strongly agree*”).

Physical Feelings Questionnaire. This questionnaire comprises a 7-item scale measuring the intensity of eventual feelings of itching, pain, burning, heat, tingling, fatigue, and other (to be specified) feelings experienced during the stimulation session. Responses are provided on a 5-point Likert scale (1 = “*None*”; 5 = “*A lot*”). A further item investigates how much the feelings impacted the performance. Responses are provided on a 5-point Likert scale (1 = “*Not at all*”; 5 = “*Very much*”). Finally, the last item asked participants whether they believed they had received sham or beta stimulation (dichotomous response). 

### 2.5. Analysis

A summary of the analyzed endpoints, alongside the constructs they are intended to measure and the task they refer to, is presented in Table 1, before Section 3. The markdowns of the analyses can be found at Files S2 to S8, in the Supplementary Materials.

#### 2.5.1. Blinding Success

For the analysis of physical feelings, a composite score of the seven items was used as an indicator of the intensity of the distress caused by the stimulation. The level of reported impairment was coded as a separate, continuous variable. The presence/absence of phosphenes was coded as a dichotomic variable based on whether they were reported on the “other” option of the questionnaire. 

A binomial test indicated that the overall proportion of correct guesses was significantly greater than chance, *p* = <0.001, 95% CI [0.619, 0.833], with a proportion of correct responses of 0.736. This suggests that participants were able to distinguish active from sham sessions, probably because of the higher physical feelings induced by the real stimulation. Therefore, the session that participants believed they had been assigned to was coded as a dichotomous variable and controlled for in subsequent analyses to account for potential placebo/nocebo effects.

Participants experienced a very small amount of aversive sensations. The median response for any item measuring aversive sensations in the active stimulation condition was the first step of the Likert scale (“*None*”), while mean ratings to these items always stayed below the second step (range: 1.00—“*None*” for Pain (*SD* = 0), to 1.53 for Tingling (*SD* = 0.736)), and 30.6% and 13.9% of participants experienced phosphenes respectively in the beta and sham condition. However, such sensations could still impact participants’ performance by distracting form the task, raising the cognitive load, or, in the case of phosphenes, impairing visual processing; therefore, they were addressed in the robustness analysis (cf. Section 2.5.8).

#### 2.5.2. Mental Time Travel: Reaction Times

For the analysis of response times (RTs), only correct trials were retained. Additionally, due to task complexity, trials with extremely short latencies (<500 ms, *N* = 3) were considered anticipatory responses and excluded from analysis. The RT distribution was markedly non-normal (skewness = 1.849; kurtosis = 4.376; Shapiro–Wilk *W* = 0.84, *p* < 0.001). To address this violation, an inverse transformation was applied, which improved distributional characteristics (skewness = 0.135; kurtosis = −0.397; *W* = 0.99, *p* < 0.001). Subsequent removal of outliers, identified using the Tukey method, led to further improvement in normality (skewness = −0.002; kurtosis = −0.687; *W* = 0.99, *p* < 0.001). The residual significance of the Shapiro–Wilk test likely reflects the large sample size, which increases sensitivity to minimal deviations from normality. Prior to model specification, the distance of the events from the reference points was log-transformed after adding one unit due to non-normal distribution (skewness = 1.588; kurtosis = 3.690; *W* = 0.87, *p* < 0.001), resulting in satisfactory distributional improvements (skewness = −0.516; kurtosis = 0.005; *W* = 0.96, *p* < 0.001). For instance, when the participant self-projected to 2014 and a target event happened in 2011, the absolute temporal distance between the two is 3. A constant of 1 was added to this value to avoid logarithmic transformation of zeros, which could happen in case of personal events, and it was log-transformed, so 3 would become *log*(4) ~ 0.602. The average arousal rating distribution approximated normality (skewness = 0.028; kurtosis = −0.798; *W* = 0.97, *p* < 0.001) and was not transformed.

RTs were analyzed using a linear mixed-effects model to account for both fixed and random sources of variance. Random intercepts and slopes for event type (personal vs. impersonal) were included for each participant, capturing individual differences in baseline speed and sensitivity to event type. Fixed effects included: (i) the temporal reference point from which the event was judged (coded categorically: 2014, 2024, 2034), (ii) the log-transformed absolute temporal distance from the reference point; (iii) average across-subjects arousal rating for each event, and (iv) stimulation condition (sham vs. beta), (v) the guess the participants made about the stimulation condition. These predictors were selected based on prior theoretical and empirical work on mental time travel. The (v) predictor was added to partially out possible placebo/nocebo effects, since the results shown in Section 2.5.1 indicated that some participants were aware of the type of stimulation session.

Three nested models were fitted and compared using maximum likelihood estimation: (1) a baseline model including only covariates; (2) a model including also the main effect of stimulation condition; and (3) a model including the interaction between stimulation condition and reference point. For the best-fitting model, ANOVA was used to test parameter significance, and marginal means were computed and contrasted at *post hoc*, using a Bonferroni correction when necessary.

#### 2.5.3. Mental Time Travel: Accuracy

For accuracy analysis, all trials were retained. Omitted responses (i.e., responses not provided within the 5 s response window) were coded as incorrect. Given the extremely low error rate (0.83%), a simplified random effect structure was implemented to prevent model specification problems, including random intercepts for event type nested within each participant.

Accuracy was analyzed using generalized linear mixed-effects models with a logit link function and the same fixed effects as the RT analysis: (i) temporal reference point, (ii) absolute temporal distance from the reference point, (iii) average arousal rating, (iv) stimulation condition, and (v) the participant’s guess about their stimulation condition. Temporal distance values were again not normally distributed (skewness = 0.854; kurtosis = 3.813; *W* = 0.94, *p* < 0.001) and therefore log-transformed (skewness = −0.472; kurtosis = −0.117; *W* = 0.96, *p* < 0.001). Average arousal rating required no transformation (skewness = 0.033; kurtosis = −0.813; *W* = 0.97, *p* < 0.001).

Model comparison followed the same three-step nested approach using maximum likelihood estimation, with significance testing and post hoc contrasts conducted using the same procedures as RT analysis.

#### 2.5.4. Intertemporal Choice: Discounting Steepness

Indifference points were computed for each participant, session, maximum offer and delay combination as the average between the last rejected small-but-sooner and the last accepted smaller-but-sooner monetary amount of money, following standard procedure [58]. For each maximum offer, indifference points across delays were used to fit hyperbolic discounting curves [59], with the *k* parameter retained as the discounting steepness indicator.

Due to non-normal distribution (skewness = 8.164; kurtosis = 83.585; *W* = 0.28, *p* < 0.001), the k parameter was log-transformed to achieve normality (skewness = 0.040; kurtosis = −0.237; *W* = 0.99, *p* = 0.65). Discounting steepness was analyzed using a linear mixed-effects model with maximum offer included as a categorical fixed effect, given its known influence on discounting steepness [60]. Due to reduced trial numbers per condition, only random intercepts for participants were included to prevent model singularity. Finally, since the relationship between maximum offer levels and the discounting parameter cannot be assumed to be strictly linear, but at the same time is monotonous and unlikely to follow higher-order polynomial trends, we opted for ordered factor coding with a first-degree (linear) polynomial contrast. The participant’s guess about their stimulation condition was not included as a covariate, since the task is not performance-based and placebo/nocebo effects do not apply. Two models were estimated and compared: (1) a baseline model including only the covariate and (2) a model including also the main effect of stimulation condition. For the best model, an ANOVA omnibus was used to test the significance of the parameters, and marginal means were computed and contrasted at post hoc.

#### 2.5.5. Prospective Memory: Accuracy

Each interval between two consecutive button presses constituted a trial or “cycle”. The 2 min target time was considered correctly detected when the participant pressed the button within a ±6 s range, consistent with previous literature [18]. Response timing distribution ranged from 0.02 to 4.05 min, with first and third quartiles of 1.85 and 2.07 min, respectively (IQR/range = 0.05). Such presence of extreme outliers, potentially reflecting PM task neglect (extremely late responses) or instructions misunderstanding or unwanted pressures (extremely early responses), necessitated outlier removal using the Tukey method. This procedure retained trials ranging from 18.66 s before to 20.76 s after the 2 min target time. One participant was excluded due to the resulting absence of valid trials in one condition. After the procedure, 123 trials remained across 26 participants.

Response accuracy was analyzed using generalized linear mixed-effects model with a logit link function.

The correct response rate was analyzed using a generalized linear mixed-effects model to account for both fixed and random sources of variance, using the logit as a link function. Random intercepts were included to capture individual baseline accuracy differences. Fixed effects included (i) log-transformed number of clock checks prior to responding, (ii) the stimulation condition and (iii) the participant’s guess about their stimulation condition. Two models were compared: (1) baseline (covariate only) and (2) stimulation condition plus covariate. For the best-fitting model, an ANOVA omnibus was used to test the significance of the parameters, and marginal means were computed and contrasted at post hoc.

Continuous accuracy was assessed using the absolute deviation from the 2 min target for all trials, for both correct and incorrect trials. Due to the asymmetric, non-normal distribution, a Box–Cox transformation was applied to achieve normality (λ = 0.24; post-transformation: skewness = −0.019, kurtosis = −0.133, *W* = 0.99, *p* = 0.481). Continuous accuracy was analyzed using the same procedure as dichotomic accuracy analysis, but with a linear instead of a generalized linear model.

#### 2.5.6. Prospective Memory: Clock-Checking Behavior

Clock-checking behavior was analyzed for trials whose both check timing and button press timing met the Tukey criterion (excluding those >18.48 s before or >20.34 s after the target), resulting in 123 cycles across 26 participants.

Average check timing was modeled using linear mixed-effects models with subject-level random intercepts and fixed effects for (i) the stimulation condition and (ii) the temporal section at which the clock check happened: first (0–29 s), second (30–59 s), third (60–89 s), and fourth (>90 s) portions of each cycle. The temporal sections were coded as levels of a nominal variable. An interaction model was also used to investigate whether some effect of (iii) stimulation condition was visible only in some temporal regions. As a covariate in all models, (iv) the participant’s guess about their stimulation condition was included.

Check timing values were normalized (0–1) and logit-transformed due to non-normal distribution (pre-transformation: skewness = −0.524, kurtosis = −0.816, *W* = 0.94, *p* < 0.001; post-transformation: skewness = 0.164, kurtosis = 0.997, *W* = 0.99, *p* < 0.001). Since the distribution of the check timing was not normal (skewness = −0.524; kurtosis = −0.816; *W* = 0.94, *p* < 0.001), the values were normalized between 0 and 1 and logit-transformed. The normality of the resulting distribution improved sensibly (skewness = 0.164; kurtosis = 0.997; *W* = 0.99, *p* < 0.001).

Check frequency was analyzed using generalized linear mixed-effects models with Poisson distribution and log link function, comparing null (random intercepts only) and stimulation condition models. For the best-fitting model, an ANOVA omnibus was used to test the significance of the parameters, and marginal means were computed and contrasted at post hoc.

#### 2.5.7. Task Performance and Personality

Composite scores from the CAT questionnaire were computed for each participant by item score summation. Subject-level Self-Assessment Manikin (SAM) ratings yielded six: individual average ratings of both valence and arousal for past and future events (4 indices), plus the difference in ratings attributed to past and future events, normalized, computed as (*M_future_* − *M_past_*)/(*M_future_* + *M_past_*). While the former four indices define how activating or positively valenced past/future events used in the MTT task are, these latter two indices indicate how a participant is selectively more activated or prone to consider positively past or future events.

Cognitive subject-level variables were the same as described as task endpoints in the previous variables, namely: (a) MTT task—logit accuracy and average inverse reaction times for non-outlier trials; (b) DD task—average log-transformed k parameter; (c) PM task—average log-transformed absolute error, average check timing, and total check count for non-outlier trials. However, since this interest lies in trait-like interindividual differences, such cognitive indices are where computers are across stimulation conditions (namely, aggregating or averaging trials from both sham and active conditions).

Spearman correlation coefficients were calculated between participant-level performance indices, CAT and SAM scores. For the Spearman correlation coefficients computation, the pairwise-complete observations method. Corresponding *p*-values were estimated on listwise-complete cases. Given the exploratory nature of this specific analysis, aimed at identifying the direction and strength of potential associations between the scores of a new scale and non-personality variables, many comparisons were included, and no FDR correction was applied, and the results are discussed and interpreted as potential areas of further deepening.

#### 2.5.8. Robustness Analysis and Correction for Multiple Comparisons

In order to account for possible confounders, a robustness analysis was conducted using the same model specifications and variable inclusion as in the aforementioned task-by-task analysis, but including several additional potential confounders: session order (first vs. second), task order (MTT task first vs. DD and PM tasks first), reference-point order (six possible permutations of the Past, Present, and Future reference points within the MTT task), and self-reported side effects of stimulation, namely itching, pain, burning, tingling, fatigue, other sensations, and presence of phosphenes. Additionally, a False Discovery Rate (FDR) correction was applied to the results of the marginal means comparisons across tasks for the main endpoints reported in Table 1.

**Table 1:** The tasks involved in the experiment, the cognitive construct they are intended to tackle, and the operationalization of such constructs.

Task	Cognitive Construct	Decription of the Measure	Operationalization
Mental Time Travel (MTT)	Temporal ordinality judgments	The accuracy with which participants judge the temporal position of an event	Logit of the proportion of correct judgments
		The speed with which participants judge the temporal position of an event	Response latency (inverse RT, 1/RT)
Delay Discounting (DD)	Preference for sooner-but-smaller vs. greater-but-later rewards	Discounting steepness of value over time	Steepness (*k* parameter) of the curve describing the decrease in perceived value as a function of delay
Time-based Prospective Memory (PM)	Maintenance of intentions, prospective duration estimation	Whether participants successfully executed the intended delayed action within the correct temporal window	Whether the key was pressed within the 2 min ± 6 s window
		How precisely close to the target time participants executed the intended delayed action	Absolute deviation of the key-press timing from the 2 min target
	Monitoring strategy	How often participants checked elapsed time while waiting to execute the action	Total count of time checks
		How close to the target time window for action execution participants tended to check the time	Average timing of the time checks

## 3. Results

### 3.1. Mental Time Travel: Reaction Times

Likelihood ratio tests revealed that adding the stimulation condition effect (beta active stimulation versus sham stimulation) did not significantly improve model fit compared to the null model (*χ*^2^(1) = 3.22, *p* = 0.073). Therefore, the null model was retained for subsequent analyses. This model explained 25% of the variance in reaction times, while fixed effects alone explained 5% of the variance. The ANOVA omnibus highlighted significant effects for absolute distance of the event from reference *χ*^2^(1) = 231.53, *p* < 0.001; projection point, *χ*^2^(2) = 52.65, *p* < 0.001; and guessed condition, *χ*^2^(1) = 11.57, *p* = 0.001. No significant effect was observed for arousal, *χ*^2^(1) = 1.361, *p* = 0.243. Examination of estimated marginal means for guessing indicated a faster response when participants believed they were in the active condition (*M_Beta_* = 0.768, 95% CI [0.729, 0.807]) compared with sham (*M_Sham_* = 0.741, 95% CI [0.701, 0.781]), z = 3.398, *p* < 0.001. These results suggest that beta stimulation neither improved nor reduced overall speed.

### 3.2. Mental Time Travel: Accuracy

**Figure 2:**
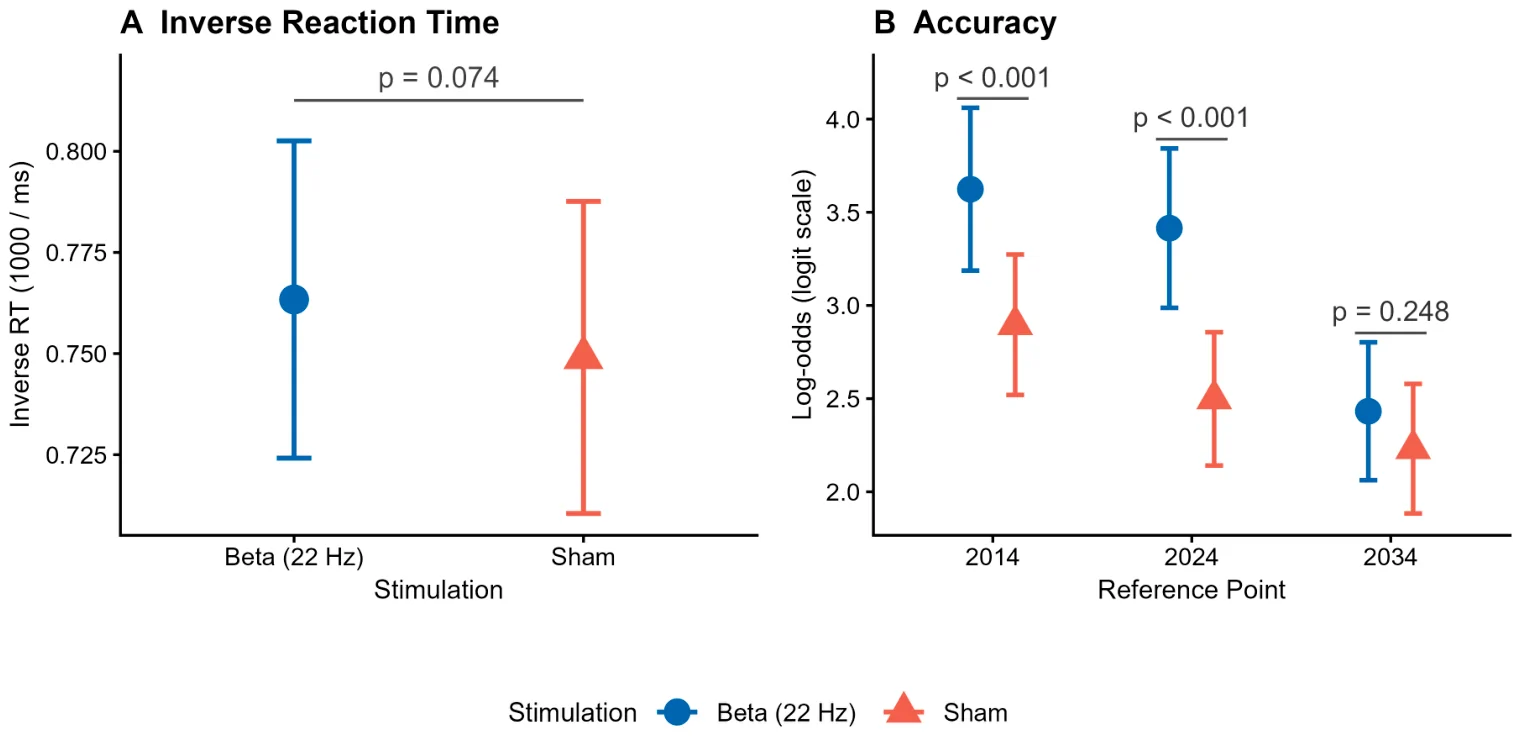
Mental time travel task—key findings. Panel (**A**): Estimated marginal means and confidence intervals for inverse reaction times at the mental time travel task for each stimulation condition. Under active (22 Hz) stimulation, participants tend to respond faster. Panel (**B**): Estimated marginal means and confidence intervals for log-odds of making a correct judgment at the mental time travel task, for each stimulation condition. Under active (22 Hz) stimulation, the likelihood of correct response increases for stimuli judged from the 2014 and 2024 reference points, while the difference is not significant for judgments given from the 2034 reference year. For all panels, error bars represent 95% confidence intervals. Distributions of individual data points within each condition can be found in File S9, in the Supplementary Materials.

Likelihood ratio tests revealed that adding the stimulation condition effect significantly improved model fit compared to the null model (*χ*^2^(1) = 20.17, *p* < 0.001); also, including the stimulation condition × reference point interaction significantly improved fit beyond the mere addition of the condition effect alone (*χ*^2^(2) = 8.89, *p* = 0.012). Therefore, the interaction model was retained for subsequent analyses. This model explained 27% of the variance in accuracy, while fixed effects alone explained 9% of the variance. The ANOVA omnibus test revealed the following significant effects: stimulation (*χ*^2^(1) = 11.82, *p* < 0.001), stimulation condition × reference point interaction (*χ*^2^(2) = 9.11, *p* = 0.010), reference point (*χ*^2^(2) = 46.63, *p* < 0.001), arousal (*χ*^2^(1) = 5.51, *p* = 0.019), guess (*χ*^2^(1) = 4.76, *p* = 0.029) and log-transformed absolute temporal distance from the reference point (*χ*^2^(1) = 89.58, *p* < 0.001). Post hoc contrasts revealed that beta stimulation did not significantly improve accuracy compared to sham only for the 2034 reference point (*Log-OR*_2034_ = 0.201, *SE* = 0.174, *z* = 1.155, *p* = 0.248), while significant improvements were observed for the 2014 (*Log-OR*_2014_ = 0.727, *SE* = 0.212, *z* = 3.438, *p* < 0.001) and 2024 (*Log-OR*_2024_ = 0.916, *SE* = 0.206, *z* = 4.446, *p* < 0.001) reference points. These results suggest that beta stimulation selectively improved accuracy when events were judged based on the present or past as a reference point, indicating temporal context-dependent modulation of processing. The result proved robust to the inclusion of additional extensive control variables (Past: *p* = 0.011; Present: *p* < 0.001; Future = *p* = 0.559) and survived the FDR correction (Past: *p* = 0.001; Present: *p* < 0.001; Future = *p* = 0.298). The results of the MTT analysis are illustrated in Figure 2.

### 3.3. Intertemporal Choice: Discounting Steepness

Likelihood ratio tests revealed that adding the stimulation condition (beta active stimulation versus sham stimulation) effect significantly improved model fit compared to the null model (*χ*^2^(1) = 4.03, *p* = 0.045). Therefore, the main effects model was retained for subsequent analyses. This model explained 76% of the variance in discounting steepness, while the fixed effects alone explained 10% of the variance. The ANOVA omnibus test highlighted the following significant effects: stimulation condition (*χ*^2^(1) = 4.03, *p* = 0.045) and maximum reward (*χ*^2^(1) = 81.26, *p* < 0.001). Marginal means in the two stimulation conditions showed higher log-transformed *k* for the beta (*M_beta_* = −2.67, *SE* = 0.369) compared to the sham (*M_sham_* = −3.02, *SE* = 0.369) condition, with a significant difference of 0.345 (*SE* = 0.172, *t*(163) = 2.01, *p* = 0.046, *d* = 0.29).

Given that higher *k* values indicate greater discounting and the analysis was conducted on log-transformed *k* values, these results would suggest that beta stimulation increased temporal discounting steepness compared to sham stimulation, with this effect being consistent across different maximum reward magnitudes. However, such an effect did not survive either the inclusion of extensive control variables (*p* = 0.08) nor the FDR correction (*p* = 0.06); therefore, there is likely no effect of beta stimulation of delay discounting.

**Figure 3:**
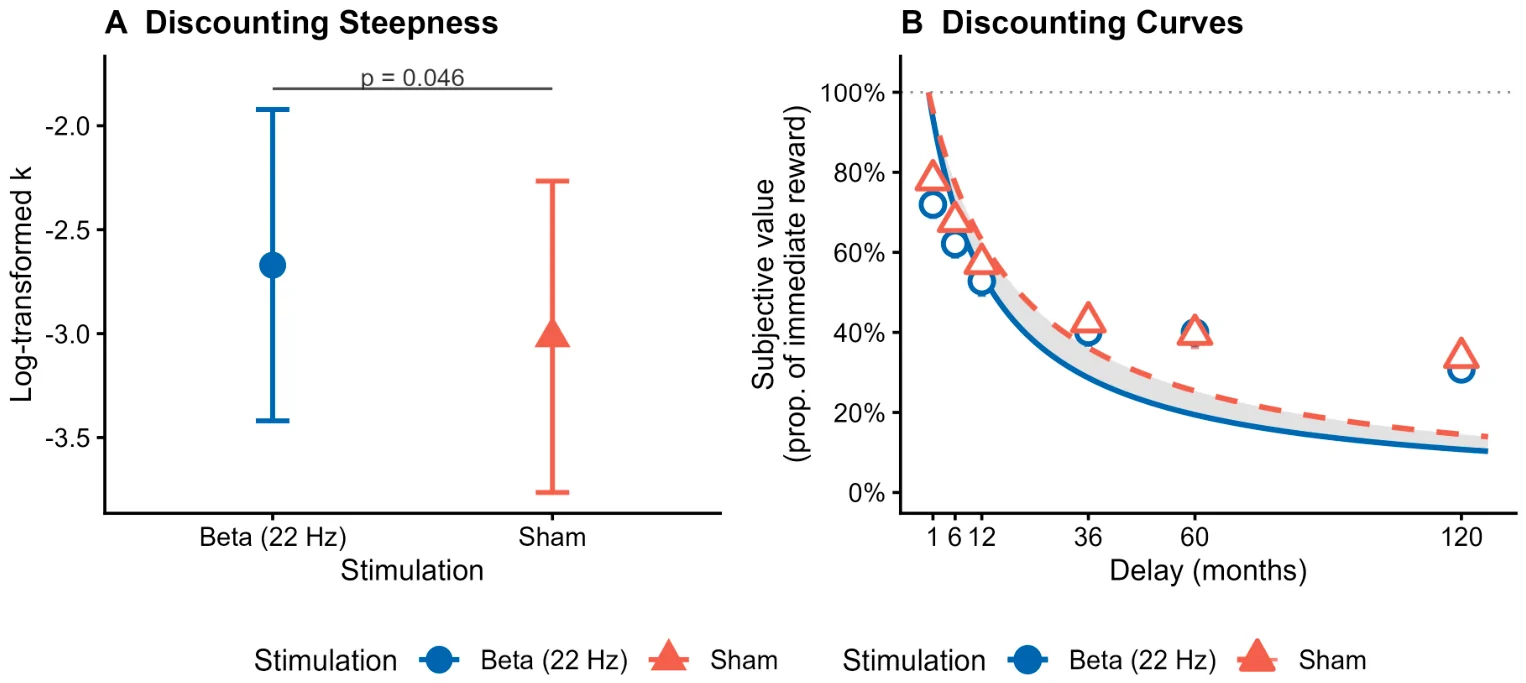
Delay discounting task—key findings. Panel (**A**): Estimated marginal means and confidence intervals for delay discounting rates *k* at Intertemporal Choice for each stimulation condition. Under active (22 Hz) stimulation, participants tend to exhibit a stronger preference for present rewards (namely, higher *k* values). Panel (**B**): Indifference points as a percentage of the total reward for each delay mount, for both stimulation conditions. At almost all time points, the indifference point is higher under Sham rather than active (22 Hz) stimulation; such a plot is purely for visualization purposes and does not represent a delay-by-delay formal analysis. For all panels, error bars represent 95% confidence intervals. Distributions of individual data points within each condition can be found in File S9, in the Supplementary Materials.

The results of the DD analysis are illustrated in Figure 3.

### 3.4. Prospective Memory: Accuracy

With respect to dichotomous accuracy, model comparison revealed that adding the main effect of stimulation to the null model—which included only the log-transformed number of clock checks and participants’ guesses about their stimulation condition—provided the best fit to the accuracy data (*χ*^2^(1) = 9.37, *p* = 0.002). This model explained 54% of the variance in the accuracy, while the number of clock checks alone explained 43% of the variance. The final model showed a significant main effect of stimulation condition, *χ*^2^(1) = 7.96, *p* = 0.005, of the participant’s guess, *χ*^2^(1) = 8.64, *p* = 0.003. There was also a positive effect of clock-checking behavior on accuracy (*β* = 2.55, *SE* = 0.74, *z* = 3.43, *p* < 0.001), indicating that participants who engaged in more frequent time monitoring were significantly more likely to respond within the target window. Interestingly, the post hoc analysis revealed that participants achieved more correct trials when they believed they were in the sham condition (*M_diff_* = −2.58, *SE* = 0.879, *z* = 2.94, *p* = 0.003), although (on the contrary) they had a better performance under beta stimulation than under sham stimulation (*M_diff_* = 2.14, *SE* = 0.757, *z* = 2.821, *p* = 0.005). Such an effect proved robust to the inclusion of extensive control variables (*p* = 0.015) and survived the FDR control (*p* = 0.007).

Continuous accuracy was assessed using absolute deviation from the 2 min target for all trials (both correct and incorrect). Model comparison revealed that adding the main stimulation effect to the null model significantly improved fit (*χ*^2^(1) = 6.49, *p* = 0.011). The final model accounted for 40% of the variance, while the fixed effects accounted for 19% of the variance. The model showed a significant negative relationship between clock-checking frequency and timing error (*β* = −0.085, *SE* = 0.026, *t* = −3.23, *p* = 0.001), indicating that increased monitoring behavior was associated with reduced temporal deviation from the target, while the ANOVA omnibus highlighted a significant effect of both stimulation condition, *χ*^2^(1) = 6.42, *p* = 0.011, and participant’s guess of their stimulation condition, *χ*^2^(1) = 7.88, *p* = 0.005. The post hoc analysis revealed that participants under sham stimulation deviated more from the 2 min target than under beta stimulation (*M_diff_* = −0.067, *SE* = 0.027, *z* = −2.821, *p* = 0.014), and that they deviated more when they believed they were in the beta condition (*M_diff_* = −2.58, *SE* = 0.879, *z* = 2.94, *p* = 0.003). Such an effect proved robust to the inclusion of extensive control variables (*p* = 0.03) and survived the FDR control (*p* = 0.019).

### 3.5. Prospective Memory: Clock-Checking Behavior

**Figure 4:**
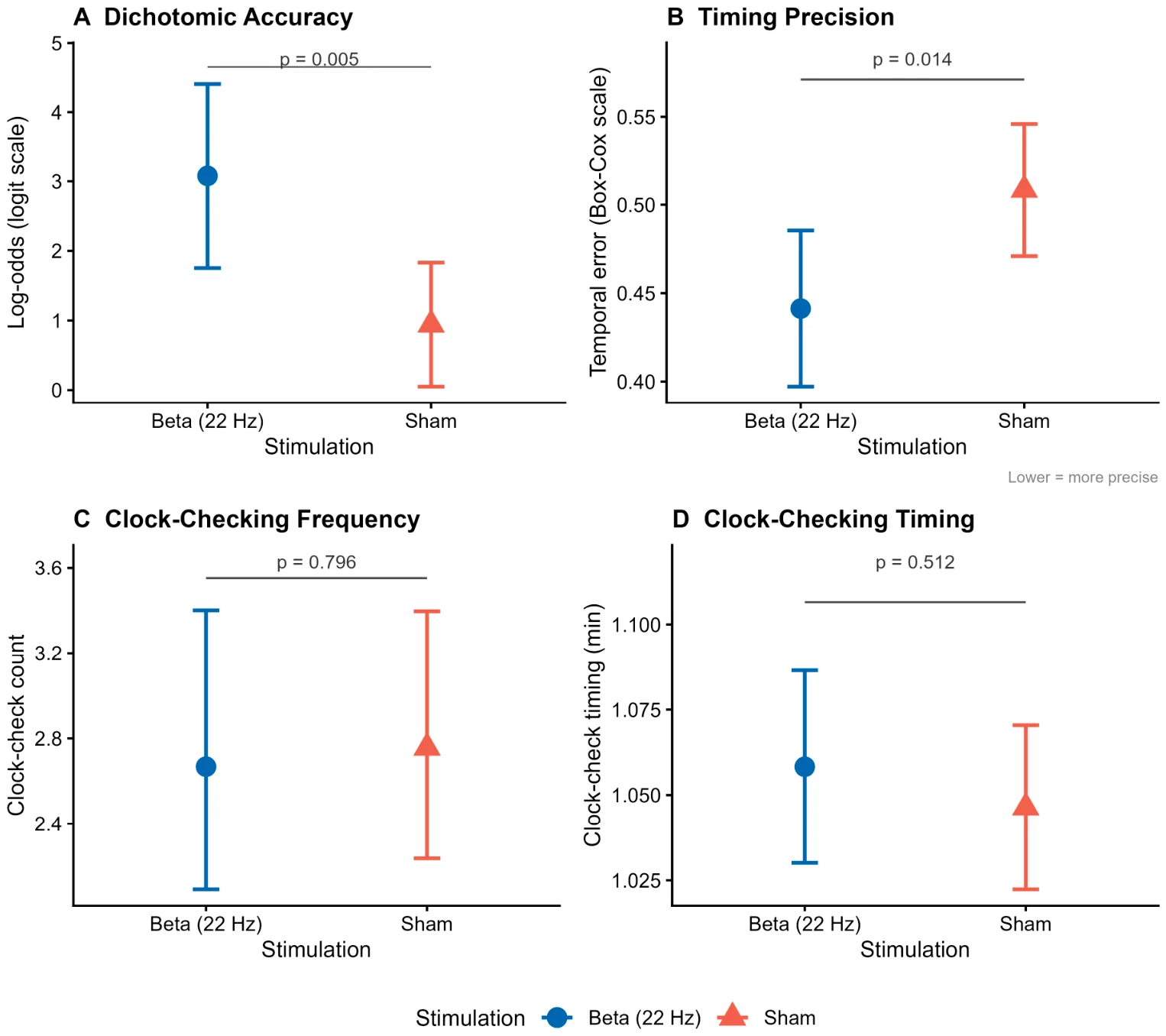
Time-based prospective memory task—key findings. Panel (**A**): Estimated marginal means and confidence intervals for the log-odds of pressing the button within the target window (2 min ± 6 s) in the time-based prospective memory task. Participants more often performed correctly under active (22 Hz) stimulation. Panel (**B**): Estimated marginal mean of the deviation from the target 2 min interval. Under active (22 Hz) stimulation, participants tended to make more precise judgments. Panel (**C**): Estimated marginal means and confidence intervals for the number of clock checks. No significant difference was found between conditions. Panel (**D**): Estimated marginal means and confidence intervals for the timing of clock checks. No significant difference was found between conditions. For all panels, error bars represent 95% confidence intervals. Distributions of individual data points within each condition can be found in File S9, in the Supplementary Materials.

In respect to check frequency, model comparison revealed that the null model, which included only temporal quartiles as predictors, was preferable to the model including stimulation condition. This model explained 50% of the variance, with the main effect of time period (*χ*^2^(3) = 65.76, *p* < 0.001) explaining 20% of the variance. Specifically, marginal means estimation on contrast highlighted significant differences between the fourth and the first (*M_diff_* = −0.82, *SE* = 0.15, *z* = −5.58, *p* < 0.001), second (*M_diff_* = −0.75, *SE* = 0.12, *z* = −6.52, *p* < 0.001) and third (*M_diff_* = −0.54, *SE* = 0.11, *z* = −5.20, *p* < 0.001) quartiles, while no other contrast proved significant. Linear mixed-effects analysis confirmed no significant effect of stimulation condition on the timing of checking behavior (*χ*^2^(1) = 0.443, *p* = 0.506). The final model revealed the expected pattern whereby check timing significantly increased across quartiles (*F*(3) = 6261.29, *p* < 0.001), with mean check times systematically progressing from early (*M*_Q1_ = 0.34, *SE* = 0.022) to later portions of each cycle (*M*_Q4_ = 1.83, *SE* = 0.011). The absence of stimulation effects suggested no systematic shift in checking behavior, either globally or within specific temporal sectors. The results of the PM analysis are illustrated in Figure 4.

### 3.6. Task Performance and Personality

Across-stimulation condition faster (higher inverse reaction times) and more accurate MTT processing, as well as discounting rates, were not significantly associated with personality dimensions. The strongest correlations for them were respectively with Future Anxiety (FA; ρ = −0.262, *p* = 0.226), Present Resilient (PR; ρ = 0.376, *p* = 0.077), and Present Hedonistic (PH; ρ =0.364, *p* = 0.087). The timing error (absolute deviance from the target time) significantly correlated with Present Resilient (PR; ρ = −0.465, *p* = 0.025) and Present Eudaimonic (PE; ρ = −0.422, *p* =0.045). On the other hand, the number of clock checks was significantly correlated with Future Proactive (FP; ρ = 0.203, *p* = 0.014).

Self-Assessment Manikin ratings for the MTT events to be judged revealed systematic relationships between emotional temporal perception and CAT dimensions. Valence ratings for future events correlated most strongly with Present Eudaimonic (PE; ρ = 0.532, *p* = 0.009) and Past Nostalgic (PNo; ρ = −0.449, *p* = 0.032), while arousal ratings for future events correlated with Present Hedonistic (PH; ρ = 0.418, *p* = 0.047).

**Figure 5:**
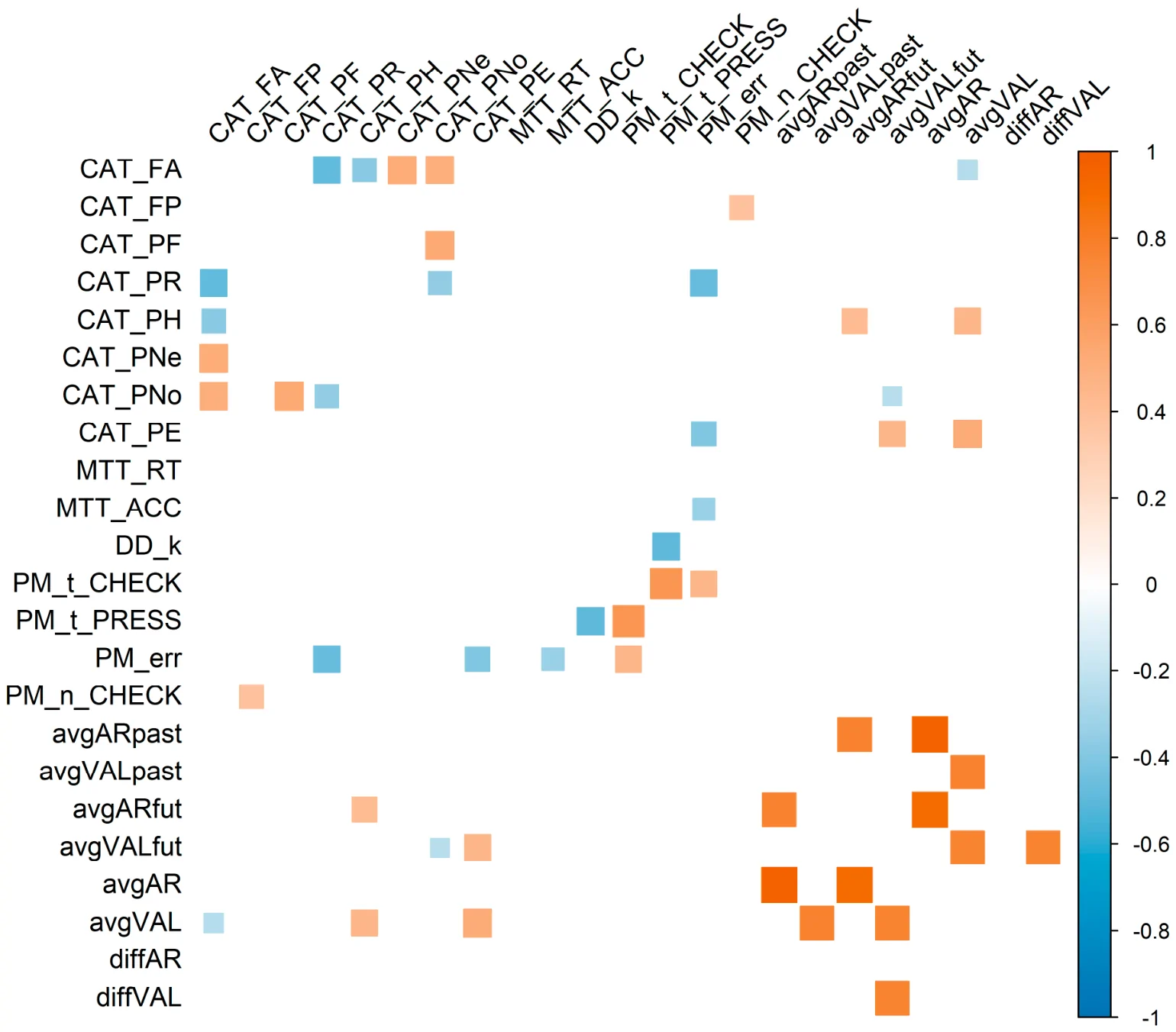
Correlation between task performance and personality measures. Correlations between performance at the mental time travel, delay discounting and prospective memory tasks, personality (CAT Questionnaire) and attitudes (SAM) measures. The size of the squares represents the absolute value of the correlation; empty spaces are left in place of non-significant associations. CAT: Control-Affect-Time Model of Time Perspective Questionnaire, of which FA = Future Anxiety; FP = Future Proactive; PF = Present Fatalistic; PR = Present Resilient; PH = Present Hedonistic; PNe = Past Negative; PNo = Past Nostalgic; PE = Present Eudaimonic. MTT: mental time travel task, of which RT = inverse reaction times; ACC = logit of accuracy. DD: delay discounting task, of which k = discounting rate. PM: prospective memory task, of which t_CHECK = timing of clock-checks; t_PRESS = timing of button press; err = timing error at button press; n_CHECK = count of clock-checks. avgARpast/avgARfut = average arousal ratings for past and future events, respectively, at the Self-Assessment Mannikin; avgVALpast/avgVALfut = average valence ratings for past and future events, respectively, at the Self-Assessment Mannikin; diffAR/diffVAL = normalized differences between arousal/valence ratings for past vs. future events.

Finally, in relation to correlations across tasks, accuracy to the MTT task and timing errors at the time-based PM were significantly inversely correlated (ρ = −0.446, *p* = 033), while the discounting rate was correlated with an earlier pressing of the target button at time-based PM (ρ = −0.475, *p* = 023). The complete correlation table is depicted in Figure 5.

## 4. Discussion

The present study showed a significant association of prefrontal beta oscillations with two of the temporal cognition tasks—mental time travel (MTT) and time-based prospective memory (time-based PM)—examined by applying 22 Hz transcranial alternating current stimulation (beta-tACS) over bilateral DLPFC regions. Moreover, individual differences in time perspective were found to be associated with performance across temporal tasks.

The most relevant effect concerns the robust tACS-associated improvement in prospective memory accuracy. Both dichotomous accuracy measures and continuous temporal precision measures showed significant improvements, indicating that stimulation was associated with an improved capability to either (a) keepg track of the time (temporal information extraction) and/or (b) engage in a dual task (temporal information utilization). This could be a possible consequence of the enhanced working memory maintenance and distraction inhibition function of the DLPFC [53,61,62].

Clock-checking frequency and timing, on the other hand, were not sensible to stimulation, meaning that such a difference in prospective memory performance should indeed not be imputed to a different metacognitive strategy selection. Namely, in the TWTE model [9], beta oscillations are not linked to a change in the dynamic between the ‘wait’ and ‘test’ phases but rather to the decisional exiting phase.

On one hand, these results align with and provide experimental support for the findings of Santacesaria and colleagues [18], which identified an increase in high-beta DLPFC activity specific to time-based prospective memory (time-based PM) rather than event-based prospective memory (event-based PM), as well as with the AtoDI model [10], which highlights the role of the DLPFC in maintaining delayed intentions. On the other hand, these results align with the Maintenance Theory [63] and the “beta-as-status-quo” hypothesis [53], which state that beta oscillations support the maintenance of stable cognitive representations, a function often associated with sustaining the current cognitive set or “status quo”. Since the effect on PM is observed in the temporal information utilization rather than in the monitoring strategy variables, the putative role of beta oscillations could be of maintenance of the delayed intention and the available temporal information in the face of the distraction due to a concomitant task (the DD task).

A second result is that beta-tACS in the bilateral DLPFC affected participants’ performance at MTT. Indeed, there was an improvement in the MTT performance accuracy. Specifically, when participants were asked to judge the ordinality of the event in relation to a past and present (2014, 2024) but not future (2034) temporal reference point. In the context of the MTT task, participants needed to maintain a temporal anchor in working memory and compare events relative to that reference. Always in line with Maintenance Theory, enhancing beta activity in the DLPFC may therefore have facilitated the top-down maintenance and manipulation of temporal reference frames [10], improving temporal comparison accuracy only when the reference point corresponded to an existing or well-established temporal context (past or present). The maintenance of the self-projection needed for reference could in fact rely on pre-existing anchors. In contrast, judgments involving a future reference point may rely more heavily on constructive episodic simulation processes, which are thought to depend on hippocampal-default mode network interactions rather than primarily on prefrontal maintenance mechanisms [64]. Consequently, modulating beta activity in the DLPFC may preferentially enhance processes related to temporal anchoring, while having limited impact on the generative mechanisms involved in projecting oneself into the future.

Another possible interpretation is that DLPFC beta-tACS modulated the position of mental projections along a past-future gradient within the mental time map. However, previous work has suggested that DLPFC activity during mental time travel reflects primarily a west–east spatial gradient, rather than a past-future temporal gradient, with the latter being more strongly associated with activity in regions such as the supramarginal gyrus and medial prefrontal cortex [28]. Therefore, the posterior-to-anterior gradient within the prefrontal cortex organizing the processing of future-oriented content and reflecting a shift from relatively concrete representations to increasingly abstract representations [2] would better explain these data without contradicting previous evidence. Within this framework, in fact, beta-tACS over the DLPFC may have preferentially enhanced the maintenance of more concrete temporal representations, such as past and present reference points, while having less impact on the more abstract representations required for future projections. This pattern of results across MTT and PM taks overall suggests that beta oscillatory activity over the DLPFC may not support future-oriented cognition indiscriminately but rather facilitates the maintenance and use of more concrete, temporally proximal representations.

Contrary to our starting hypothesis, beta-tACS was not associated with changes in temporal discounting, even though previous findings showed reduced beta activity in the lateral PFC during less impulsive (“greater-but-later”) choices, so it was hypothesized that DLPFC activation is negatively correlated with the perceived magnitude of delay [36,43,65].

Finally, concerning the relationship between task performance and the personality profile, several coherent associations emerged between CAT dimensions (reflecting time perspective attitudes) [52] and task performance, particularly in relation to temporal monitoring and affective representations of the future.

In the time-based PM task, timing accuracy (absolute deviation from the 2 min target) was significantly and negatively associated with Present Resilient and Present Eudaimonic. Individuals scoring higher on these dimensions showed more precise temporal estimation. This pattern is consistent with a more sustained and committed engagement with the task: Present Resilient may reflect a determined, effortful stance toward demanding activities, while Present Eudaimonic may index a more “present” involvement in ongoing behavior. The number of clock checks, on the other hand, was significantly associated with future-proactive orientation, suggesting that individuals with a stronger proactive future orientation engaged more frequently in strategic behaviors such as active time monitoring.

Across tasks, performance measures converged in theoretically meaningful ways. Accuracy at the MTT task and timing errors at the time-based PM task were inversely correlated, pointing to a shared component—possibly executive monitoring or temporal control—underlying both paradigms. In addition, higher discount rates were associated with earlier button presses in the time-based PM task, linking intertemporal choice tendencies and the underlying impulsiveness to anticipatory responding in prospective timing.

Clear relationships also emerged in the affective evaluation of future events. Valence ratings for future scenarios were positively associated with Present Eudaimonic and negatively with Past Nostalgic, suggesting that a meaning-oriented present focus supports a more positive construction of the future, whereas a stronger nostalgic orientation is linked (and can be hypothesized to be due) to less positive future appraisal. Arousal ratings for future events were positively correlated with Present Hedonistic, showing a more emotionally activated anticipation of future scenarios in individuals with a stronger present-focused, sensation-seeking profile. Overall, while basic speed and accuracy indices were largely independent of personality, measures tapping temporal precision, strategic monitoring, intertemporal preferences, and emotional future processing showed theoretically interpretable associations with specific CAT dimensions.

### Limitations

Several methodological considerations should be noted when interpreting these findings.

First, the significant blinding failure (73.6% correct guesses) represents a methodological limitation that may have influenced results through placebo/nocebo effects. We tried to address such limitations through the inclusion of the perceived stimulation condition as a covariate in the analyses. Future studies should consider optimizing stimulation parameters to improve blinding while maintaining efficacy. Overall, there seems to be a pattern of nocebo effect, found in both the MTT and PM tasks, for which people believing to be undergoing beta-tACS seem to perform worse, an effect which counters the actual accuracy-boosting effects of the beta stimulation itself.

Second, the heterogeneity of temporal events (both impersonal and autobiographical), even though improving the generalizability of the result, introduces potential confounds that larger samples would better accommodate. Future personal events are inherently hypothetical and qualitatively distinct from past personal experiences or semantic historical events. Although this mixed-event design enhances ecological validity, it complicates interpretation of the mechanisms underlying observed effects. Designs focusing exclusively on either personal or semantic events could strengthen causal inferences, albeit at some cost to generalizability across event types.

Third, our sample of university students (aged 22–29) limits generalizability across the lifespan. While this narrow age range helped control relative temporal distances for personal events, it constrains applicability to broader populations. Future research could recruit more diverse age cohorts when examining personal events or employ standardized semantic events to trace developmental trajectories in temporal cognition.

Fourth, the compressed experimental protocol required by tACS time constraints reduced trial counts, particularly for the PM task. This limitation may have constrained our ability to detect meaningful, robust PM effects and restricted comprehensive within-subject analyses. Future studies could benefit from separating the MTT task from the DD/PM battery, allowing for increased trial sampling while preserving the integrity of each paradigm.

Fifth, the current tACS approach lacks the spatial specificity needed for definitive causal claims. Without a head model, neuronavigation or concurrent electrophysiological monitoring, we cannot confirm precise current delivery to target regions or exclude stimulation of neighboring areas. Given the relatively large surface of both the DLPFC and the sponges, it is reasonable to assume that the montage engaged in at least part of this region; at the same time, however, we cannot determine whether the observed effects reflect an exclusive modulation of DLPFC activity or a downstream/cascading effect on interconnected, or the effect of a spillover of electric current on proximal regions, such as those belonging to the dorsal attention network (DAN) or broader frontoparietal networks involved in executive control and attention. The associations emerging from data should therefore be considered a tentative corroboration of a possible causal involvement, pending further experimental evidence.

Sixth, although the inverse transformation improved the distributional characteristics of the RTs for the purposes of parametric tests, it does not allow us to distinguish between the contributions of the Gaussian component and the tail component to the RT distribution; approaches based on ex-Gaussian fitting [66] could provide a more complete picture of the underlying cognitive processes, and represent a promising direction for future analyses.

## 5. Conclusions

Beta-tACS at 22 Hz was associated with effects across two temporal cognitive domains: enhanced accuracy for ordinality judgements in mental time travel for all time frames except the future and improved prospective memory performance but not reduced temporal discounting. Taken together, the present findings provide possible evidence of the functional role of the DLPFC in supporting the maintenance of temporally relevant representations that guide behavior, but only when they are concrete and well-established. On the other hand, the idea of a general facilitating role in broader temporal cognition, or more specifically in future cognition, cannot be supported. Across tasks, DLPFC beta activity appears to contribute to the stabilization of temporal information within ongoing cognitive processing. In the mental time travel task, this mechanism may have facilitated the maintenance of concrete temporal reference points, improving judgments anchored in past and present contexts. Within the prospective memory paradigm, a similar process may support the sustained representation of delayed intentions and the monitoring of temporal cues necessary for their execution. More broadly, in line with the Goal Maintenance Theory [63], these results suggest that the DLPFC may act as a control hub that maintains and prioritizes temporally relevant representations in working memory, allowing individuals to coordinate present behavior with past information and future-oriented goals. Interestingly, individual differences in time perspective attitudes were found to be associated with performance across temporal tasks, measured irrespective of the stimulation condition.

## Data Availability

The subject-level data is not disclosed due to privacy agreements with participants. See Supplementary Materials for the code markdown.

## Supplementary Materials

The following supporting information can be downloaded at https://bit.ly/4tMenc3 and https://www.mdpi.com/article/10.3390/brainsci16090899/s1: File S1: Stimuli (table); File S2: Balancing (code markdown); File S3: Blinding (code markdown); File S4: Delay discounting (code markdown); File S5: Mental time travel (code markdown); File S6: Prospective memory (code markdown); File S7: Personality (code markdown); File S8: FDR correction (code markdown); File S9: Supplementary Figures (boxplots).

## Abbreviations

The following abbreviations are used in this manuscript:

(hd-)EEG	(High-Density) Electroencephalography
ACC	Anterior Cingulate Cortex
AtoDI	Attention to Delayed Intentions Model
CAT	Control-Affect-Time Model of Time Perspective
DD	Delay Discounting
DLPFC	Dorsolateral Prefrontal Cortex
EFT	Episodic Future Thinking
FEF	Frontal Eye Fields
MEG	Magnetoencephalography
MTT	Mental Time Travel
PM	Prospective Memory
Pre-SMA	Pre-supplementary motor cortex
rTMS	Repetitive Transcranial Magnetic Stimulation
tACS	Transcranial Alternating Current Stimulation
tDCS	Transcranial Direct Current Stimulation
tES	Transcranial Electric Stimulation
TP	Time Perspective
TWTE	Test-Wait-Test-Execute
ZTPI	Zimbardo Time Perspective Inventory
