# Supplementary material for: Beta-Band Stimulation of Dorsolateral Prefrontal Cortex Through tACS Alters Mental Time Travel and Prospective Memory, but Not Delay Discounting
Source: Brain Sci. 2026 Aug 22;16(9):899. doi: 10.3390/brainsci16090899 (PMC13604682; doi:10.3390/brainsci16090899)
Supplement: Supplementary file 1 [file brainsci-16-00899-s001.zip › S2 Balancing (code markdown).html]

tACS\_future\_BALANCING\_CHECK


# tACS\_future\_BALANCING\_CHECK

#### Authors are masked for peer review purposes

# UPLOADING DATA

The dataset `data_conditions` is a data frame (36 × 4)
with the following variables:

- `order_cond`: factor, 2 levels (1, 2) — sham first
  vs. beta first
- `order_task`: factor, 2 levels (1, 2) — MTT first vs. DD
  + tPM first
- `order_ref`: factor, 6 levels (123, 132, 231, 213, 312,
  321) — order of PAST, PRESENT and FUTURE reference points in MTT

# CHI TESTS TO CHECK COUNTERBALANCING:

# Equal distribution at single-factors level

```
table(data_conditions$order_cond) %>% 
  chisq.test(simulate.p.value = TRUE, B = 10000)
```

```
## 
##  Chi-squared test for given probabilities with simulated p-value (based
##  on 10000 replicates)
## 
## data:  .
## X-squared = 0.11111, df = NA, p-value = 0.864
```

```
table(data_conditions$order_task) %>% 
  chisq.test(simulate.p.value = TRUE, B = 10000)
```

```
## 
##  Chi-squared test for given probabilities with simulated p-value (based
##  on 10000 replicates)
## 
## data:  .
## X-squared = 1, df = NA, p-value = 0.3985
```

```
table(data_conditions$order_ref) %>% 
  chisq.test(simulate.p.value = TRUE, B = 10000)
```

```
## 
##  Chi-squared test for given probabilities with simulated p-value (based
##  on 10000 replicates)
## 
## data:  .
## X-squared = 0.66667, df = NA, p-value = 0.9935
```

# CHI TESTS TO CHECK COUNTERBALANCING:

# Independence of other conditions from stimulation order

```
table(data_conditions$order_cond, data_conditions$order_task) %>% 
  chisq.test(simulate.p.value = TRUE, B = 10000)
```

```
## 
##  Pearson's Chi-squared test with simulated p-value (based on 10000
##  replicates)
## 
## data:  .
## X-squared = 0.38532, df = NA, p-value = 0.7335
```

```
table(data_conditions$order_cond, data_conditions$order_ref) %>% 
  chisq.test(simulate.p.value = TRUE, B = 10000)
```

```
## 
##  Pearson's Chi-squared test with simulated p-value (based on 10000
##  replicates)
## 
## data:  .
## X-squared = 0.57638, df = NA, p-value = 1
```
