# Supplementary material for: Beta-Band Stimulation of Dorsolateral Prefrontal Cortex Through tACS Alters Mental Time Travel and Prospective Memory, but Not Delay Discounting
Source: Brain Sci. 2026 Aug 22;16(9):899. doi: 10.3390/brainsci16090899 (PMC13604682; doi:10.3390/brainsci16090899)
Supplement: Supplementary file 1 [file brainsci-16-00899-s001.zip › S3 Blinding (code markdown).html]

tACS\_future\_BLINDING\_CHECK


# tACS\_future\_BLINDING\_CHECK

#### Authors are masked for peer review purposes

# § 2.4.1. Physical feelings during stimulation and blinding success

## Libraries

```
library(readr)
library(readxl)
library(dplyr)
library(lubridate)
library(emmeans)
library(datawizard)
library(lme4)
library(tidyr)
```

## Loading data

```
load("~/PROJECTS/tacs_MTT_DD_PM/DATASETS/DATA_SENSORY_GUESS.RData")
```

## Setting seed

```
set.seed(254364673)
```

## Blinding Check

```
tab <- data_sensory %>%
  mutate(
    exp_cond = case_when(
      exp_cond == "beta" ~ "EXP_COND_IS_BETA",
      exp_cond == "sham" ~ "EXP_COND_IS_SHAM",
      TRUE ~ exp_cond),
    guess_cond = case_when(
      guess_cond == "beta" ~ "GUESS_IS_BETA",
      guess_cond == "sham" ~ "GUESS_IS_SHAM",
      TRUE ~ guess_cond)) %>%
  {table(.$exp_cond,.$guess_cond)}
  
print(tab)
```

```
##                   
##                    GUESS_IS_BETA GUESS_IS_SHAM
##   EXP_COND_IS_BETA            32             4
##   EXP_COND_IS_SHAM            15            21
```

```
success_all <- tab["EXP_COND_IS_BETA", "GUESS_IS_BETA"] + tab["EXP_COND_IS_SHAM", "GUESS_IS_SHAM"]
tot_all     <- sum(tab)
binom.test(success_all, tot_all, p = 0.5)
```

```
## 
##  Exact binomial test
## 
## data:  success_all and tot_all
## number of successes = 53, number of trials = 72, p-value = 7.556e-05
## alternative hypothesis: true probability of success is not equal to 0.5
## 95 percent confidence interval:
##  0.6189721 0.8330460
## sample estimates:
## probability of success 
##              0.7361111
```
