# Supplementary material for: Beta-Band Stimulation of Dorsolateral Prefrontal Cortex Through tACS Alters Mental Time Travel and Prospective Memory, but Not Delay Discounting
Source: Brain Sci. 2026 Aug 22;16(9):899. doi: 10.3390/brainsci16090899 (PMC13604682; doi:10.3390/brainsci16090899)
Supplement: Supplementary file 1 [file brainsci-16-00899-s001.zip › S4 Delay Discounting (code markdown).html]

tACS\_future\_Delay\_Discounting


# tACS\_future\_Delay\_Discounting

#### Authors are masked for peer review purposes

# PRELIMINARY

```
set.seed(57456)
```

`data_DD`: 1,188 obs × 7 variables — `id` (fct,
33 levels), `session` (1/2), `stimulation`
(beta/sham), `rew_max` (200/10000/40000 €),
`rew_delay` (months: 1/6/12/36/60/120),
`ind_point` (€), `ind_perc` (ind\_point /
rew\_max).

---

# DISCOUNT RATE ESTIMATION

Hyperbolic discounting curve fit per participant × session × reward
level (Green & Myerson, 1996). *k*: steepness parameter
(higher = greater impatience).

```
data_DD_subj_level <-
data_DD %>%
group_by(id, session, rew_max) %>%
summarize(
k = nls(ind_perc ~ 1/(1 + k * rew_delay), start = list(k = 0.01)) %>%
broom::tidy() %>% pull(estimate),
stimulation = first(stimulation),
.groups = "drop"
)
```

`data_DD_subj_level`: 198 obs × 5 variables —
`id`, `session`, `rew_max`,
`k`, `stimulation`.

---

# NORMALITY ASSESSMENT

```
sw_raw <- shapiro.test(data_DD_subj_level$k)

tibble(
Statistic = c("Skewness", "Kurtosis", "Shapiro-Wilk W", "Shapiro-Wilk p"),
Value = c(
round(skewness(data_DD_subj_level$k)$Skewness, 3),
round(kurtosis(data_DD_subj_level$k)$Kurtosis, 3),
round(sw_raw$statistic, 3),
format.pval(sw_raw$p.value, eps = .001))
) %>% kable(caption = "Normality diagnostics — raw k")
```

Normality diagnostics — raw k

| Statistic | Value |
| --- | --- |
| Skewness | 8.164 |
| Kurtosis | 83.585 |
| Shapiro-Wilk W | 0.28 |
| Shapiro-Wilk p | < 0.001 |

```
data_DD_subj_level <- data_DD_subj_level %>%
mutate(
k_trans = log(k),
rew_max_c = as.factor(ordered(rew_max, levels = c(200, 10000, 40000)))
)

sw_log <- shapiro.test(data_DD_subj_level$k_trans)

tibble(
Statistic = c("Skewness", "Kurtosis", "Shapiro-Wilk W", "Shapiro-Wilk p"),
Value = c(
round(skewness(data_DD_subj_level$k_trans)$Skewness, 3),
round(kurtosis(data_DD_subj_level$k_trans)$Kurtosis, 3),
round(sw_log$statistic, 3),
format.pval(sw_log$p.value, eps = .001))
) %>% kable(caption = "Normality diagnostics — log-transformed k")
```

Normality diagnostics — log-transformed k

| Statistic | Value |
| --- | --- |
| Skewness | 0.04 |
| Kurtosis | -0.237 |
| Shapiro-Wilk W | 0.994 |
| Shapiro-Wilk p | 0.65235 |

---

# DISCOUNT RATE ANALYSIS

LMMs with random intercepts per participant; `rew_max_c`
as ordered factor, first-degree polynomial contrast (see §2.4.3). Three
nested models compared via LRT.

```
m_DD_K_null <- lmer(k_trans ~ poly(rew_max_c, 1) + (1 | id),
data = data_DD_subj_level)
m_DD_K_no_int <- lmer(k_trans ~ stimulation + poly(rew_max_c, 1) + (1 | id),
data = data_DD_subj_level)
m_DD_K_int <- lmer(k_trans ~ stimulation * poly(rew_max_c, 1) + (1 | id),
data = data_DD_subj_level)

kable(as.data.frame(anova(m_DD_K_null, m_DD_K_no_int, m_DD_K_int)),
caption = "Likelihood ratio tests: model comparison",
digits = 3)
```

Likelihood ratio tests: model comparison

|  | npar | AIC | BIC | logLik | -2\*log(L) | Chisq | Df | Pr(>Chisq) |
| --- | --- | --- | --- | --- | --- | --- | --- | --- |
| m\_DD\_K\_null | 4 | 740.199 | 753.352 | -366.099 | 732.199 | NA | NA | NA |
| m\_DD\_K\_no\_int | 5 | 738.172 | 754.613 | -364.086 | 728.172 | 4.027 | 1 | 0.045 |
| m\_DD\_K\_int | 6 | 740.170 | 759.900 | -364.085 | 728.170 | 0.002 | 1 | 0.968 |

Winning model: `m_DD_K_no_int`. R² marginal/conditional
via Nakagawa & Schielzeth.

```
summary(m_DD_K_no_int)
```

```
## Linear mixed model fit by REML ['lmerMod']
## Formula: k_trans ~ stimulation + poly(rew_max_c, 1) + (1 | id)
##    Data: data_DD_subj_level
## 
## REML criterion at convergence: 727.9
## 
## Scaled residuals: 
##     Min      1Q  Median      3Q     Max 
## -3.1765 -0.5883 -0.0094  0.6127  2.3234 
## 
## Random effects:
##  Groups   Name        Variance Std.Dev.
##  id       (Intercept) 4.011    2.003   
##  Residual             1.460    1.208   
## Number of obs: 198, groups:  id, 33
## 
## Fixed effects:
##                    Estimate Std. Error t value
## (Intercept)         -2.6708     0.3692  -7.234
## stimulationsham     -0.3446     0.1717  -2.007
## poly(rew_max_c, 1) -10.8904     1.2081  -9.014
## 
## Correlation of Fixed Effects:
##             (Intr) stmltn
## stimultnshm -0.233       
## ply(rw__,1)  0.000  0.000
```

```
kable(as.data.frame(car::Anova(m_DD_K_no_int)),
caption = "Type II Wald chi-square tests — winning model",
digits = 3)
```

Type II Wald chi-square tests — winning model

|  | Chisq | Df | Pr(>Chisq) |
| --- | --- | --- | --- |
| stimulation | 4.027 | 1 | 0.045 |
| poly(rew\_max\_c, 1) | 81.260 | 1 | 0.000 |

```
sjPlot::tab_model(
m_DD_K_no_int,
string.pred = "Effect",
string.est = "β",
string.ci = "95% CI",
string.p = "p"
)
```

|  | k\_trans | | |
| --- | --- | --- | --- |
| Effect | β | 95% CI | p |
| (Intercept) | -2.67 | -3.40 – -1.94 | **<0.001** |
| stimulation [sham] | -0.34 | -0.68 – -0.01 | **0.046** |
| rew max c [ degree] | -10.89 | -13.27 – -8.51 | **<0.001** |
| Random Effects | | | |
| σ^2^ | 1.46 | | |
| τ_00_ _id_ | 4.01 | | || ICC | 0.73 | | || N _id_ | 33 | | || Observations | 198 | | |
| Marginal R^2^ / Conditional R^2^ | 0.104 / 0.761 | | |

EMMs per stimulation condition; pairwise contrast
Bonferroni-adjusted.

```
emm_options(pbkrtest.limit = 5000)
emm_stim <- emmeans(m_DD_K_no_int, ~ stimulation)

kable(as.data.frame(emm_stim),
caption = "Estimated marginal means by stimulation condition",
digits = 3)
```

Estimated marginal means by stimulation condition

| stimulation | emmean | SE | df | lower.CL | upper.CL |
| --- | --- | --- | --- | --- | --- |
| beta | -2.671 | 0.369 | 35.741 | -3.420 | -1.922 |
| sham | -3.015 | 0.369 | 35.741 | -3.764 | -2.266 |

```
kable(as.data.frame(pairs(emm_stim, adjust = "bonferroni")),
caption = "Pairwise contrast: beta vs sham",
digits = 3)
```

Pairwise contrast: beta vs sham

| contrast | estimate | SE | df | t.ratio | p.value |
| --- | --- | --- | --- | --- | --- |
| beta - sham | 0.345 | 0.172 | 163 | 2.007 | 0.046 |

Cohen’s *d* = mean difference in log(*k*) / residual
SD.

```
k_diff <- summary(contrast(emm_stim, method = "pairwise"))$estimate
s_resid <- sigma(m_DD_K_no_int)
d <- k_diff / s_resid

tibble(
`Mean difference (log k)` = round(k_diff, 3),
`Residual SD` = round(s_resid, 3),
`Cohen's d` = round(d, 2)
) %>% kable(caption = "Effect size for stimulation condition")
```

Effect size for stimulation condition

| Mean difference (log k) | Residual SD | Cohen’s d |
| --- | --- | --- |
| 0.345 | 1.208 | 0.29 |

---

# ROBUSTNESS ANALYSIS

*This section extends the discount-rate analysis above with a
covariate-adjusted robustness check of the stimulation effect on
log-transformed* k*.*

Additional variables accounted for: session order
(`session`; 2 levels: ‘1st’ vs ‘2nd’), task order
(`task`; 2 levels: MTT-first vs. DD&PM-first), and
self-reported side effects of stimulation (itching - `itch`,
pain - `pain`, burning - `burn`, heat -
`heat`, tingling - `ting`, fatigue -
`fatg`, other - `oth`, task impairment -
`impair`), phosphene perception (`phos`,
dichotomic).

## Covariate-Adjusted Discount-Rate Model

The winning model (`m_DD_K_no_int`, above) is re-estimated
with session order, task order, and self-reported side
effects/phosphenes added as covariates.

```
m_DD_K_no_int_robust <- lmer(
k_trans ~ stimulation + poly(rew_max_c, 1) + (1 | id) +
as.factor(session) + as.factor(task) + impair + itch + pain + burn + heat + ting + fatg + oth +
as.factor(phos),
data = data_DD_subj_level)

summary(m_DD_K_no_int_robust)
```

```
## Linear mixed model fit by REML ['lmerMod']
## Formula: 
## k_trans ~ stimulation + poly(rew_max_c, 1) + (1 | id) + as.factor(session) +  
##     as.factor(task) + impair + itch + pain + burn + heat + ting +  
##     fatg + oth + as.factor(phos)
##    Data: data_DD_subj_level
## 
## REML criterion at convergence: 721.5
## 
## Scaled residuals: 
##      Min       1Q   Median       3Q      Max 
## -2.99713 -0.57308  0.00986  0.62970  2.49099 
## 
## Random effects:
##  Groups   Name        Variance Std.Dev.
##  id       (Intercept) 4.031    2.008   
##  Residual             1.447    1.203   
## Number of obs: 198, groups:  id, 33
## 
## Fixed effects:
##                           Estimate Std. Error t value
## (Intercept)               -2.73160    0.46708  -5.848
## stimulationsham           -0.39395    0.22272  -1.769
## poly(rew_max_c, 1)       -10.89043    1.20282  -9.054
## as.factor(session)2nd      0.39336    0.19480   2.019
## as.factor(task)MTT_first   0.08492    0.20336   0.418
## impair                     0.42912    0.33862   1.267
## itch                      -0.43752    0.38901  -1.125
## pain                      -0.45067    0.62367  -0.723
## burn                      -0.40891    0.40179  -1.018
## heat                       0.08501    0.45832   0.185
## ting                       0.06461    0.44907   0.144
## fatg                      -0.16682    0.32038  -0.521
## oth                       -0.36568    0.28705  -1.274
## as.factor(phos)1           0.51690    0.59002   0.876
```

```
kable(as.data.frame(car::Anova(m_DD_K_no_int_robust)),
caption = "Type II Wald chi-square tests — covariate-adjusted model", digits = 3)
```

Type II Wald chi-square tests — covariate-adjusted
model

|  | Chisq | Df | Pr(>Chisq) |
| --- | --- | --- | --- |
| stimulation | 3.129 | 1 | 0.077 |
| poly(rew\_max\_c, 1) | 81.977 | 1 | 0.000 |
| as.factor(session) | 4.078 | 1 | 0.043 |
| as.factor(task) | 0.174 | 1 | 0.676 |
| impair | 1.606 | 1 | 0.205 |
| itch | 1.265 | 1 | 0.261 |
| pain | 0.522 | 1 | 0.470 |
| burn | 1.036 | 1 | 0.309 |
| heat | 0.034 | 1 | 0.853 |
| ting | 0.021 | 1 | 0.886 |
| fatg | 0.271 | 1 | 0.603 |
| oth | 1.623 | 1 | 0.203 |
| as.factor(phos) | 0.768 | 1 | 0.381 |

```
sjPlot::tab_model(
m_DD_K_no_int_robust,
string.pred = "Effect", string.est = "β", string.ci = "95% CI", string.p = "p")
```

|  | k\_trans | | |
| --- | --- | --- | --- |
| Effect | β | 95% CI | p |
| (Intercept) | -2.73 | -3.65 – -1.81 | **<0.001** |
| stimulation [sham] | -0.39 | -0.83 – 0.05 | 0.079 |
| rew max c [ degree] | -10.89 | -13.26 – -8.52 | **<0.001** |
| session [2nd] | 0.39 | 0.01 – 0.78 | **0.045** |
| task [MTT\_first] | 0.08 | -0.32 – 0.49 | 0.677 |
| impair | 0.43 | -0.24 – 1.10 | 0.207 |
| itch | -0.44 | -1.21 – 0.33 | 0.262 |
| pain | -0.45 | -1.68 – 0.78 | 0.471 |
| burn | -0.41 | -1.20 – 0.38 | 0.310 |
| heat | 0.09 | -0.82 – 0.99 | 0.853 |
| ting | 0.06 | -0.82 – 0.95 | 0.886 |
| fatg | -0.17 | -0.80 – 0.47 | 0.603 |
| oth | -0.37 | -0.93 – 0.20 | 0.204 |
| phos [1] | 0.52 | -0.65 – 1.68 | 0.382 |
| Random Effects | | | |
| σ^2^ | 1.45 | | |
| τ_00_ _id_ | 4.03 | | || ICC | 0.74 | | || N _id_ | 33 | | || Observations | 198 | | |
| Marginal R^2^ / Conditional R^2^ | 0.126 / 0.769 | | |

```
emm_options(pbkrtest.limit = 5000)
emm_stim_robust <- emmeans(m_DD_K_no_int_robust, ~ stimulation)

kable(as.data.frame(emm_stim_robust),
caption = "EMMs by stimulation condition, covariate-adjusted model", digits = 3)
```

EMMs by stimulation condition, covariate-adjusted
model

| stimulation | emmean | SE | df | lower.CL | upper.CL |
| --- | --- | --- | --- | --- | --- |
| beta | -2.698 | 0.537 | 98.968 | -3.765 | -1.632 |
| sham | -3.092 | 0.484 | 78.266 | -4.055 | -2.129 |

```
kable(as.data.frame(pairs(emm_stim_robust, adjust = "bonferroni")),
caption = "Pairwise contrast: beta vs sham, covariate-adjusted model", digits = 3)
```

Pairwise contrast: beta vs sham, covariate-adjusted
model

| contrast | estimate | SE | df | t.ratio | p.value |
| --- | --- | --- | --- | --- | --- |
| beta - sham | 0.394 | 0.224 | 168.453 | 1.762 | 0.08 |

```
k_diff_robust <- summary(contrast(emm_stim_robust, method = "pairwise"))$estimate
s_resid_robust <- sigma(m_DD_K_no_int_robust)
d_robust <- k_diff_robust / s_resid_robust

tibble(
`Mean difference (log k)` = round(k_diff_robust, 3),
`Residual SD` = round(s_resid_robust, 3),
`Cohen's d` = round(d_robust, 2)
) %>% kable(caption = "Effect size for stimulation condition, covariate-adjusted model")
```

Effect size for stimulation condition, covariate-adjusted
model

| Mean difference (log k) | Residual SD | Cohen’s d |
| --- | --- | --- |
| 0.394 | 1.203 | 0.33 |
