# Supplementary material for: Beta-Band Stimulation of Dorsolateral Prefrontal Cortex Through tACS Alters Mental Time Travel and Prospective Memory, but Not Delay Discounting
Source: Brain Sci. 2026 Aug 22;16(9):899. doi: 10.3390/brainsci16090899 (PMC13604682; doi:10.3390/brainsci16090899)
Supplement: Supplementary file 1 [file brainsci-16-00899-s001.zip › S5 Mental Time Travel (code markdown).html]

tACS\_future\_Mental\_Time\_Travel


# tACS\_future\_Mental\_Time\_Travel

#### Authors are masked for peer review purposes

# PRELIMINARY

```
set.seed(12313423)
```

`data_MTT`: 5,184 obs × 9 variables — `id`
(chr, 36 participants), `RT` (ms), `correct`
(0/1), `stim` (fct: beta/sham), `type` (fct:
impersonal/personal), `ref_cat` (fct: 2014/2024/2034),
`dist_ref` (years, signed), `ar_gen` (continuous
arousal rating), `guess` (fct: 0/1).

---

# RT ANALYSIS

## Data Preparation

Correct trials only; anticipatory responses excluded (RT < 500 ms,
N = 3). Omitted responses excluded as incorrect by design.

```
data_MTT_RT <- data_MTT %>%
  filter(correct == TRUE, RT > 500)
```

## Normality Assessment — RT

Inverse transformation (1000/RT) selected over log (see §2.4.2).
Outliers removed via Tukey method (2×IQR). Residual Shapiro-Wilk
significance attributable to large N.

```
tibble(
  Transform = c("Raw RT", "1000/RT"),
  Skewness = c(
    round(skewness(data_MTT_RT$RT)$Skewness, 3),
    round(skewness(1000/data_MTT_RT$RT)$Skewness, 3)),
  Kurtosis = c(
    round(kurtosis(data_MTT_RT$RT)$Kurtosis, 3),
    round(kurtosis(1000/data_MTT_RT$RT)$Kurtosis, 3)),
  `Shapiro-Wilk W` = c(
    round(shapiro.test(data_MTT_RT$RT)$statistic, 3),
    round(shapiro.test(1000/data_MTT_RT$RT)$statistic, 3)),
  `Shapiro-Wilk p` = c(
    format.pval(shapiro.test(data_MTT_RT$RT)$p.value, eps = .001),
    format.pval(shapiro.test(1000/data_MTT_RT$RT)$p.value, eps = .001))
) %>% kable(caption = "Normality diagnostics — RT transformations")
```

Normality diagnostics — RT transformations

| Transform | Skewness | Kurtosis | Shapiro-Wilk W | Shapiro-Wilk p |
| --- | --- | --- | --- | --- |
| Raw RT | 1.849 | 4.376 | 0.837 | < 0.001 |
| 1000/RT | 0.135 | -0.397 | 0.995 | < 0.001 |

```
data_MTT_RT <- data_MTT_RT %>%
  mutate(RT = 1000/RT) %>%
  filter(RT > median(RT) - 2*IQR(RT),
         RT < median(RT) + 2*IQR(RT))

tibble(
  Statistic = c("Skewness", "Kurtosis", "Shapiro-Wilk W", "Shapiro-Wilk p"),
  Value = c(
    round(skewness(data_MTT_RT$RT)$Skewness, 3),
    round(kurtosis(data_MTT_RT$RT)$Kurtosis, 3),
    round(shapiro.test(data_MTT_RT$RT)$statistic, 3),
    format.pval(shapiro.test(data_MTT_RT$RT)$p.value, eps = .001))
) %>% kable(caption = "Normality diagnostics — inverse RT after outlier removal")
```

Normality diagnostics — inverse RT after outlier
removal

| Statistic | Value |
| --- | --- |
| Skewness | 0.103 |
| Kurtosis | -0.483 |
| Shapiro-Wilk W | 0.994 |
| Shapiro-Wilk p | < 0.001 |

## Normality Assessment — RT Covariates

`d_ref_ab` = log(|dist\_ref| + 1); constant added to avoid
log(0) for personal events at zero distance (see §2.4.2).

```
data_MTT_RT <- data_MTT_RT %>%
  mutate(d_ref_ab = log(abs(dist_ref) + 1),
         guess = ifelse(guess == 1, "beta", "sham"))

tibble(
  Variable = c("dist_ref (raw)", "d_ref_ab (log-transformed)", "ar_gen"),
  Skewness = c(
    round(skewness(abs(data_MTT_RT$dist_ref))$Skewness, 3),
    round(skewness(data_MTT_RT$d_ref_ab)$Skewness, 3),
    round(skewness(data_MTT_RT$ar_gen)$Skewness, 3)),
  Kurtosis = c(
    round(kurtosis(abs(data_MTT_RT$dist_ref))$Kurtosis, 3),
    round(kurtosis(data_MTT_RT$d_ref_ab)$Kurtosis, 3),
    round(kurtosis(data_MTT_RT$ar_gen)$Kurtosis, 3)),
  `Shapiro-Wilk W` = c(
    round(shapiro.test(abs(data_MTT_RT$dist_ref))$statistic, 3),
    round(shapiro.test(data_MTT_RT$d_ref_ab)$statistic, 3),
    round(shapiro.test(data_MTT_RT$ar_gen)$statistic, 3))
) %>% kable(caption = "Normality diagnostics — RT covariates")
```

Normality diagnostics — RT covariates

| Variable | Skewness | Kurtosis | Shapiro-Wilk W |
| --- | --- | --- | --- |
| dist\_ref (raw) | 1.589 | 3.703 | 0.870 |
| d\_ref\_ab (log-transformed) | -0.522 | 0.016 | 0.964 |
| ar\_gen | 0.022 | -0.805 | 0.969 |

## Model Comparison

LMM with random intercepts and slopes for event type per participant.
Three nested models compared via LRT (see §2.4.2).

```
m_MTT_RT_null <- lmer(RT ~ d_ref_ab + ref_cat + ar_gen + guess + (1 + type | id),
                       data = data_MTT_RT)
m_MTT_RT_no_int <- lmer(RT ~ stim + d_ref_ab + ref_cat + ar_gen + guess + (1 + type | id),
                         data = data_MTT_RT)
m_MTT_RT_int <- lmer(RT ~ stim*ref_cat + d_ref_ab + ar_gen + guess + (1 + type | id),
                      data = data_MTT_RT)

kable(as.data.frame(anova(m_MTT_RT_null, m_MTT_RT_no_int, m_MTT_RT_int)),
      caption = "Likelihood ratio tests — RT models", digits = 3)
```

Likelihood ratio tests — RT models

|  | npar | AIC | BIC | logLik | -2\*log(L) | Chisq | Df | Pr(>Chisq) |
| --- | --- | --- | --- | --- | --- | --- | --- | --- |
| m\_MTT\_RT\_null | 10 | -1029.284 | -964.695 | 524.642 | -1049.284 | NA | NA | NA |
| m\_MTT\_RT\_no\_int | 11 | -1030.502 | -959.454 | 526.251 | -1052.502 | 3.218 | 1 | 0.073 |
| m\_MTT\_RT\_int | 13 | -1027.593 | -943.627 | 526.797 | -1053.593 | 1.091 | 2 | 0.579 |

Null model retained. R² marginal/conditional via Nakagawa &
Schielzeth.

```
summary(m_MTT_RT_null)
```

```
## Linear mixed model fit by REML ['lmerMod']
## Formula: RT ~ d_ref_ab + ref_cat + ar_gen + guess + (1 + type | id)
##    Data: data_MTT_RT
## 
## REML criterion at convergence: -1001.3
## 
## Scaled residuals: 
##     Min      1Q  Median      3Q     Max 
## -3.1085 -0.6848  0.0129  0.6723  3.6631 
## 
## Random effects:
##  Groups   Name         Variance Std.Dev. Corr 
##  id       (Intercept)  0.012004 0.10956       
##           typepersonal 0.002194 0.04684  -0.07
##  Residual              0.045341 0.21294       
## Number of obs: 4717, groups:  id, 36
## 
## Fixed effects:
##              Estimate Std. Error t value
## (Intercept)  0.636672   0.024192  26.318
## d_ref_ab     0.063467   0.004175  15.200
## ref_cat2024  0.016029   0.007742   2.070
## ref_cat2034 -0.038640   0.007992  -4.835
## ar_gen      -0.005959   0.005108  -1.167
## guesssham   -0.027181   0.007990  -3.402
## 
## Correlation of Fixed Effects:
##             (Intr) d_rf_b r_2024 r_2034 ar_gen
## d_ref_ab    -0.400                            
## ref_cat2024 -0.015 -0.132                     
## ref_cat2034  0.036 -0.219  0.525              
## ar_gen      -0.471  0.040 -0.186 -0.215       
## guesssham   -0.110 -0.003 -0.004 -0.006 -0.004
```

```
kable(as.data.frame(car::Anova(m_MTT_RT_null)),
      caption = "Type II Wald chi-square tests — RT null model", digits = 3)
```

Type II Wald chi-square tests — RT null model

|  | Chisq | Df | Pr(>Chisq) |
| --- | --- | --- | --- |
| d\_ref\_ab | 231.053 | 1 | 0.000 |
| ref\_cat | 52.650 | 2 | 0.000 |
| ar\_gen | 1.361 | 1 | 0.243 |
| guess | 11.574 | 1 | 0.001 |

```
sjPlot::tab_model(
  m_MTT_RT_null,
  string.pred = "Effect", string.est = "β", string.ci = "95% CI", string.p = "p")
```

|  | RT | | |
| --- | --- | --- | --- |
| Effect | β | 95% CI | p |
| (Intercept) | 0.64 | 0.59 – 0.68 | **<0.001** |
| d ref ab | 0.06 | 0.06 – 0.07 | **<0.001** |
| ref cat [2024] | 0.02 | 0.00 – 0.03 | **0.038** |
| ref cat [2034] | -0.04 | -0.05 – -0.02 | **<0.001** |
| ar gen | -0.01 | -0.02 – 0.00 | 0.243 |
| guess [sham] | -0.03 | -0.04 – -0.01 | **0.001** |
| Random Effects | | | |
| σ^2^ | 0.05 | | |
| τ_00_ _id_ | 0.01 | | || τ_11_ _id.typepersonal_ | 0.00 | | || ρ_01_ _id_ | -0.07 | | || ICC | 0.21 | | || N _id_ | 36 | | || Observations | 4717 | | |
| Marginal R^2^ / Conditional R^2^ | 0.046 / 0.246 | | |

Significant `guess` effect: faster RTs when participants
believed they received active stimulation.

```
emm_options(pbkrtest.limit = 5000)
kable(as.data.frame(pairs(emmeans(m_MTT_RT_null, ~ guess))),
      caption = "Pairwise contrast: guessed beta vs guessed sham (RT)", digits = 3)
```

Pairwise contrast: guessed beta vs guessed sham (RT)

| contrast | estimate | SE | df | t.ratio | p.value |
| --- | --- | --- | --- | --- | --- |
| beta - sham | 0.027 | 0.008 | 4641.639 | 3.398 | 0.001 |

---

# ACCURACY ANALYSIS

## Data Preparation

All trials retained; omitted responses coded as incorrect. Low error
rate (0.83%) motivated simplified random effects: random intercepts for
event type nested within participant.

```
data_MTT_ACC <- data_MTT %>%
  mutate(correct = ifelse(is.na(correct), 0, correct),
         d_ref_ab = log(abs(dist_ref) + 1))
```

`data_MTT_ACC`: 5,184 obs × 10 variables — same structure
as `data_MTT` plus `d_ref_ab`.

## Normality Assessment — Accuracy Covariates

```
tibble(
  Variable = c("dist_ref (raw)", "d_ref_ab (log-transformed)", "ar_gen"),
  Skewness = c(
    round(skewness(data_MTT_ACC$dist_ref)$Skewness, 3),
    round(skewness(data_MTT_ACC$d_ref_ab)$Skewness, 3),
    round(skewness(data_MTT_ACC$ar_gen)$Skewness, 3)),
  Kurtosis = c(
    round(kurtosis(data_MTT_ACC$dist_ref)$Kurtosis, 3),
    round(kurtosis(data_MTT_ACC$d_ref_ab)$Kurtosis, 3),
    round(kurtosis(data_MTT_ACC$ar_gen)$Kurtosis, 3)),
  `Shapiro-Wilk W` = c(
    round(shapiro.test(sample(data_MTT_ACC$dist_ref, 5000))$statistic, 3),
    round(shapiro.test(sample(data_MTT_ACC$d_ref_ab, 5000))$statistic, 3),
    round(shapiro.test(sample(data_MTT_ACC$ar_gen, 5000))$statistic, 3))
) %>% kable(caption = "Normality diagnostics — accuracy covariates")
```

Normality diagnostics — accuracy covariates

| Variable | Skewness | Kurtosis | Shapiro-Wilk W |
| --- | --- | --- | --- |
| dist\_ref (raw) | 0.854 | 0.584 | 0.943 |
| d\_ref\_ab (log-transformed) | -0.472 | -0.117 | 0.964 |
| ar\_gen | 0.033 | -0.813 | 0.969 |

## Model Comparison

GLMM (binomial, logit link); same fixed effects as RT analysis. Three
nested models compared via LRT (see §2.4.2).

```
m_MTT_ACC_null <- glmer(
  correct ~ d_ref_ab + ref_cat + ar_gen + guess + (1 | id/type),
  family = binomial, data = data_MTT_ACC,
  control = glmerControl(optimizer = "bobyqa"))

m_MTT_ACC_no_int <- glmer(
  correct ~ stim + d_ref_ab + ref_cat + ar_gen + guess + (1 | id/type),
  family = binomial, data = data_MTT_ACC,
  control = glmerControl(optimizer = "bobyqa"))

m_MTT_ACC_int <- glmer(
  correct ~ stim*ref_cat + d_ref_ab + ar_gen + guess + (1 | id/type),
  family = binomial, data = data_MTT_ACC,
  control = glmerControl(optimizer = "bobyqa"))

kable(as.data.frame(anova(m_MTT_ACC_null, m_MTT_ACC_no_int, m_MTT_ACC_int)),
      caption = "Likelihood ratio tests — accuracy models", digits = 3)
```

Likelihood ratio tests — accuracy models

|  | npar | AIC | BIC | logLik | -2\*log(L) | Chisq | Df | Pr(>Chisq) |
| --- | --- | --- | --- | --- | --- | --- | --- | --- |
| m\_MTT\_ACC\_null | 8 | 2748.175 | 2800.602 | -1366.088 | 2732.175 | NA | NA | NA |
| m\_MTT\_ACC\_no\_int | 9 | 2730.003 | 2788.983 | -1356.002 | 2712.003 | 20.172 | 1 | 0.000 |
| m\_MTT\_ACC\_int | 11 | 2725.118 | 2797.205 | -1351.559 | 2703.118 | 8.885 | 2 | 0.012 |

Interaction model retained. R² marginal/conditional via Nakagawa
& Schielzeth.

```
summary(m_MTT_ACC_int)
```

```
## Generalized linear mixed model fit by maximum likelihood (Laplace
##   Approximation) [glmerMod]
##  Family: binomial  ( logit )
## Formula: correct ~ stim * ref_cat + d_ref_ab + ar_gen + guess + (1 | id/type)
##    Data: data_MTT_ACC
## Control: glmerControl(optimizer = "bobyqa")
## 
##       AIC       BIC    logLik -2*log(L)  df.resid 
##    2725.1    2797.2   -1351.6    2703.1      5173 
## 
## Scaled residuals: 
##     Min      1Q  Median      3Q     Max 
## -8.6584  0.1589  0.2239  0.3114  1.8710 
## 
## Random effects:
##  Groups  Name        Variance Std.Dev.
##  type:id (Intercept) 0.3107   0.5574  
##  id      (Intercept) 0.4838   0.6955  
## Number of obs: 5184, groups:  type:id, 72; id, 36
## 
## Fixed effects:
##                      Estimate Std. Error z value Pr(>|z|)    
## (Intercept)           1.77680    0.35185   5.050 4.42e-07 ***
## stimsham             -0.72717    0.21152  -3.438 0.000586 ***
## ref_cat2024          -0.20908    0.22123  -0.945 0.344627    
## ref_cat2034          -1.19162    0.19703  -6.048 1.47e-09 ***
## d_ref_ab              0.61270    0.06474   9.464  < 2e-16 ***
## ar_gen                0.21645    0.09219   2.348 0.018881 *  
## guess1               -0.36012    0.16498  -2.183 0.029046 *  
## stimsham:ref_cat2024 -0.18890    0.27912  -0.677 0.498562    
## stimsham:ref_cat2034  0.52638    0.25600   2.056 0.039764 *  
## ---
## Signif. codes:  0 '***' 0.001 '**' 0.01 '*' 0.05 '.' 0.1 ' ' 1
## 
## Correlation of Fixed Effects:
##             (Intr) stmshm r_2024 r_2034 d_rf_b ar_gen guess1 s:_202
## stimsham    -0.445                                                 
## ref_cat2024 -0.217  0.519                                          
## ref_cat2034 -0.212  0.583  0.582                                   
## d_ref_ab    -0.372 -0.019 -0.052 -0.130                            
## ar_gen      -0.600 -0.010 -0.132 -0.166  0.047                     
## guess1      -0.402  0.318  0.005 -0.002 -0.016 -0.006              
## stmsh:_2024  0.244 -0.679 -0.777 -0.438 -0.003  0.003  0.000       
## stmsh:_2034  0.260 -0.739 -0.427 -0.736  0.010  0.006  0.008  0.561
```

```
kable(as.data.frame(car::Anova(m_MTT_ACC_int, type = "III")),
      caption = "Type III Wald chi-square tests — accuracy interaction model", digits = 3)
```

Type III Wald chi-square tests — accuracy interaction
model

|  | Chisq | Df | Pr(>Chisq) |
| --- | --- | --- | --- |
| (Intercept) | 25.501 | 1 | 0.000 |
| stim | 11.819 | 1 | 0.001 |
| ref\_cat | 46.628 | 2 | 0.000 |
| d\_ref\_ab | 89.575 | 1 | 0.000 |
| ar\_gen | 5.513 | 1 | 0.019 |
| guess | 4.765 | 1 | 0.029 |
| stim:ref\_cat | 9.115 | 2 | 0.010 |

```
sjPlot::tab_model(
  m_MTT_ACC_int,
  string.pred = "Effect", string.est = "β", string.ci = "95% CI", string.p = "p")
```

|  | correct | | |
| --- | --- | --- | --- |
| Effect | β | 95% CI | p |
| (Intercept) | 5.91 | 2.97 – 11.78 | **<0.001** |
| stim [sham] | 0.48 | 0.32 – 0.73 | **0.001** |
| ref cat [2024] | 0.81 | 0.53 – 1.25 | 0.345 |
| ref cat [2034] | 0.30 | 0.21 – 0.45 | **<0.001** |
| d ref ab | 1.85 | 1.63 – 2.10 | **<0.001** |
| ar gen | 1.24 | 1.04 – 1.49 | **0.019** |
| guess [1] | 0.70 | 0.50 – 0.96 | **0.029** |
| stim [sham] × ref cat [2024] | 0.83 | 0.48 – 1.43 | 0.499 |
| stim [sham] × ref cat [2034] | 1.69 | 1.02 – 2.80 | **0.040** |
| Random Effects | | | |
| σ^2^ | 3.29 | | |
| τ_00_ _type:id_ | 0.31 | | || τ_00_ _id_ | 0.48 | | || ICC | 0.19 | | || N _type_ | 2 | | || N _id_ | 36 | | || Observations | 5184 | | |
| Marginal R^2^ / Conditional R^2^ | 0.091 / 0.268 | | |

EMMs and pairwise contrasts (beta vs sham) per reference point;
Bonferroni-adjusted.

```
emm_options(pbkrtest.limit = 5000)
emm_stim <- emmeans(m_MTT_ACC_int, ~ stim | ref_cat)

kable(as.data.frame(emm_stim),
      caption = "Estimated marginal means by stimulation × reference point (log-odds scale)",
      digits = 3)
```

Estimated marginal means by stimulation × reference point
(log-odds scale)

| stim | ref\_cat | emmean | SE | df | asymp.LCL | asymp.UCL |
| --- | --- | --- | --- | --- | --- | --- |
| beta | 2014 | 3.624 | 0.223 | Inf | 3.187 | 4.061 |
| sham | 2014 | 2.897 | 0.192 | Inf | 2.520 | 3.274 |
| beta | 2024 | 3.415 | 0.218 | Inf | 2.987 | 3.842 |
| sham | 2024 | 2.499 | 0.182 | Inf | 2.141 | 2.856 |
| beta | 2034 | 2.432 | 0.189 | Inf | 2.062 | 2.802 |
| sham | 2034 | 2.231 | 0.177 | Inf | 1.884 | 2.579 |

```
kable(as.data.frame(pairs(emm_stim, adjust = "bonferroni")),
      caption = "Pairwise contrasts: beta vs sham per reference point (log-OR scale)",
      digits = 3)
```

Pairwise contrasts: beta vs sham per reference point (log-OR
scale)

| contrast | ref\_cat | estimate | SE | df | z.ratio | p.value |
| --- | --- | --- | --- | --- | --- | --- |
| beta - sham | 2014 | 0.727 | 0.212 | Inf | 3.438 | 0.001 |
| beta - sham | 2024 | 0.916 | 0.206 | Inf | 4.446 | 0.000 |
| beta - sham | 2034 | 0.201 | 0.174 | Inf | 1.155 | 0.248 |

---

# ROBUSTNESS ANALYSIS

*This section extends the accuracy analysis above with a
covariate-adjusted robustness check of the stimulation × reference-point
interaction.*

Additional variables accounted for: session order
(`session`; 2 levels: ‘1st’ vs ‘2nd’), task order
(`order_task`; 2 levels: MTT-first vs. DD&PM-first),
reference-point order (`order_ref`; 6 levels: permutation
order of PAST/PRESENT/FUTURE reference points within MTT), and
self-reported side effects of stimulation (itching - `itch`,
pain - `pain`, burning - `burn`, tingling -
`ting`, fatigue - `fatg`, other -
`oth`, phosphene perception (`phos`,
dichotomic).

## Covariate-Adjusted Accuracy Model

The winning interaction model (`m_MTT_ACC_int`, above) is
re-estimated with session order, task order, reference-point order, and
self-reported side effects/phosphenes added as covariates.

```
m_MTT_ACC_int_robust <- glmer(
  correct ~ stim*ref_cat + d_ref_ab + ar_gen + as.factor(guess.x) + (1 | id/type) +
    as.factor(task) + as.factor(session) + as.factor(phos) + as.factor(order_ref) +
    itch + pain + burn + ting + fatg + oth,
  family = binomial, data = data_MTT_ACC,
  control = glmerControl(optimizer = "bobyqa"))

summary(m_MTT_ACC_int_robust)
```

```
## Generalized linear mixed model fit by maximum likelihood (Laplace
##   Approximation) [glmerMod]
##  Family: binomial  ( logit )
## Formula: correct ~ stim * ref_cat + d_ref_ab + ar_gen + as.factor(guess.x) +  
##     (1 | id/type) + as.factor(task) + as.factor(session) + as.factor(phos) +  
##     as.factor(order_ref) + itch + pain + burn + ting + fatg +      oth
##    Data: data_MTT_ACC
## Control: glmerControl(optimizer = "bobyqa")
## 
##       AIC       BIC    logLik -2*log(L)  df.resid 
##    2680.6    2844.4   -1315.3    2630.6      5159 
## 
## Scaled residuals: 
##     Min      1Q  Median      3Q     Max 
## -8.8563  0.1542  0.2171  0.3051  2.3068 
## 
## Random effects:
##  Groups  Name        Variance Std.Dev.
##  type:id (Intercept) 0.3158   0.5619  
##  id      (Intercept) 0.3640   0.6033  
## Number of obs: 5184, groups:  type:id, 72; id, 36
## 
## Fixed effects:
##                          Estimate Std. Error z value Pr(>|z|)    
## (Intercept)               1.53015    0.46211   3.311 0.000929 ***
## stimsham                 -0.55930    0.22083  -2.533 0.011320 *  
## ref_cat2024              -0.20930    0.21784  -0.961 0.336666    
## ref_cat2034              -1.16412    0.19398  -6.001 1.96e-09 ***
## d_ref_ab                  0.63079    0.06580   9.587  < 2e-16 ***
## ar_gen                    0.22232    0.09362   2.375 0.017558 *  
## as.factor(guess.x)1       0.43777    0.21874   2.001 0.045361 *  
## as.factor(task)MTT_first  0.29350    0.12165   2.413 0.015837 *  
## as.factor(session)2      -0.31647    0.12598  -2.512 0.012005 *  
## as.factor(phos)1         -0.06983    0.33261  -0.210 0.833710    
## as.factor(order_ref)132  -0.15584    0.45371  -0.343 0.731242    
## as.factor(order_ref)213   0.31436    0.44379   0.708 0.478723    
## as.factor(order_ref)231   0.01081    0.45486   0.024 0.981042    
## as.factor(order_ref)312   0.23829    0.48247   0.494 0.621377    
## as.factor(order_ref)321  -0.58172    0.47859  -1.215 0.224180    
## itch                     -0.74635    0.18854  -3.958 7.54e-05 ***
## pain                      0.71481    0.34781   2.055 0.039859 *  
## burn                     -0.57622    0.18640  -3.091 0.001993 ** 
## ting                      0.08095    0.18625   0.435 0.663814    
## fatg                      0.22322    0.18115   1.232 0.217853    
## oth                      -0.24118    0.15809  -1.526 0.127123    
## stimsham:ref_cat2024     -0.21644    0.28145  -0.769 0.441868    
## stimsham:ref_cat2034      0.45182    0.25899   1.745 0.081064 .  
## ---
## Signif. codes:  0 '***' 0.001 '**' 0.01 '*' 0.05 '.' 0.1 ' ' 1
```

```
kable(as.data.frame(car::Anova(m_MTT_ACC_int_robust, type = "III")),
      caption = "Type III Wald chi-square tests — covariate-adjusted accuracy model", digits = 3)
```

Type III Wald chi-square tests — covariate-adjusted accuracy
model

|  | Chisq | Df | Pr(>Chisq) |
| --- | --- | --- | --- |
| (Intercept) | 10.964 | 1 | 0.001 |
| stim | 6.414 | 1 | 0.011 |
| ref\_cat | 45.776 | 2 | 0.000 |
| d\_ref\_ab | 91.914 | 1 | 0.000 |
| ar\_gen | 5.640 | 1 | 0.018 |
| as.factor(guess.x) | 4.005 | 1 | 0.045 |
| as.factor(task) | 5.821 | 1 | 0.016 |
| as.factor(session) | 6.310 | 1 | 0.012 |
| as.factor(phos) | 0.044 | 1 | 0.834 |
| as.factor(order\_ref) | 4.277 | 5 | 0.510 |
| itch | 15.670 | 1 | 0.000 |
| pain | 4.224 | 1 | 0.040 |
| burn | 9.556 | 1 | 0.002 |
| ting | 0.189 | 1 | 0.664 |
| fatg | 1.518 | 1 | 0.218 |
| oth | 2.327 | 1 | 0.127 |
| stim:ref\_cat | 7.514 | 2 | 0.023 |

```
sjPlot::tab_model(
  m_MTT_ACC_int_robust,
  string.pred = "Effect", string.est = "β", string.ci = "95% CI", string.p = "p")
```

|  | correct | | |
| --- | --- | --- | --- |
| Effect | β | 95% CI | p |
| (Intercept) | 4.62 | 1.87 – 11.43 | **0.001** |
| stim [sham] | 0.57 | 0.37 – 0.88 | **0.011** |
| ref cat [2024] | 0.81 | 0.53 – 1.24 | 0.337 |
| ref cat [2034] | 0.31 | 0.21 – 0.46 | **<0.001** |
| d ref ab | 1.88 | 1.65 – 2.14 | **<0.001** |
| ar gen | 1.25 | 1.04 – 1.50 | **0.018** |
| guess x [1] | 1.55 | 1.01 – 2.38 | **0.045** |
| task [MTT\_first] | 1.34 | 1.06 – 1.70 | **0.016** |
| session [2] | 0.73 | 0.57 – 0.93 | **0.012** |
| phos [1] | 0.93 | 0.49 – 1.79 | 0.834 |
| order ref [132] | 0.86 | 0.35 – 2.08 | 0.731 |
| order ref [213] | 1.37 | 0.57 – 3.27 | 0.479 |
| order ref [231] | 1.01 | 0.41 – 2.47 | 0.981 |
| order ref [312] | 1.27 | 0.49 – 3.27 | 0.621 |
| order ref [321] | 0.56 | 0.22 – 1.43 | 0.224 |
| itch | 0.47 | 0.33 – 0.69 | **<0.001** |
| pain | 2.04 | 1.03 – 4.04 | **0.040** |
| burn | 0.56 | 0.39 – 0.81 | **0.002** |
| ting | 1.08 | 0.75 – 1.56 | 0.664 |
| fatg | 1.25 | 0.88 – 1.78 | 0.218 |
| oth | 0.79 | 0.58 – 1.07 | 0.127 |
| stim [sham] × ref cat [2024] | 0.81 | 0.46 – 1.40 | 0.442 |
| stim [sham] × ref cat [2034] | 1.57 | 0.95 – 2.61 | 0.081 |
| Random Effects | | | |
| σ^2^ | 3.29 | | |
| τ_00_ _type:id_ | 0.32 | | || τ_00_ _id_ | 0.36 | | || ICC | 0.17 | | || N _type_ | 2 | | || N _id_ | 36 | | || Observations | 5184 | | |
| Marginal R^2^ / Conditional R^2^ | 0.158 / 0.302 | | |

```
emm_options(pbkrtest.limit = 5000)
emm_stim_robust <- emmeans(m_MTT_ACC_int_robust, ~ stim | ref_cat)
emm_stim_robust_resp <- emmeans(m_MTT_ACC_int_robust, ~ stim | ref_cat, type = "response")

kable(as.data.frame(emm_stim_robust),
      caption = "EMMs — stimulation × reference point, covariate-adjusted model (log-odds scale)",
      digits = 3)
```

EMMs — stimulation × reference point, covariate-adjusted model
(log-odds scale)

| stim | ref\_cat | emmean | SE | df | asymp.LCL | asymp.UCL |
| --- | --- | --- | --- | --- | --- | --- |
| beta | 2014 | 3.740 | 0.302 | Inf | 3.148 | 4.332 |
| sham | 2014 | 3.181 | 0.259 | Inf | 2.674 | 3.688 |
| beta | 2024 | 3.531 | 0.299 | Inf | 2.945 | 4.117 |
| sham | 2024 | 2.755 | 0.250 | Inf | 2.265 | 3.245 |
| beta | 2034 | 2.576 | 0.279 | Inf | 2.030 | 3.122 |
| sham | 2034 | 2.469 | 0.245 | Inf | 1.988 | 2.949 |

```
kable(as.data.frame(pairs(emm_stim_robust, adjust = "bonferroni")),
      caption = "Pairwise contrasts: beta vs sham per reference point, covariate-adjusted (log-OR scale)",
      digits = 3)
```

Pairwise contrasts: beta vs sham per reference point,
covariate-adjusted (log-OR scale)

| contrast | ref\_cat | estimate | SE | df | z.ratio | p.value |
| --- | --- | --- | --- | --- | --- | --- |
| beta - sham | 2014 | 0.559 | 0.221 | Inf | 2.533 | 0.011 |
| beta - sham | 2024 | 0.776 | 0.214 | Inf | 3.617 | 0.000 |
| beta - sham | 2034 | 0.107 | 0.184 | Inf | 0.585 | 0.559 |

```
kable(as.data.frame(emm_stim_robust_resp),
      caption = "EMMs — stimulation × reference point, covariate-adjusted model (probability scale)",
      digits = 3)
```

EMMs — stimulation × reference point, covariate-adjusted model
(probability scale)

| stim | ref\_cat | prob | SE | df | asymp.LCL | asymp.UCL |
| --- | --- | --- | --- | --- | --- | --- |
| beta | 2014 | 0.977 | 0.007 | Inf | 0.959 | 0.987 |
| sham | 2014 | 0.960 | 0.010 | Inf | 0.935 | 0.976 |
| beta | 2024 | 0.972 | 0.008 | Inf | 0.950 | 0.984 |
| sham | 2024 | 0.940 | 0.014 | Inf | 0.906 | 0.962 |
| beta | 2034 | 0.929 | 0.018 | Inf | 0.884 | 0.958 |
| sham | 2034 | 0.922 | 0.018 | Inf | 0.880 | 0.950 |
