# Supplementary material for: Beta-Band Stimulation of Dorsolateral Prefrontal Cortex Through tACS Alters Mental Time Travel and Prospective Memory, but Not Delay Discounting
Source: Brain Sci. 2026 Aug 22;16(9):899. doi: 10.3390/brainsci16090899 (PMC13604682; doi:10.3390/brainsci16090899)
Supplement: Supplementary file 1 [file brainsci-16-00899-s001.zip › S6 Prospective Memory (code markdown).html]

BP\_tACS\_Markdown\_PM\_robustness.knit


s— title: “Cortex\_tACS\_Markdown\_Prospective\_Memory” author: “Masked”
date: “2026-05-17” output: html\_document —

`data_PM`: wide-format PM dataset — id (chr), stimulation
(chr: beta/sham), guess (int: 0/1), TIME\_CHECK (min, clock-check timing
within cycle), TIME\_PRESS (min, button-press timing), PROG\_CHECK,
PROG\_PRESS (progressive cycle counters).

---

## OUTLIER REMOVAL

### TIME\_PRESS distribution — raw

IQR/Range = .054: extreme outliers present (task neglect or
misunderstanding). Tukey **1.5×IQR** criterion applied to
TIME\_PRESS; one participant excluded due to absence of valid trials in
one condition after removal.

```
data_PM %>%
filter(!is.na(TIME_PRESS)) %>%
pull(TIME_PRESS) %>%
{
tibble(
Statistic = c("Min", "Q1", "Median", "Q3", "Max",
"IQR", "Upper threshold (median + 1.5xIQR)",
"Lower threshold (median - 1.5xIQR)",
"IQR/Range", "Skewness", "Kurtosis",
"Shapiro-Wilk W", "Shapiro-Wilk p"),
Value = c(
round(min(.), 3),
round(quantile(., .25), 3),
round(median(.), 3),
round(quantile(., .75), 3),
round(max(.), 3),
round(IQR(.), 3),
round(median(.) + 1.5 * IQR(.), 3),
round(median(.) - 1.5 * IQR(.), 3),
round(IQR(.) / (max(.) - min(.)), 3),
round(datawizard::skewness(.)$Skewness, 3),
round(datawizard::kurtosis(.)$Kurtosis, 3),
round(shapiro.test(.)$statistic, 3),
format.pval(shapiro.test(.)$p.value, eps = .001)
)
)
} %>%
kable(caption = "TIME_PRESS normality — raw data")
```

TIME\_PRESS normality — raw data

| Statistic | Value |
| --- | --- |
| Min | 0.022 |
| Q1 | 1.851 |
| Median | 2.018 |
| Q3 | 2.07 |
| Max | 4.039 |
| IQR | 0.219 |
| Upper threshold (median + 1.5xIQR) | 2.346 |
| Lower threshold (median - 1.5xIQR) | 1.689 |
| IQR/Range | 0.054 |
| Skewness | -0.467 |
| Kurtosis | 1.574 |
| Shapiro-Wilk W | 0.81 |
| Shapiro-Wilk p | < 0.001 |

```
only_press <- data_PM %>%
filter(!is.na(TIME_PRESS)) %>%
mutate(
outlier =
TIME_PRESS > median(TIME_PRESS) + 1.5 * IQR(TIME_PRESS) |
TIME_PRESS < median(TIME_PRESS) - 1.5 * IQR(TIME_PRESS))

only_press %>%
filter(outlier == FALSE) %>%
pull(TIME_PRESS) %>%
{
tibble(
Statistic = c("Skewness", "Kurtosis", "Shapiro-Wilk W",
"Shapiro-Wilk p", "IQR/Range"),
Value = c(
round(datawizard::skewness(.)$Skewness, 3),
round(datawizard::kurtosis(.)$Kurtosis, 3),
round(shapiro.test(.)$statistic, 3),
format.pval(shapiro.test(.)$p.value, eps = .001),
round(IQR(.) / (max(.) - min(.)), 3)
)
)
} %>%
kable(caption = "TIME_PRESS normality — after TIME_PRESS outlier removal")
```

TIME\_PRESS normality — after TIME\_PRESS outlier
removal

| Statistic | Value |
| --- | --- |
| Skewness | -0.277 |
| Kurtosis | 2.352 |
| Shapiro-Wilk W | 0.93 |
| Shapiro-Wilk p | < 0.001 |
| IQR/Range | 0.107 |

```
# Escludi soggetto senza trial validi in una condizione
data_PM_press <- data_PM %>%
filter(!is.na(TIME_PRESS)) %>%
mutate(
outlier =
TIME_PRESS > median(TIME_PRESS, na.rm = TRUE) + 1.5 * IQR(TIME_PRESS, na.rm = TRUE) |
TIME_PRESS < median(TIME_PRESS, na.rm = TRUE) - 1.5 * IQR(TIME_PRESS, na.rm = TRUE)
) %>%
filter(outlier == FALSE) %>%
group_by(id) %>%
filter(n_distinct(stimulation) == 2) %>%
ungroup()
```

---

### TIME\_CHECK distribution — raw (after TIME\_PRESS removal)

Separate Tukey 1.5×IQR step applied to TIME\_CHECK within
`data_PM_check`; temporal quartile bins (0–.5 / .5–1 / 1–1.5
/ ≥1.5 min) assigned thereafter.

```
# Rimozione outlier TIME_PRESS e costruzione dataset checking
data_PM_check <-
data_PM %>%
left_join(
data_PM %>%
filter(!is.na(TIME_PRESS)) %>%
mutate(
outlier =
TIME_PRESS > median(TIME_PRESS) + 1.5 * IQR(TIME_PRESS) |
TIME_PRESS < median(TIME_PRESS) - 1.5 * IQR(TIME_PRESS)) %>%
distinct(id, stimulation, PROG_PRESS, outlier),
by = c("id", "stimulation", "PROG_PRESS")
) %>%
group_by(id, stimulation) %>%
mutate(
PROG_PRESS = ifelse(
is.na(PROG_PRESS),
ifelse(all(is.na(PROG_PRESS)), 1, max(PROG_PRESS, na.rm = TRUE) + 1),
PROG_PRESS)) %>%
group_by(id, stimulation, PROG_PRESS) %>%
mutate(outlier = ifelse(is.na(outlier), first(na.omit(outlier)), outlier)) %>%
filter(outlier == FALSE | is.na(outlier)) %>%
filter(!is.na(TIME_CHECK)) %>%
ungroup() %>%
filter(
TIME_CHECK < median(TIME_CHECK) + 1.5 * IQR(TIME_CHECK) &
TIME_CHECK > median(TIME_CHECK) - 1.5 * IQR(TIME_CHECK)) %>%
mutate(
QUARTILE = case_when(
TIME_CHECK < 0.5 ~ "1st",
TIME_CHECK < 1.0 ~ "2nd",
TIME_CHECK < 1.5 ~ "3rd",
TIME_CHECK >= 1.5 ~ "4th",
TRUE ~ NA_character_))

tibble(
Statistic = c("Skewness", "Kurtosis", "Shapiro-Wilk W", "Shapiro-Wilk p"),
Value = c(
round(datawizard::skewness(data_PM_check$TIME_CHECK)$Skewness, 3),
round(datawizard::kurtosis(data_PM_check$TIME_CHECK)$Kurtosis, 3),
round(shapiro.test(data_PM_check$TIME_CHECK)$statistic, 3),
format.pval(shapiro.test(data_PM_check$TIME_CHECK)$p.value, eps = .001)
)
) %>%
kable(caption = "TIME_CHECK normality — after TIME_CHECK outlier removal")
```

TIME\_CHECK normality — after TIME\_CHECK outlier
removal

| Statistic | Value |
| --- | --- |
| Skewness | -0.427 |
| Kurtosis | -0.912 |
| Shapiro-Wilk W | 0.948 |
| Shapiro-Wilk p | < 0.001 |

---

## CLOCK-CHECKING BEHAVIOR

### Check frequency (Poisson GLMM)

`data_PM_n_check`: counts of clock-checks per participant
× stimulation × quartile. Dataset built on `data_PM_check`
after double Tukey removal (123 cycles, 27 participants for checking
analyses per §2.4.5). N observations = 166.

```
data_PM_n_check <-
data_PM_check %>%
group_by(id, stimulation, QUARTILE) %>%
summarise(nCHECK = n(), guess = first(guess), .groups = "drop") %>%
mutate(
id = as.factor(id),
stimulation = as.factor(stimulation),
guess = as.factor(guess))

m_PM_CC_n_null <- glmer(
nCHECK ~ QUARTILE + guess + (1 | id),
family = poisson, data = data_PM_n_check,
control = glmerControl(optimizer = "bobyqa", optCtrl = list(maxfun = 100000)))

m_PM_CC_n_no_int <- glmer(
nCHECK ~ QUARTILE + stimulation + guess + (1 | id),
family = poisson, data = data_PM_n_check,
control = glmerControl(optimizer = "bobyqa", optCtrl = list(maxfun = 100000)))

m_PM_CC_n_int <- glmer(
nCHECK ~ QUARTILE * stimulation + guess + (1 | id),
family = poisson, data = data_PM_n_check,
control = glmerControl(optimizer = "bobyqa", optCtrl = list(maxfun = 100000)))

kable(as.data.frame(anova(m_PM_CC_n_null, m_PM_CC_n_no_int, m_PM_CC_n_int)),
caption = "LRT — clock-check frequency models", digits = 3)
```

LRT — clock-check frequency models

|  | npar | AIC | BIC | logLik | -2\*log(L) | Chisq | Df | Pr(>Chisq) |
| --- | --- | --- | --- | --- | --- | --- | --- | --- |
| m\_PM\_CC\_n\_null | 6 | 625.810 | 644.482 | -306.905 | 613.810 | NA | NA | NA |
| m\_PM\_CC\_n\_no\_int | 7 | 627.744 | 649.528 | -306.872 | 613.744 | 0.066 | 1 | 0.798 |
| m\_PM\_CC\_n\_int | 10 | 631.883 | 663.003 | -305.942 | 611.883 | 1.861 | 3 | 0.602 |

Null model retained (no stimulation effect). R² via Nakagawa &
Schielzeth.

```
summary(m_PM_CC_n_null)
```

```
## Generalized linear mixed model fit by maximum likelihood (Laplace
##   Approximation) [glmerMod]
##  Family: poisson  ( log )
## Formula: nCHECK ~ QUARTILE + guess + (1 | id)
##    Data: data_PM_n_check
## Control: glmerControl(optimizer = "bobyqa", optCtrl = list(maxfun = 1e+05))
## 
##       AIC       BIC    logLik -2*log(L)  df.resid 
##     625.8     644.5    -306.9     613.8       160 
## 
## Scaled residuals: 
##     Min      1Q  Median      3Q     Max 
## -1.1769 -0.4644 -0.1035  0.3733  1.9144 
## 
## Random effects:
##  Groups Name        Variance Std.Dev.
##  id     (Intercept) 0.1539   0.3923  
## Number of obs: 166, groups:  id, 27
## 
## Fixed effects:
##             Estimate Std. Error z value Pr(>|z|)    
## (Intercept)  0.73682    0.16698   4.413 1.02e-05 ***
## QUARTILE2nd  0.06538    0.16330   0.400   0.6889    
## QUARTILE3rd  0.27433    0.15666   1.751   0.0799 .  
## QUARTILE4th  0.81772    0.14667   5.575 2.47e-08 ***
## guess1      -0.04915    0.10142  -0.485   0.6279    
## ---
## Signif. codes:  0 '***' 0.001 '**' 0.01 '*' 0.05 '.' 0.1 ' ' 1
## 
## Correlation of Fixed Effects:
##             (Intr) QUARTILE2 QUARTILE3 QUARTILE4
## QUARTILE2nd -0.631                              
## QUARTILE3rd -0.665  0.681                       
## QUARTILE4th -0.718  0.728     0.764             
## guess1      -0.354 -0.038    -0.038    -0.032
```

```
kable(as.data.frame(car::Anova(m_PM_CC_n_null)),
caption = "Type II Wald chi-square tests — check frequency null model", digits = 3)
```

Type II Wald chi-square tests — check frequency null
model

|  | Chisq | Df | Pr(>Chisq) |
| --- | --- | --- | --- |
| QUARTILE | 65.760 | 3 | 0.000 |
| guess | 0.235 | 1 | 0.628 |

```
emm_quartile_n <- emmeans(m_PM_CC_n_null, specs = "QUARTILE", type = "response")

kable(as.data.frame(emm_quartile_n),
caption = "EMMs — check frequency per quartile (response scale, checks/cycle)",
digits = 3)
```

EMMs — check frequency per quartile (response scale,
checks/cycle)

| QUARTILE | rate | SE | df | asymp.LCL | asymp.UCL |
| --- | --- | --- | --- | --- | --- |
| 1st | 2.039 | 0.319 | Inf | 1.500 | 2.770 |
| 2nd | 2.176 | 0.276 | Inf | 1.698 | 2.790 |
| 3rd | 2.682 | 0.313 | Inf | 2.134 | 3.371 |
| 4th | 4.618 | 0.469 | Inf | 3.784 | 5.635 |

```
kable(as.data.frame(pairs(emm_quartile_n, adjust = "bonferroni")),
caption = "Pairwise contrasts — check frequency (ratio scale, Bonferroni)", digits = 3)
```

Pairwise contrasts — check frequency (ratio scale,
Bonferroni)

| contrast | ratio | SE | df | null | z.ratio | p.value |
| --- | --- | --- | --- | --- | --- | --- |
| 1st / 2nd | 0.937 | 0.153 | Inf | 1 | -0.400 | 1.000 |
| 1st / 3rd | 0.760 | 0.119 | Inf | 1 | -1.751 | 0.480 |
| 1st / 4th | 0.441 | 0.065 | Inf | 1 | -5.575 | 0.000 |
| 2nd / 3rd | 0.811 | 0.104 | Inf | 1 | -1.633 | 0.615 |
| 2nd / 4th | 0.471 | 0.054 | Inf | 1 | -6.521 | 0.000 |
| 3rd / 4th | 0.581 | 0.061 | Inf | 1 | -5.198 | 0.000 |

---

### Check timing (LMM)

Raw TIME\_CHECK is non-normal (skewness = −.427; kurtosis = −.912; W =
0.948, p < .001). In this version the model is estimated on the raw
scale (minutes). See §2.4.5.

```
m_PM_CC_t_null <- lmer(
TIME_CHECK ~ QUARTILE + guess + (1 | id),
data = data_PM_check)

m_PM_CC_t_no_int <- lmer(
TIME_CHECK ~ QUARTILE + stimulation + guess + (1 | id),
data = data_PM_check)

m_PM_CC_t_int <- lmer(
TIME_CHECK ~ QUARTILE * stimulation + guess + (1 | id),
data = data_PM_check)

kable(as.data.frame(anova(m_PM_CC_t_null, m_PM_CC_t_no_int, m_PM_CC_t_int)),
caption = "LRT — check timing models", digits = 3)
```

LRT — check timing models

|  | npar | AIC | BIC | logLik | -2\*log(L) | Chisq | Df | Pr(>Chisq) |
| --- | --- | --- | --- | --- | --- | --- | --- | --- |
| m\_PM\_CC\_t\_null | 7 | -487.759 | -457.438 | 250.879 | -501.759 | NA | NA | NA |
| m\_PM\_CC\_t\_no\_int | 8 | -486.202 | -451.550 | 251.101 | -502.202 | 0.443 | 1 | 0.506 |
| m\_PM\_CC\_t\_int | 11 | -480.309 | -432.662 | 251.154 | -502.309 | 0.107 | 3 | 0.991 |

Null model retained. Stimulation does not modulate check timing
(§3.5).

```
summary(m_PM_CC_t_null)
```

```
## Linear mixed model fit by REML ['lmerMod']
## Formula: TIME_CHECK ~ QUARTILE + guess + (1 | id)
##    Data: data_PM_check
## 
## REML criterion at convergence: -468.7
## 
## Scaled residuals: 
##     Min      1Q  Median      3Q     Max 
## -2.1258 -0.7648  0.1343  0.8291  3.0168 
## 
## Random effects:
##  Groups   Name        Variance Std.Dev.
##  id       (Intercept) 0.000505 0.02247 
##  Residual             0.023789 0.15424 
## Number of obs: 562, groups:  id, 27
## 
## Fixed effects:
##             Estimate Std. Error t value
## (Intercept)  0.32688    0.02302  14.200
## QUARTILE2nd  0.44370    0.02527  17.558
## QUARTILE3rd  0.92344    0.02438  37.882
## QUARTILE4th  1.49830    0.02289  65.445
## guess        0.01594    0.01430   1.115
## 
## Correlation of Fixed Effects:
##             (Intr) QUARTILE2 QUARTILE3 QUARTILE4
## QUARTILE2nd -0.722                              
## QUARTILE3rd -0.763  0.681                       
## QUARTILE4th -0.820  0.729     0.765             
## guess       -0.378 -0.013     0.000     0.007
```

```
kable(as.data.frame(car::Anova(m_PM_CC_t_no_int)),
caption = "Type II Wald chi-square tests — stimulation effect on check timing", digits = 3)
```

Type II Wald chi-square tests — stimulation effect on check
timing

|  | Chisq | Df | Pr(>Chisq) |
| --- | --- | --- | --- |
| QUARTILE | 6261.295 | 3 | 0.000 |
| stimulation | 0.447 | 1 | 0.504 |
| guess | 0.136 | 1 | 0.712 |

```
emm_quartile_t <- emmeans(m_PM_CC_t_null, specs = "QUARTILE")

kable(as.data.frame(emm_quartile_t),
caption = "EMMs — check timing per quartile (minutes)", digits = 3)
```

EMMs — check timing per quartile (minutes)

| QUARTILE | emmean | SE | df | lower.CL | upper.CL |
| --- | --- | --- | --- | --- | --- |
| 1st | 0.335 | 0.022 | 253.751 | 0.292 | 0.377 |
| 2nd | 0.779 | 0.016 | 204.441 | 0.747 | 0.810 |
| 3rd | 1.258 | 0.014 | 156.088 | 1.231 | 1.286 |
| 4th | 1.833 | 0.011 | 65.137 | 1.811 | 1.855 |

```
kable(as.data.frame(pairs(emm_quartile_t, adjust = "bonferroni")),
caption = "Pairwise contrasts — check timing (minutes, Bonferroni)", digits = 3)
```

Pairwise contrasts — check timing (minutes,
Bonferroni)

| contrast | estimate | SE | df | t.ratio | p.value |
| --- | --- | --- | --- | --- | --- |
| 1st - 2nd | -0.444 | 0.025 | 556.829 | -17.474 | 0 |
| 1st - 3rd | -0.923 | 0.025 | 548.082 | -37.589 | 0 |
| 1st - 4th | -1.498 | 0.023 | 524.202 | -64.741 | 0 |
| 2nd - 3rd | -0.480 | 0.020 | 551.950 | -24.141 | 0 |
| 2nd - 4th | -1.055 | 0.018 | 553.421 | -58.926 | 0 |
| 3rd - 4th | -0.575 | 0.016 | 546.577 | -35.339 | 0 |

---

## PM ACCURACY ANALYSIS

### Data Preparation

Button press classified as correct if |TIME\_PRESS − 2| ≤ 0.1 min (±6
s). Clock-check count per cycle (n\_CHECK) computed from
`data_PM` prior to TIME\_PRESS outlier removal. Covariate
transformed as `log(n_CHECK + 1)`. Box-Cox transformation
applied to `err_abs`; lambda estimated via MLE using
`MASS::boxcox` (see §2.4.4).

`data_PM_pata_acc`: 123 obs × 26 participants — id,
stimulation, correct (lgl), err\_abs (min), n\_CHECK (int), guess
(fct).

```
data_PM_pata_acc <-
data_PM %>%
filter(!is.na(TIME_PRESS)) %>%
mutate(
outlier =
TIME_PRESS > median(TIME_PRESS, na.rm = TRUE) + 1.5 * IQR(TIME_PRESS, na.rm = TRUE) |
TIME_PRESS < median(TIME_PRESS, na.rm = TRUE) - 1.5 * IQR(TIME_PRESS, na.rm = TRUE),
correct = (abs(TIME_PRESS - 2) <= 0.1),
err_abs = abs(TIME_PRESS - 2)) %>%
mutate(
outlier = ifelse(
id %in% (
filter(., outlier == FALSE) %>%
count(id, stimulation) %>%
tidyr::pivot_wider(names_from = stimulation, values_from = n,
values_fill = list(n = 0)) %>%
filter(beta == 0 | sham == 0) %>%
pull(id)),
TRUE, outlier)) %>%
filter(outlier == FALSE) %>%
left_join(
data_PM %>%
group_by(id, stimulation, PROG_PRESS) %>%
mutate(n_CHECK = sum(!is.na(TIME_CHECK))) %>%
filter(!is.na(TIME_PRESS)) %>%
distinct(id, stimulation, PROG_PRESS, n_CHECK),
by = c("id", "stimulation", "PROG_PRESS")) %>%
ungroup() %>%
mutate(guess = as.factor(guess), id = as.factor(id))
```

---

### Normality Assessment and Box-Cox Transformation

```
boxcox_result <- MASS::boxcox(
lm(data_PM_pata_acc$err_abs ~ 1),
lambda = seq(0, 2, 0.001),
plotit = FALSE)

lambda_err <- boxcox_result$x[boxcox_result$y == max(boxcox_result$y)]

data_PM_pata_acc <- data_PM_pata_acc %>%
mutate(
err_abs_t = err_abs^lambda_err,
n_CHECK_t = log(n_CHECK + 1))

tibble(
Variable = c("err_abs (raw)", paste0("err_abs (Box-Cox, lambda = ", round(lambda_err, 2), ")")),
Skewness = c(
round(datawizard::skewness(data_PM_pata_acc$err_abs)$Skewness, 3),
round(datawizard::skewness(data_PM_pata_acc$err_abs_t)$Skewness, 3)),
Kurtosis = c(
round(datawizard::kurtosis(data_PM_pata_acc$err_abs)$Kurtosis, 3),
round(datawizard::kurtosis(data_PM_pata_acc$err_abs_t)$Kurtosis, 3)),
`Shapiro-Wilk W` = c(
round(shapiro.test(data_PM_pata_acc$err_abs)$statistic, 3),
round(shapiro.test(data_PM_pata_acc$err_abs_t)$statistic, 3)),
`Shapiro-Wilk p` = c(
format.pval(shapiro.test(data_PM_pata_acc$err_abs)$p.value, eps = .001),
format.pval(shapiro.test(data_PM_pata_acc$err_abs_t)$p.value, eps = .001))
) %>%
kable(caption = "Normality diagnostics — err_abs transformations")
```

Normality diagnostics — err\_abs transformations

| Variable | Skewness | Kurtosis | Shapiro-Wilk W | Shapiro-Wilk p |
| --- | --- | --- | --- | --- |
| err\_abs (raw) | 1.669 | 2.396 | 0.808 | < 0.001 |
| err\_abs (Box-Cox, lambda = 0.24) | -0.019 | -0.133 | 0.990 | 0.48134 |

```
tibble(
Variable = c("n_CHECK (raw)", "log(n_CHECK + 1)"),
Skewness = c(
round(datawizard::skewness(data_PM_pata_acc$n_CHECK)$Skewness, 3),
round(datawizard::skewness(data_PM_pata_acc$n_CHECK_t)$Skewness, 3)),
Kurtosis = c(
round(datawizard::kurtosis(data_PM_pata_acc$n_CHECK)$Kurtosis, 3),
round(datawizard::kurtosis(data_PM_pata_acc$n_CHECK_t)$Kurtosis, 3)),
`Shapiro-Wilk W` = c(
round(shapiro.test(data_PM_pata_acc$n_CHECK)$statistic, 3),
round(shapiro.test(data_PM_pata_acc$n_CHECK_t)$statistic, 3))
) %>%
kable(caption = "Normality diagnostics — n_CHECK transformations")
```

Normality diagnostics — n\_CHECK transformations

| Variable | Skewness | Kurtosis | Shapiro-Wilk W |
| --- | --- | --- | --- |
| n\_CHECK (raw) | 1.281 | 2.569 | 0.90 |
| log(n\_CHECK + 1) | -0.544 | 1.018 | 0.95 |

---

### Dichotomous Accuracy (GLMM)

```
m_PM_BP_c_null <- glmer(
correct ~ n_CHECK_t + guess + (1 | id),
family = binomial, data = data_PM_pata_acc)

m_PM_BP_c_no_int <- glmer(
correct ~ stimulation + n_CHECK_t + guess + (1 | id),
family = binomial, data = data_PM_pata_acc)

kable(as.data.frame(anova(m_PM_BP_c_null, m_PM_BP_c_no_int)),
caption = "LRT — dichotomous accuracy models", digits = 3)
```

LRT — dichotomous accuracy models

|  | npar | AIC | BIC | logLik | -2\*log(L) | Chisq | Df | Pr(>Chisq) |
| --- | --- | --- | --- | --- | --- | --- | --- | --- |
| m\_PM\_BP\_c\_null | 4 | 113.851 | 125.100 | -52.926 | 105.851 | NA | NA | NA |
| m\_PM\_BP\_c\_no\_int | 5 | 106.478 | 120.539 | -48.239 | 96.478 | 9.373 | 1 | 0.002 |

Stimulation model retained (χ²(1) = 9.37, p = .002). R² via Nakagawa
& Schielzeth.

```
summary(m_PM_BP_c_no_int)
```

```
## Generalized linear mixed model fit by maximum likelihood (Laplace
##   Approximation) [glmerMod]
##  Family: binomial  ( logit )
## Formula: correct ~ stimulation + n_CHECK_t + guess + (1 | id)
##    Data: data_PM_pata_acc
## 
##       AIC       BIC    logLik -2*log(L)  df.resid 
##     106.5     120.5     -48.2      96.5       118 
## 
## Scaled residuals: 
##     Min      1Q  Median      3Q     Max 
## -4.3838  0.0493  0.2489  0.3727  3.6107 
## 
## Random effects:
##  Groups Name        Variance Std.Dev.
##  id     (Intercept) 0.7882   0.8878  
## Number of obs: 123, groups:  id, 26
## 
## Fixed effects:
##                 Estimate Std. Error z value Pr(>|z|)    
## (Intercept)       0.0629     1.2154   0.052  0.95872    
## stimulationsham  -2.1361     0.7572  -2.821  0.00479 ** 
## n_CHECK_t         2.8517     0.7719   3.695  0.00022 ***
## guess1           -2.5827     0.8789  -2.939  0.00330 ** 
## ---
## Signif. codes:  0 '***' 0.001 '**' 0.01 '*' 0.05 '.' 0.1 ' ' 1
## 
## Correlation of Fixed Effects:
##             (Intr) stmltn n_CHEC
## stimultnshm -0.382              
## n_CHECK_t   -0.623 -0.315       
## guess1      -0.513  0.656 -0.253
```

```
kable(as.data.frame(car::Anova(m_PM_BP_c_no_int)),
caption = "Type II Wald chi-square tests — dichotomous accuracy model", digits = 3)
```

Type II Wald chi-square tests — dichotomous accuracy
model

|  | Chisq | Df | Pr(>Chisq) |
| --- | --- | --- | --- |
| stimulation | 7.958 | 1 | 0.005 |
| n\_CHECK\_t | 13.650 | 1 | 0.000 |
| guess | 8.636 | 1 | 0.003 |

```
sjPlot::tab_model(
m_PM_BP_c_no_int,
string.pred = "Effect", string.est = "β", string.ci = "95% CI", string.p = "p")
```

|  | correct | | |
| --- | --- | --- | --- |
| Effect | β | 95% CI | p |
| (Intercept) | 1.06 | 0.10 – 11.53 | 0.959 |
| stimulation [sham] | 0.12 | 0.03 – 0.52 | **0.005** |
| n CHECK t | 17.32 | 3.81 – 78.61 | **<0.001** |
| guess [1] | 0.08 | 0.01 – 0.42 | **0.003** |
| Random Effects | | | |
| σ^2^ | 3.29 | | |
| τ_00_ _id_ | 0.79 | | || ICC | 0.19 | | || N _id_ | 26 | | || Observations | 123 | | |
| Marginal R^2^ / Conditional R^2^ | 0.427 / 0.537 | | |

```
emm_stim_c <- emmeans(m_PM_BP_c_no_int, specs = "stimulation")

kable(as.data.frame(emm_stim_c),
caption = "EMMs — dichotomous accuracy by stimulation (logit scale)", digits = 3)
```

EMMs — dichotomous accuracy by stimulation (logit
scale)

| stimulation | emmean | SE | df | asymp.LCL | asymp.UCL |
| --- | --- | --- | --- | --- | --- |
| beta | 3.077 | 0.675 | Inf | 1.754 | 4.401 |
| sham | 0.941 | 0.454 | Inf | 0.051 | 1.832 |

```
kable(as.data.frame(emmeans(m_PM_BP_c_no_int, specs = "stimulation", type = "response")),
caption = "EMMs — dichotomous accuracy by stimulation (probability scale)", digits = 3)
```

EMMs — dichotomous accuracy by stimulation (probability
scale)

| stimulation | prob | SE | df | asymp.LCL | asymp.UCL |
| --- | --- | --- | --- | --- | --- |
| beta | 0.956 | 0.028 | Inf | 0.852 | 0.988 |
| sham | 0.719 | 0.092 | Inf | 0.513 | 0.862 |

```
kable(as.data.frame(pairs(emm_stim_c, adjust = "bonferroni")),
caption = "Pairwise contrast: beta vs sham — dichotomous accuracy (log-OR scale)",
digits = 3)
```

Pairwise contrast: beta vs sham — dichotomous accuracy (log-OR
scale)

| contrast | estimate | SE | df | z.ratio | p.value |
| --- | --- | --- | --- | --- | --- |
| beta - sham | 2.136 | 0.757 | Inf | 2.821 | 0.005 |

```
emm_guess_c <- emmeans(m_PM_BP_c_no_int, specs = "guess")

kable(as.data.frame(pairs(emm_guess_c, adjust = "bonferroni")),
caption = "Pairwise contrast: guess condition — dichotomous accuracy", digits = 3)
```

Pairwise contrast: guess condition — dichotomous
accuracy

| contrast | estimate | SE | df | z.ratio | p.value |
| --- | --- | --- | --- | --- | --- |
| guess0 - guess1 | 2.583 | 0.879 | Inf | 2.939 | 0.003 |

---

### Continuous Accuracy (LMM)

Absolute temporal error (err\_abs) Box-Cox-transformed (λ ≈ 1.3;
post-transformation: skewness = −0.019, kurtosis = −0.133, W = .99, p =
.481). See §2.4.4.

```
m_PM_BP_t_null <- lmer(
err_abs_t ~ n_CHECK_t + guess + (1 | id),
data = data_PM_pata_acc)

m_PM_BP_t_no_int <- lmer(
err_abs_t ~ stimulation + n_CHECK_t + guess + (1 | id),
data = data_PM_pata_acc)

kable(as.data.frame(anova(m_PM_BP_t_null, m_PM_BP_t_no_int)),
caption = "LRT — continuous accuracy models", digits = 3)
```

LRT — continuous accuracy models

|  | npar | AIC | BIC | logLik | -2\*log(L) | Chisq | Df | Pr(>Chisq) |
| --- | --- | --- | --- | --- | --- | --- | --- | --- |
| m\_PM\_BP\_t\_null | 5 | -181.524 | -167.463 | 95.762 | -191.524 | NA | NA | NA |
| m\_PM\_BP\_t\_no\_int | 6 | -186.012 | -169.139 | 99.006 | -198.012 | 6.489 | 1 | 0.011 |

Stimulation model retained (χ²(1) = 6.49, p = .011).

```
summary(m_PM_BP_t_no_int)
```

```
## Linear mixed model fit by REML ['lmerMod']
## Formula: err_abs_t ~ stimulation + n_CHECK_t + guess + (1 | id)
##    Data: data_PM_pata_acc
## 
## REML criterion at convergence: -174.5
## 
## Scaled residuals: 
##     Min      1Q  Median      3Q     Max 
## -2.0849 -0.5939  0.0919  0.6574  2.0160 
## 
## Random effects:
##  Groups   Name        Variance Std.Dev.
##  id       (Intercept) 0.003621 0.06017 
##  Residual             0.009798 0.09899 
## Number of obs: 123, groups:  id, 26
## 
## Fixed effects:
##                 Estimate Std. Error t value
## (Intercept)      0.53157    0.05046  10.535
## stimulationsham  0.06714    0.02650   2.534
## n_CHECK_t       -0.08839    0.02506  -3.527
## guess1           0.08638    0.03088   2.798
## 
## Correlation of Fixed Effects:
##             (Intr) stmltn n_CHEC
## stimultnshm -0.515              
## n_CHECK_t   -0.746 -0.027       
## guess1      -0.595  0.730  0.030
```

```
kable(as.data.frame(car::Anova(m_PM_BP_t_no_int)),
caption = "Type II Wald chi-square tests — continuous accuracy model", digits = 3)
```

Type II Wald chi-square tests — continuous accuracy
model

|  | Chisq | Df | Pr(>Chisq) |
| --- | --- | --- | --- |
| stimulation | 6.421 | 1 | 0.011 |
| n\_CHECK\_t | 12.437 | 1 | 0.000 |
| guess | 7.827 | 1 | 0.005 |

```
sjPlot::tab_model(
m_PM_BP_t_no_int,
string.pred = "Effect", string.est = "β", string.ci = "95% CI", string.p = "p")
```

|  | err\_abs\_t | | |
| --- | --- | --- | --- |
| Effect | β | 95% CI | p |
| (Intercept) | 0.53 | 0.43 – 0.63 | **<0.001** |
| stimulation [sham] | 0.07 | 0.01 – 0.12 | **0.013** |
| n CHECK t | -0.09 | -0.14 – -0.04 | **0.001** |
| guess [1] | 0.09 | 0.03 – 0.15 | **0.006** |
| Random Effects | | | |
| σ^2^ | 0.01 | | |
| τ_00_ _id_ | 0.00 | | || ICC | 0.27 | | || N _id_ | 26 | | || Observations | 123 | | |
| Marginal R^2^ / Conditional R^2^ | 0.185 / 0.405 | | |

```
emm_stim_t <- emmeans(m_PM_BP_t_no_int, specs = "stimulation")

kable(as.data.frame(emm_stim_t),
caption = "EMMs — continuous accuracy by stimulation (Box-Cox scale)", digits = 3)
```

EMMs — continuous accuracy by stimulation (Box-Cox
scale)

| stimulation | emmean | SE | df | lower.CL | upper.CL |
| --- | --- | --- | --- | --- | --- |
| beta | 0.441 | 0.022 | 60.729 | 0.397 | 0.485 |
| sham | 0.508 | 0.019 | 48.178 | 0.471 | 0.546 |

```
kable(as.data.frame(pairs(emm_stim_t, adjust = "bonferroni")),
caption = "Pairwise contrast: beta vs sham — continuous accuracy", digits = 3)
```

Pairwise contrast: beta vs sham — continuous accuracy

| contrast | estimate | SE | df | t.ratio | p.value |
| --- | --- | --- | --- | --- | --- |
| beta - sham | -0.067 | 0.027 | 118.504 | -2.502 | 0.014 |

```
emm_guess_t <- emmeans(m_PM_BP_t_no_int, specs = "guess")

kable(as.data.frame(pairs(emm_guess_t, adjust = "bonferroni")),
caption = "Pairwise contrast: guess condition — continuous accuracy", digits = 3)
```

Pairwise contrast: guess condition — continuous
accuracy

| contrast | estimate | SE | df | t.ratio | p.value |
| --- | --- | --- | --- | --- | --- |
| guess0 - guess1 | -0.086 | 0.032 | 108.19 | -2.733 | 0.007 |

---

## ROBUSTNESS ANALYSIS

*This section extends the PM accuracy analyses above with a
covariate-adjusted robustness check of the stimulation effect on both
dichotomous and continuous accuracy.*

Additional variables accounted for: task order (`task`; 2
levels: MTT-first vs. DD&PM-first), session order
(`session`; 2 levels: ‘1st’ vs ‘2nd’), and self-reported side
effects of stimulation (itching - `itch`, pain -
`pain`, burning - `burn`, tingling -
`ting`, fatigue - `fatg`, other -
`oth`), phosphene perception (`phos`,
dichotomic).

### Covariate-Adjusted Dichotomous Accuracy Model

The winning model (`m_PM_BP_c_no_int`, above) is
re-estimated with task order, session, and self-reported side
effects/phosphenes added as covariates.

```
m_PM_BP_c_no_int_robust <- glmer(
correct ~ stimulation + n_CHECK_t + guess.x + (1 | id) +
as.factor(task) + as.factor(session) + as.factor(phos) + itch + pain + burn + ting + fatg + oth,
family = binomial, data = data_PM_pata_acc)

summary(m_PM_BP_c_no_int_robust)
```

```
## Generalized linear mixed model fit by maximum likelihood (Laplace
##   Approximation) [glmerMod]
##  Family: binomial  ( logit )
## Formula: 
## correct ~ stimulation + n_CHECK_t + guess.x + (1 | id) + as.factor(task) +  
##     as.factor(session) + as.factor(phos) + itch + pain + burn +  
##     ting + fatg + oth
##    Data: data_PM_pata_acc
## 
##       AIC       BIC    logLik -2*log(L)  df.resid 
##     110.9     150.3     -41.5      82.9       109 
## 
## Scaled residuals: 
##     Min      1Q  Median      3Q     Max 
## -7.7584  0.0185  0.1781  0.3626  1.2597 
## 
## Random effects:
##  Groups Name        Variance Std.Dev.
##  id     (Intercept) 0.8115   0.9009  
## Number of obs: 123, groups:  id, 26
## 
## Fixed effects:
##                          Estimate Std. Error z value Pr(>|z|)    
## (Intercept)               -0.2563     1.7355  -0.148 0.882585    
## stimulationsham           -2.4798     1.0221  -2.426 0.015258 *  
## n_CHECK_t                  4.3526     1.2815   3.396 0.000683 ***
## guess.x1                  -2.1253     1.0411  -2.041 0.041208 *  
## as.factor(task)MTT_first  -2.0008     0.8276  -2.418 0.015616 *  
## as.factor(session)second  -1.7648     0.9594  -1.839 0.065862 .  
## as.factor(phos)1          -3.8065     1.6342  -2.329 0.019844 *  
## itch                      -0.5445     1.0548  -0.516 0.605691    
## pain                       2.6994     1.9224   1.404 0.160275    
## burn                      -0.7798     0.8535  -0.914 0.360885    
## ting                       0.8745     1.1506   0.760 0.447233    
## fatg                       0.3375     0.7331   0.460 0.645248    
## oth                        1.3490     0.6513   2.071 0.038345 *  
## ---
## Signif. codes:  0 '***' 0.001 '**' 0.01 '*' 0.05 '.' 0.1 ' ' 1
```

```
## optimizer (Nelder_Mead) convergence code: 0 (OK)
## Model failed to converge with max|grad| = 0.063569 (tol = 0.002, component 1)
##   See ?lme4::convergence and ?lme4::troubleshooting.
```

```
kable(as.data.frame(car::Anova(m_PM_BP_c_no_int_robust)),
caption = "Type II Wald chi-square tests — dichotomous accuracy, covariate-adjusted model",
digits = 3)
```

Type II Wald chi-square tests — dichotomous accuracy,
covariate-adjusted model

|  | Chisq | Df | Pr(>Chisq) |
| --- | --- | --- | --- |
| stimulation | 5.886 | 1 | 0.015 |
| n\_CHECK\_t | 11.536 | 1 | 0.001 |
| guess.x | 4.167 | 1 | 0.041 |
| as.factor(task) | 5.846 | 1 | 0.016 |
| as.factor(session) | 3.383 | 1 | 0.066 |
| as.factor(phos) | 5.426 | 1 | 0.020 |
| itch | 0.266 | 1 | 0.606 |
| pain | 1.972 | 1 | 0.160 |
| burn | 0.835 | 1 | 0.361 |
| ting | 0.578 | 1 | 0.447 |
| fatg | 0.212 | 1 | 0.645 |
| oth | 4.290 | 1 | 0.038 |

```
sjPlot::tab_model(
m_PM_BP_c_no_int_robust,
string.pred = "Effect", string.est = "β", string.ci = "95% CI", string.p = "p")
```

|  | correct | | |
| --- | --- | --- | --- |
| Effect | β | 95% CI | p |
| (Intercept) | 0.77 | 0.03 – 23.22 | 0.883 |
| stimulation [sham] | 0.08 | 0.01 – 0.62 | **0.015** |
| n CHECK t | 77.68 | 6.30 – 957.51 | **0.001** |
| guess x [1] | 0.12 | 0.02 – 0.92 | **0.041** |
| task [MTT\_first] | 0.14 | 0.03 – 0.68 | **0.016** |
| session [second] | 0.17 | 0.03 – 1.12 | 0.066 |
| phos [1] | 0.02 | 0.00 – 0.55 | **0.020** |
| itch | 0.58 | 0.07 – 4.59 | 0.606 |
| pain | 14.87 | 0.34 – 643.73 | 0.160 |
| burn | 0.46 | 0.09 – 2.44 | 0.361 |
| ting | 2.40 | 0.25 – 22.86 | 0.447 |
| fatg | 1.40 | 0.33 – 5.90 | 0.645 |
| oth | 3.85 | 1.08 – 13.81 | **0.038** |
| Random Effects | | | |
| σ^2^ | 3.29 | | |
| τ_00_ _id_ | 0.81 | | || ICC | 0.20 | | || N _id_ | 26 | | || Observations | 123 | | |
| Marginal R^2^ / Conditional R^2^ | 0.602 / 0.681 | | |

```
emm_stim_c_robust <- emmeans(m_PM_BP_c_no_int_robust, specs = "stimulation")

kable(as.data.frame(emm_stim_c_robust),
caption = "EMMs — dichotomous accuracy (covariate-adjusted) by stimulation (logit scale)", digits = 3)
```

EMMs — dichotomous accuracy (covariate-adjusted) by stimulation
(logit scale)

| stimulation | emmean | SE | df | asymp.LCL | asymp.UCL |
| --- | --- | --- | --- | --- | --- |
| beta | 3.818 | 1.285 | Inf | 1.300 | 6.336 |
| sham | 1.338 | 0.938 | Inf | -0.501 | 3.177 |

```
kable(as.data.frame(emmeans(m_PM_BP_c_no_int_robust, specs = "stimulation", type = "response")),
caption = "EMMs — dichotomous accuracy (covariate-adjusted) by stimulation (probability scale)",
digits = 3)
```

EMMs — dichotomous accuracy (covariate-adjusted) by stimulation
(probability scale)

| stimulation | prob | SE | df | asymp.LCL | asymp.UCL |
| --- | --- | --- | --- | --- | --- |
| beta | 0.978 | 0.027 | Inf | 0.786 | 0.998 |
| sham | 0.792 | 0.154 | Inf | 0.377 | 0.960 |

```
kable(as.data.frame(pairs(emm_stim_c_robust, adjust = "bonferroni")),
caption = "Pairwise contrast: beta vs sham — dichotomous accuracy, covariate-adjusted (log-OR scale)",
digits = 3)
```

Pairwise contrast: beta vs sham — dichotomous accuracy,
covariate-adjusted (log-OR scale)

| contrast | estimate | SE | df | z.ratio | p.value |
| --- | --- | --- | --- | --- | --- |
| beta - sham | 2.48 | 1.022 | Inf | 2.426 | 0.015 |

### Covariate-Adjusted Continuous Accuracy Model

The winning model (`m_PM_BP_t_no_int`, above) is
re-estimated with the same order and side-effect covariates.

```
m_PM_BP_t_no_int_robust <- lmer(
err_abs_t ~ stimulation + n_CHECK_t + guess.x + (1 | id) +
as.factor(task) + as.factor(session) + as.factor(phos) + itch + pain + burn + ting + fatg + oth,
data = data_PM_pata_acc)

summary(m_PM_BP_t_no_int_robust)
```

```
## Linear mixed model fit by REML ['lmerMod']
## Formula: 
## err_abs_t ~ stimulation + n_CHECK_t + guess.x + (1 | id) + as.factor(task) +  
##     as.factor(session) + as.factor(phos) + itch + pain + burn +  
##     ting + fatg + oth
##    Data: data_PM_pata_acc
## 
## REML criterion at convergence: -137.4
## 
## Scaled residuals: 
##      Min       1Q   Median       3Q      Max 
## -2.18230 -0.59997  0.07849  0.68936  2.17619 
## 
## Random effects:
##  Groups   Name        Variance Std.Dev.
##  id       (Intercept) 0.004703 0.06858 
##  Residual             0.009146 0.09564 
## Number of obs: 123, groups:  id, 26
## 
## Fixed effects:
##                            Estimate Std. Error t value
## (Intercept)               0.5605526  0.0550807  10.177
## stimulationsham           0.0676183  0.0302380   2.236
## n_CHECK_t                -0.0935862  0.0275939  -3.392
## guess.x1                  0.0885858  0.0377493   2.347
## as.factor(task)MTT_first -0.0005445  0.0199932  -0.027
## as.factor(session)second  0.0079853  0.0235517   0.339
## as.factor(phos)1          0.0587304  0.0445403   1.319
## itch                     -0.0063518  0.0345650  -0.184
## pain                     -0.1183355  0.0571679  -2.070
## burn                      0.0384635  0.0284904   1.350
## ting                     -0.0592995  0.0352185  -1.684
## fatg                      0.0119280  0.0247024   0.483
## oth                      -0.0290707  0.0203832  -1.426
```

```
kable(as.data.frame(car::Anova(m_PM_BP_t_no_int_robust)),
caption = "Type II Wald chi-square tests — continuous accuracy, covariate-adjusted model",
digits = 3)
```

Type II Wald chi-square tests — continuous accuracy,
covariate-adjusted model

|  | Chisq | Df | Pr(>Chisq) |
| --- | --- | --- | --- |
| stimulation | 5.001 | 1 | 0.025 |
| n\_CHECK\_t | 11.503 | 1 | 0.001 |
| guess.x | 5.507 | 1 | 0.019 |
| as.factor(task) | 0.001 | 1 | 0.978 |
| as.factor(session) | 0.115 | 1 | 0.735 |
| as.factor(phos) | 1.739 | 1 | 0.187 |
| itch | 0.034 | 1 | 0.854 |
| pain | 4.285 | 1 | 0.038 |
| burn | 1.823 | 1 | 0.177 |
| ting | 2.835 | 1 | 0.092 |
| fatg | 0.233 | 1 | 0.629 |
| oth | 2.034 | 1 | 0.154 |

```
sjPlot::tab_model(
m_PM_BP_t_no_int_robust,
string.pred = "Effect", string.est = "β", string.ci = "95% CI", string.p = "p")
```

|  | err\_abs\_t | | |
| --- | --- | --- | --- |
| Effect | β | 95% CI | p |
| (Intercept) | 0.56 | 0.45 – 0.67 | **<0.001** |
| stimulation [sham] | 0.07 | 0.01 – 0.13 | **0.027** |
| n CHECK t | -0.09 | -0.15 – -0.04 | **0.001** |
| guess x [1] | 0.09 | 0.01 – 0.16 | **0.021** |
| task [MTT\_first] | -0.00 | -0.04 – 0.04 | 0.978 |
| session [second] | 0.01 | -0.04 – 0.05 | 0.735 |
| phos [1] | 0.06 | -0.03 – 0.15 | 0.190 |
| itch | -0.01 | -0.07 – 0.06 | 0.855 |
| pain | -0.12 | -0.23 – -0.01 | **0.041** |
| burn | 0.04 | -0.02 – 0.09 | 0.180 |
| ting | -0.06 | -0.13 – 0.01 | 0.095 |
| fatg | 0.01 | -0.04 – 0.06 | 0.630 |
| oth | -0.03 | -0.07 – 0.01 | 0.157 |
| Random Effects | | | |
| σ^2^ | 0.01 | | |
| τ_00_ _id_ | 0.00 | | || ICC | 0.34 | | || N _id_ | 26 | | || Observations | 123 | | |
| Marginal R^2^ / Conditional R^2^ | 0.252 / 0.506 | | |

```
emm_stim_t_robust <- emmeans(m_PM_BP_t_no_int_robust, specs = "stimulation")

kable(as.data.frame(emm_stim_t_robust),
caption = "EMMs — continuous accuracy (covariate-adjusted) by stimulation (Box-Cox scale)", digits = 3)
```

EMMs — continuous accuracy (covariate-adjusted) by stimulation
(Box-Cox scale)

| stimulation | emmean | SE | df | lower.CL | upper.CL |
| --- | --- | --- | --- | --- | --- |
| beta | 0.401 | 0.040 | 68.383 | 0.321 | 0.482 |
| sham | 0.469 | 0.032 | 84.480 | 0.405 | 0.532 |

```
kable(as.data.frame(pairs(emm_stim_t_robust, adjust = "bonferroni")),
caption = "Pairwise contrast: beta vs sham — continuous accuracy, covariate-adjusted", digits = 3)
```

Pairwise contrast: beta vs sham — continuous accuracy,
covariate-adjusted

| contrast | estimate | SE | df | t.ratio | p.value |
| --- | --- | --- | --- | --- | --- |
| beta - sham | -0.068 | 0.031 | 107.438 | -2.198 | 0.03 |
