# Supplementary material for: Beta-Band Stimulation of Dorsolateral Prefrontal Cortex Through tACS Alters Mental Time Travel and Prospective Memory, but Not Delay Discounting
Source: Brain Sci. 2026 Aug 22;16(9):899. doi: 10.3390/brainsci16090899 (PMC13604682; doi:10.3390/brainsci16090899)
Supplement: Supplementary file 1 [file brainsci-16-00899-s001.zip › S7 Personality (code markdown).html]

tACS\_future\_CORRELATIONS


# tACS\_future\_CORRELATIONS

#### Authors are masked for peer review purposes

## CAT Questionnaire — Loading and Scoring

The CAT questionnaire composite scores are computed for each of the 8
theoretically motivated subscales by summing the relevant item
scores.

```
# .$CAT_FA  : Future Anticipatory Anxiety          (3 items)
# .$CAT_FP  : Future Positive Anticipation         (4 items)
# .$CAT_PF  : Psychological Fear (Future)          (4 items)
# .$CAT_PR  : Past Regret                          (9 items)
# .$CAT_PH  : Past Happiness                       (5 items)
# .$CAT_PNe : Past Negative Nostalgia              (3 items)
# .$CAT_PNo : Past Nostalgia                       (8 items)
# .$CAT_PE  : Present Engagement                  (4 items)

CAT_composite <-
  CAT_data %>%
  reframe(
    subject_id = subject_id,
    CAT_FA  = rowSums(across(all_of(c("FuNeCo_3", "FuNeCo_4", "FuNeNC_2"))),                                                                               na.rm = TRUE),
    CAT_FP  = rowSums(across(all_of(c("FuPoCo_4", "FuPoCo_1", "FuPoCo_2", "FuPoCo_3"))),                                                                  na.rm = TRUE),
    CAT_PF  = rowSums(across(all_of(c("FuNeNC_1", "FuNeNC_4", "PrNeNC_3", "PrNeNC_1"))),                                                                   na.rm = TRUE),
    CAT_PR  = rowSums(across(all_of(c("FuPoNC_4", "PaNeCo_4", "PaNeCo_5", "PaNeCo_1", "PaNeCo_2", "PaPoCo_2", "PrNeCo_3", "PrNeCo_4", "PrNeCo_1"))),     na.rm = TRUE),
    CAT_PH  = rowSums(across(all_of(c("FuPoNC_1", "FuPoNC_2", "FuPoNC_3", "PrPoNC_2", "PrPoNC_1"))),                                                       na.rm = TRUE),
    CAT_PNe = rowSums(across(all_of(c("PaNeNC_2", "PaNeNC_4", "PaNeNC_1"))),                                                                               na.rm = TRUE),
    CAT_PNo = rowSums(across(all_of(c("PaPoNC_3", "PaPoNC_4", "PaPoNC_1", "PaPoNC_2", "PrNeNC_4", "PrNeNC_2", "PrPoNC_4", "PaNeNC_3"))),                  na.rm = TRUE),
    CAT_PE  = rowSums(across(all_of(c("PrPoCo_3", "PrPoCo_4", "PrPoCo_5", "PrPoCo_1"))),                                                                   na.rm = TRUE)
  )
```

## Behavioural Indices — Subject-Level Aggregation

Performance indices are computed at the subject level across
stimulation conditions, consistent with the approach described in
§2.5.7.

```
# .$MTT_RT      : average inverse RT (correct, non-outlier trials)
# .$MTT_ACC     : logit-transformed mean accuracy
# .$DD_k        : log-transformed mean hyperbolic discounting rate k
# .$PM_t_CHECK  : mean normalised clock-check timing
# .$PM_t_PRESS  : mean normalised button-press timing
# .$PM_err      : mean absolute deviation from 2-min target (Box-Cox transformed)
# .$PM_n_CHECK  : mean number of clock checks per cycle

data_beh <-
  list(
    data_MTT %>%
      group_by(id) %>%
      summarize(
        MTT_RT  = mean(1 / RT,      na.rm = TRUE),
        MTT_ACC = car::logit(mean(correct, na.rm = TRUE))
      ),
    data_DD_subj_level %>%
      group_by(id) %>%
      summarize(
        DD_k = log(mean(k, na.rm = TRUE))
      ),
    data_PM_press %>%
      group_by(id) %>%
      summarize(
        PM_t_CHECK = mean(TIME_CHECK, na.rm = TRUE),
        PM_t_PRESS = mean(TIME_PRESS, na.rm = TRUE),
        PM_err     = mean(err_abs_t,  na.rm = TRUE)
      ),
    data_PM_n_CHECK %>%
      group_by(id) %>%
      summarize(
        PM_n_CHECK = mean(nCHECK, na.rm = TRUE)
      )
  ) %>%
  reduce(full_join, by = "id")
```

## Final Dataset Assembly

All sources are joined at the subject level. The subject identifier
is dropped prior to any further analysis.

```
data_scores <-
  id_map %>%
  left_join(CAT_composite,  by = c("id" = "subject_id")) %>%
  left_join(data_beh,       by = c("id_hash" = "id")) %>%
  left_join(subj_data_STIM, by = c("id" = "subject_id")) %>%
  select(-id)
```

# CORRELATION ANALYSIS

Spearman correlation coefficients are computed pairwise across all
numeric indices. Significance (p-values) is estimated via
`corrplot::cor.mtest()` on listwise-complete cases.

```
temp <-
  id_map %>%
  left_join(CAT_composite,  by = c("id" = "subject_id")) %>%
  left_join(data_beh,       by = c("id_hash" = "id")) %>%
  left_join(subj_data_STIM, by = c("id" = "subject_id")) %>%
  select(-id)

temp_num   <- temp %>% select(where(is.numeric))
cor_full   <- cor(temp_num, method = "spearman", use = "pairwise.complete.obs")

temp_clean <- temp_num %>% na.omit()
p_vals     <- suppressWarnings(
  corrplot::cor.mtest(temp_clean, method = "spearman", conf.level = 0.95)
)$p
```

Spearman correlation matrix (3 decimal places):

```
kable(round(cor_full, 3), align = "c")
```

|  | CAT\_FA | CAT\_FP | CAT\_PF | CAT\_PR | CAT\_PH | CAT\_PNe | CAT\_PNo | CAT\_PE | MTT\_RT | MTT\_ACC | DD\_k | PM\_t\_CHECK | PM\_t\_PRESS | PM\_err | PM\_n\_CHECK | avgARpast | avgVALpast | avgARfut | avgVALfut | avgAR | avgVAL | diffAR | diffVAL |
| --- | --- | --- | --- | --- | --- | --- | --- | --- | --- | --- | --- | --- | --- | --- | --- | --- | --- | --- | --- | --- | --- | --- | --- |
| CAT\_FA | 1.000 | 0.255 | 0.232 | -0.481 | -0.378 | 0.519 | 0.506 | -0.092 | -0.082 | -0.164 | -0.136 | -0.026 | 0.064 | 0.099 | 0.083 | -0.143 | -0.247 | -0.054 | -0.153 | -0.105 | -0.249 | 0.052 | 0.029 |
| CAT\_FP | 0.255 | 1.000 | -0.445 | 0.130 | -0.362 | 0.091 | -0.267 | 0.187 | 0.066 | 0.035 | -0.062 | -0.053 | 0.098 | -0.071 | 0.384 | -0.024 | -0.179 | -0.214 | 0.126 | -0.060 | -0.027 | -0.365 | 0.169 |
| CAT\_PF | 0.232 | -0.445 | 1.000 | -0.349 | 0.099 | 0.080 | 0.539 | 0.043 | -0.303 | -0.123 | 0.077 | 0.353 | -0.031 | 0.168 | -0.112 | -0.151 | -0.029 | 0.061 | -0.313 | -0.094 | -0.203 | 0.274 | -0.259 |
| CAT\_PR | -0.481 | 0.130 | -0.349 | 1.000 | 0.418 | -0.412 | -0.356 | 0.425 | -0.062 | 0.321 | 0.193 | -0.248 | -0.158 | -0.471 | 0.360 | -0.003 | 0.263 | 0.137 | 0.250 | 0.046 | 0.347 | 0.044 | 0.048 |
| CAT\_PH | -0.378 | -0.362 | 0.099 | 0.418 | 1.000 | -0.219 | 0.207 | 0.342 | -0.107 | 0.015 | 0.260 | 0.033 | -0.020 | -0.031 | -0.101 | 0.224 | 0.344 | 0.418 | 0.389 | 0.305 | 0.455 | 0.250 | 0.164 |
| CAT\_PNe | 0.519 | 0.091 | 0.080 | -0.412 | -0.219 | 1.000 | 0.323 | -0.288 | -0.049 | -0.067 | -0.277 | 0.073 | 0.236 | 0.260 | -0.179 | -0.109 | -0.061 | -0.101 | -0.065 | -0.107 | -0.096 | 0.066 | 0.061 |
| CAT\_PNo | 0.506 | -0.267 | 0.539 | -0.356 | 0.207 | 0.323 | 1.000 | -0.020 | -0.213 | -0.176 | -0.033 | 0.292 | -0.001 | 0.081 | -0.220 | -0.151 | -0.002 | -0.033 | -0.235 | -0.125 | -0.120 | 0.123 | -0.180 |
| CAT\_PE | -0.092 | 0.187 | 0.043 | 0.425 | 0.342 | -0.288 | -0.020 | 1.000 | 0.000 | 0.162 | 0.086 | -0.025 | 0.029 | -0.404 | 0.325 | 0.168 | 0.316 | 0.225 | 0.459 | 0.204 | 0.523 | -0.078 | 0.116 |
| MTT\_RT | -0.082 | 0.066 | -0.303 | -0.062 | -0.107 | -0.049 | -0.213 | 0.000 | 1.000 | 0.301 | -0.099 | -0.263 | -0.222 | -0.165 | -0.393 | 0.070 | 0.289 | -0.092 | -0.059 | -0.008 | 0.136 | -0.209 | -0.206 |
| MTT\_ACC | -0.164 | 0.035 | -0.123 | 0.321 | 0.015 | -0.067 | -0.176 | 0.162 | 0.301 | 1.000 | -0.078 | -0.383 | 0.012 | -0.334 | 0.075 | 0.125 | 0.197 | 0.050 | -0.169 | 0.070 | 0.036 | -0.208 | -0.262 |
| DD\_k | -0.136 | -0.062 | 0.077 | 0.193 | 0.260 | -0.277 | -0.033 | 0.086 | -0.099 | -0.078 | 1.000 | -0.110 | -0.495 | -0.252 | 0.183 | -0.243 | 0.057 | -0.138 | 0.060 | -0.182 | 0.056 | 0.037 | -0.013 |
| PM\_t\_CHECK | -0.026 | -0.053 | 0.353 | -0.248 | 0.033 | 0.073 | 0.292 | -0.025 | -0.263 | -0.383 | -0.110 | 1.000 | 0.657 | 0.453 | -0.333 | -0.030 | -0.151 | 0.000 | -0.099 | 0.000 | -0.147 | 0.028 | -0.017 |
| PM\_t\_PRESS | 0.064 | 0.098 | -0.031 | -0.158 | -0.020 | 0.236 | -0.001 | 0.029 | -0.222 | 0.012 | -0.495 | 0.657 | 1.000 | 0.358 | -0.123 | 0.147 | -0.375 | 0.111 | 0.099 | 0.143 | -0.154 | -0.038 | 0.349 |
| PM\_err | 0.099 | -0.071 | 0.168 | -0.471 | -0.031 | 0.260 | 0.081 | -0.404 | -0.165 | -0.334 | -0.252 | 0.453 | 0.358 | 1.000 | -0.320 | 0.043 | -0.202 | 0.034 | 0.104 | 0.035 | -0.234 | -0.071 | 0.286 |
| PM\_n\_CHECK | 0.083 | 0.384 | -0.112 | 0.360 | -0.101 | -0.179 | -0.220 | 0.325 | -0.393 | 0.075 | 0.183 | -0.333 | -0.123 | -0.320 | 1.000 | -0.333 | 0.041 | -0.331 | -0.033 | -0.331 | 0.014 | -0.191 | -0.203 |
| avgARpast | -0.143 | -0.024 | -0.151 | -0.003 | 0.224 | -0.109 | -0.151 | 0.168 | 0.070 | 0.125 | -0.243 | -0.030 | 0.147 | 0.043 | -0.333 | 1.000 | 0.171 | 0.774 | 0.404 | 0.960 | 0.353 | -0.216 | 0.251 |
| avgVALpast | -0.247 | -0.179 | -0.029 | 0.263 | 0.344 | -0.061 | -0.002 | 0.316 | 0.289 | 0.197 | 0.057 | -0.151 | -0.375 | -0.202 | 0.041 | 0.171 | 1.000 | 0.194 | 0.241 | 0.168 | 0.770 | -0.049 | -0.393 |
| avgARfut | -0.054 | -0.214 | 0.061 | 0.137 | 0.418 | -0.101 | -0.033 | 0.225 | -0.092 | 0.050 | -0.138 | 0.000 | 0.111 | 0.034 | -0.331 | 0.774 | 0.194 | 1.000 | 0.464 | 0.905 | 0.399 | 0.326 | 0.330 |
| avgVALfut | -0.153 | 0.126 | -0.313 | 0.250 | 0.389 | -0.065 | -0.235 | 0.459 | -0.059 | -0.169 | 0.060 | -0.099 | 0.099 | 0.104 | -0.033 | 0.404 | 0.241 | 0.464 | 1.000 | 0.482 | 0.762 | 0.094 | 0.761 |
| avgAR | -0.105 | -0.060 | -0.094 | 0.046 | 0.305 | -0.107 | -0.125 | 0.204 | -0.008 | 0.070 | -0.182 | 0.000 | 0.143 | 0.035 | -0.331 | 0.960 | 0.168 | 0.905 | 0.482 | 1.000 | 0.408 | 0.001 | 0.329 |
| avgVAL | -0.249 | -0.027 | -0.203 | 0.347 | 0.455 | -0.096 | -0.120 | 0.523 | 0.136 | 0.036 | 0.056 | -0.147 | -0.154 | -0.234 | 0.014 | 0.353 | 0.770 | 0.399 | 0.762 | 0.408 | 1.000 | 0.060 | 0.222 |
| diffAR | 0.052 | -0.365 | 0.274 | 0.044 | 0.250 | 0.066 | 0.123 | -0.078 | -0.209 | -0.208 | 0.037 | 0.028 | -0.038 | -0.071 | -0.191 | -0.216 | -0.049 | 0.326 | 0.094 | 0.001 | 0.060 | 1.000 | 0.192 |
| diffVAL | 0.029 | 0.169 | -0.259 | 0.048 | 0.164 | 0.061 | -0.180 | 0.116 | -0.206 | -0.262 | -0.013 | -0.017 | 0.349 | 0.286 | -0.203 | 0.251 | -0.393 | 0.330 | 0.761 | 0.329 | 0.222 | 0.192 | 1.000 |

p-value matrix:

```
kable(round(p_vals, 3), align = "c")
```

|  | CAT\_FA | CAT\_FP | CAT\_PF | CAT\_PR | CAT\_PH | CAT\_PNe | CAT\_PNo | CAT\_PE | MTT\_RT | MTT\_ACC | DD\_k | PM\_t\_CHECK | PM\_t\_PRESS | PM\_err | PM\_n\_CHECK | avgARpast | avgVALpast | avgARfut | avgVALfut | avgAR | avgVAL | diffAR | diffVAL |
| --- | --- | --- | --- | --- | --- | --- | --- | --- | --- | --- | --- | --- | --- | --- | --- | --- | --- | --- | --- | --- | --- | --- | --- |
| CAT\_FA | 0.000 | 0.246 | 0.342 | 0.028 | 0.012 | 0.008 | 0.019 | 0.788 | 0.566 | 0.526 | 0.350 | 0.810 | 0.934 | 0.578 | 0.463 | 0.196 | 0.085 | 0.227 | 0.209 | 0.206 | 0.032 | 0.829 | 0.820 |
| CAT\_FP | 0.246 | 0.000 | 0.201 | 0.229 | 0.109 | 0.558 | 0.262 | 0.181 | 0.559 | 0.466 | 0.606 | 0.815 | 0.546 | 0.772 | 0.014 | 0.358 | 0.397 | 0.171 | 0.569 | 0.284 | 0.809 | 0.128 | 0.492 |
| CAT\_PF | 0.342 | 0.201 | 0.000 | 0.052 | 0.736 | 0.865 | 0.005 | 0.687 | 0.226 | 0.364 | 0.391 | 0.087 | 0.897 | 0.249 | 0.336 | 0.719 | 0.589 | 0.659 | 0.392 | 0.654 | 0.339 | 0.975 | 0.567 |
| CAT\_PR | 0.028 | 0.229 | 0.052 | 0.000 | 0.056 | 0.063 | 0.032 | 0.121 | 0.859 | 0.077 | 0.551 | 0.269 | 0.775 | 0.025 | 0.103 | 0.884 | 0.263 | 0.613 | 0.324 | 0.752 | 0.097 | 0.959 | 0.834 |
| CAT\_PH | 0.012 | 0.109 | 0.736 | 0.056 | 0.000 | 0.085 | 0.783 | 0.092 | 0.846 | 0.863 | 0.087 | 0.885 | 0.776 | 0.607 | 0.712 | 0.240 | 0.050 | 0.047 | 0.160 | 0.113 | 0.023 | 0.324 | 0.792 |
| CAT\_PNe | 0.008 | 0.558 | 0.865 | 0.063 | 0.085 | 0.000 | 0.154 | 0.084 | 0.457 | 0.744 | 0.354 | 0.821 | 0.674 | 0.366 | 0.728 | 0.225 | 0.459 | 0.129 | 0.249 | 0.168 | 0.175 | 0.962 | 0.821 |
| CAT\_PNo | 0.019 | 0.262 | 0.005 | 0.032 | 0.783 | 0.154 | 0.000 | 0.966 | 0.651 | 0.412 | 0.928 | 0.181 | 0.798 | 0.515 | 0.147 | 0.248 | 0.841 | 0.271 | 0.032 | 0.233 | 0.268 | 0.748 | 0.098 |
| CAT\_PE | 0.788 | 0.181 | 0.687 | 0.121 | 0.092 | 0.084 | 0.966 | 0.000 | 0.830 | 0.426 | 0.676 | 0.966 | 0.671 | 0.045 | 0.093 | 0.355 | 0.169 | 0.195 | 0.009 | 0.192 | 0.003 | 0.763 | 0.420 |
| MTT\_RT | 0.566 | 0.559 | 0.226 | 0.859 | 0.846 | 0.457 | 0.651 | 0.830 | 0.000 | 0.302 | 0.799 | 0.236 | 0.139 | 0.183 | 0.117 | 0.866 | 0.239 | 0.705 | 0.811 | 0.837 | 0.362 | 0.595 | 0.521 |
| MTT\_ACC | 0.526 | 0.466 | 0.364 | 0.077 | 0.863 | 0.744 | 0.412 | 0.426 | 0.302 | 0.000 | 0.450 | 0.066 | 0.820 | 0.033 | 0.447 | 0.414 | 0.426 | 0.729 | 0.829 | 0.726 | 0.481 | 0.262 | 0.518 |
| DD\_k | 0.350 | 0.606 | 0.391 | 0.551 | 0.087 | 0.354 | 0.928 | 0.676 | 0.799 | 0.450 | 0.000 | 0.585 | 0.023 | 0.716 | 0.722 | 0.114 | 0.461 | 0.474 | 0.800 | 0.247 | 0.620 | 0.475 | 0.948 |
| PM\_t\_CHECK | 0.810 | 0.815 | 0.087 | 0.269 | 0.885 | 0.821 | 0.181 | 0.966 | 0.236 | 0.066 | 0.585 | 0.000 | 0.001 | 0.037 | 0.111 | 0.894 | 0.489 | 1.000 | 0.652 | 1.000 | 0.501 | 0.901 | 0.941 |
| PM\_t\_PRESS | 0.934 | 0.546 | 0.897 | 0.775 | 0.776 | 0.674 | 0.798 | 0.671 | 0.139 | 0.820 | 0.023 | 0.001 | 0.000 | 0.387 | 0.923 | 0.747 | 0.090 | 0.849 | 0.816 | 0.774 | 0.389 | 0.930 | 0.243 |
| PM\_err | 0.578 | 0.772 | 0.249 | 0.025 | 0.607 | 0.366 | 0.515 | 0.045 | 0.183 | 0.033 | 0.716 | 0.037 | 0.387 | 0.000 | 0.337 | 0.771 | 0.516 | 0.696 | 0.776 | 0.733 | 0.206 | 0.795 | 0.405 |
| PM\_n\_CHECK | 0.463 | 0.014 | 0.336 | 0.103 | 0.712 | 0.728 | 0.147 | 0.093 | 0.117 | 0.447 | 0.722 | 0.111 | 0.923 | 0.337 | 0.000 | 0.189 | 0.898 | 0.183 | 0.767 | 0.204 | 0.814 | 0.281 | 0.649 |
| avgARpast | 0.196 | 0.358 | 0.719 | 0.884 | 0.240 | 0.225 | 0.248 | 0.355 | 0.866 | 0.414 | 0.114 | 0.894 | 0.747 | 0.771 | 0.189 | 0.000 | 0.186 | 0.000 | 0.169 | 0.000 | 0.097 | 0.747 | 0.774 |
| avgVALpast | 0.085 | 0.397 | 0.589 | 0.263 | 0.050 | 0.459 | 0.841 | 0.169 | 0.239 | 0.426 | 0.461 | 0.489 | 0.090 | 0.516 | 0.898 | 0.186 | 0.000 | 0.250 | 0.152 | 0.216 | 0.000 | 0.567 | 0.078 |
| avgARfut | 0.227 | 0.171 | 0.659 | 0.613 | 0.047 | 0.129 | 0.271 | 0.195 | 0.705 | 0.729 | 0.474 | 1.000 | 0.849 | 0.696 | 0.183 | 0.000 | 0.250 | 0.000 | 0.059 | 0.000 | 0.051 | 0.106 | 0.335 |
| avgVALfut | 0.209 | 0.569 | 0.392 | 0.324 | 0.160 | 0.249 | 0.032 | 0.009 | 0.811 | 0.829 | 0.800 | 0.652 | 0.816 | 0.776 | 0.767 | 0.169 | 0.152 | 0.059 | 0.000 | 0.076 | 0.000 | 0.404 | 0.000 |
| avgAR | 0.206 | 0.284 | 0.654 | 0.752 | 0.113 | 0.168 | 0.233 | 0.192 | 0.837 | 0.726 | 0.247 | 1.000 | 0.774 | 0.733 | 0.204 | 0.000 | 0.216 | 0.000 | 0.076 | 0.000 | 0.056 | 0.516 | 0.472 |
| avgVAL | 0.032 | 0.809 | 0.339 | 0.097 | 0.023 | 0.175 | 0.268 | 0.003 | 0.362 | 0.481 | 0.620 | 0.501 | 0.389 | 0.206 | 0.814 | 0.097 | 0.000 | 0.051 | 0.000 | 0.056 | 0.000 | 0.774 | 0.713 |
| diffAR | 0.829 | 0.128 | 0.975 | 0.959 | 0.324 | 0.962 | 0.748 | 0.763 | 0.595 | 0.262 | 0.475 | 0.901 | 0.930 | 0.795 | 0.281 | 0.747 | 0.567 | 0.106 | 0.404 | 0.516 | 0.774 | 0.000 | 0.120 |
| diffVAL | 0.820 | 0.492 | 0.567 | 0.834 | 0.792 | 0.821 | 0.098 | 0.420 | 0.521 | 0.518 | 0.948 | 0.941 | 0.243 | 0.405 | 0.649 | 0.774 | 0.078 | 0.335 | 0.000 | 0.472 | 0.713 | 0.120 | 0.000 |

# VISUALIZATION

```
palette <- colorRampPalette(c("#0072B2", "#FFFFFF", "#D55E00"))(200)

corrplot(cor_full,
         method      = "square",
         col         = palette,
         type        = "full",
         tl.col      = "black",
         tl.srt      = 45,
         diag        = FALSE,
         cl.pos      = "r",
         addgrid.col = "white",
         sig.level   = 0.05,
         insig       = "blank")
```
