# Supplementary material for: Beta-Band Stimulation of Dorsolateral Prefrontal Cortex Through tACS Alters Mental Time Travel and Prospective Memory, but Not Delay Discounting
Source: Brain Sci. 2026 Aug 22;16(9):899. doi: 10.3390/brainsci16090899 (PMC13604682; doi:10.3390/brainsci16090899)
Supplement: Supplementary file 1 [file brainsci-16-00899-s001.zip › S8 FDR Correction (code markdown).html]

tACS\_future\_FDR\_Correction


# tACS\_future\_FDR\_Correction

#### Authors are masked for peer review purposes

# FDR CORRECTION ACROSS ALL EMM CONTRASTS

```
contrasts_list <- list(
  `MTT ACC — stim x ref_cat` = pairs(emmeans(m_MTT_ACC_int, ~ stim | ref_cat)),
  `DD — stimulation` = pairs(emmeans(m_DD_K_no_int, ~ stimulation)),
  `PM check freq — quartile` = pairs(emmeans(m_PM_CC_n_null, ~ QUARTILE)),
  `PM check timing — quartile` = pairs(emmeans(m_PM_CC_t_null, ~ QUARTILE)),
  `PM dichot ACC — stimulation` = pairs(emmeans(m_PM_BP_c_no_int, ~ stimulation)),
  `PM cont ACC — stimulation` = pairs(emmeans(m_PM_BP_t_no_int, ~ stimulation))
)

all_contrasts <- bind_rows(lapply(names(contrasts_list), function(nm) {
  as.data.frame(contrasts_list[[nm]]) %>% mutate(Analysis = nm, .before = 1)
}))

all_contrasts <- all_contrasts %>%
  mutate(p_fdr = p.adjust(p.value, method = "fdr"))

kable(all_contrasts, digits = 3, caption = "FDR-corrected pairwise EMM contrasts across all models")
```

FDR-corrected pairwise EMM contrasts across all
models

| Analysis | contrast | ref\_cat | estimate | SE | df | z.ratio | p.value | t.ratio | p\_fdr |
| --- | --- | --- | --- | --- | --- | --- | --- | --- | --- |
| MTT ACC — stim x ref\_cat | beta - sham | 2014 | 0.727 | 0.212 | Inf | 3.438 | 0.001 | NA | 0.001 |
| MTT ACC — stim x ref\_cat | beta - sham | 2024 | 0.916 | 0.206 | Inf | 4.446 | 0.000 | NA | 0.000 |
| MTT ACC — stim x ref\_cat | beta - sham | 2034 | 0.201 | 0.174 | Inf | 1.155 | 0.248 | NA | 0.298 |
| DD — stimulation | beta - sham | NA | 0.345 | 0.172 | 163.000 | NA | 0.046 | 2.007 | 0.060 |
| PM check freq — quartile | 1st - 2nd | NA | -0.065 | 0.163 | Inf | -0.400 | 0.978 | NA | 0.978 |
| PM check freq — quartile | 1st - 3rd | NA | -0.274 | 0.157 | Inf | -1.751 | 0.297 | NA | 0.334 |
| PM check freq — quartile | 1st - 4th | NA | -0.818 | 0.147 | Inf | -5.575 | 0.000 | NA | 0.000 |
| PM check freq — quartile | 2nd - 3rd | NA | -0.209 | 0.128 | Inf | -1.633 | 0.360 | NA | 0.381 |
| PM check freq — quartile | 2nd - 4th | NA | -0.752 | 0.115 | Inf | -6.521 | 0.000 | NA | 0.000 |
| PM check freq — quartile | 3rd - 4th | NA | -0.543 | 0.105 | Inf | -5.198 | 0.000 | NA | 0.000 |
| PM check timing — quartile | 1st - 2nd | NA | -0.444 | 0.025 | 556.829 | NA | 0.000 | -17.474 | 0.000 |
| PM check timing — quartile | 1st - 3rd | NA | -0.923 | 0.025 | 548.082 | NA | 0.000 | -37.589 | 0.000 |
| PM check timing — quartile | 1st - 4th | NA | -1.498 | 0.023 | 524.202 | NA | 0.000 | -64.741 | 0.000 |
| PM check timing — quartile | 2nd - 3rd | NA | -0.480 | 0.020 | 551.950 | NA | 0.000 | -24.141 | 0.000 |
| PM check timing — quartile | 2nd - 4th | NA | -1.055 | 0.018 | 553.421 | NA | 0.000 | -58.926 | 0.000 |
| PM check timing — quartile | 3rd - 4th | NA | -0.575 | 0.016 | 546.577 | NA | 0.000 | -35.339 | 0.000 |
| PM dichot ACC — stimulation | beta - sham | NA | 2.136 | 0.757 | Inf | 2.821 | 0.005 | NA | 0.007 |
| PM cont ACC — stimulation | beta - sham | NA | -0.067 | 0.027 | 118.504 | NA | 0.014 | -2.502 | 0.019 |
