# Supplementary material for: Beta-Band Stimulation of Dorsolateral Prefrontal Cortex Through tACS Alters Mental Time Travel and Prospective Memory, but Not Delay Discounting
Source: Brain Sci. 2026 Aug 22;16(9):899. doi: 10.3390/brainsci16090899 (PMC13604682; doi:10.3390/brainsci16090899)
Supplement: Supplementary file 1 [file brainsci-16-00899-s001.zip › S9 Supplementary Figures (boxplots).pdf]

Supplementary Figure S1

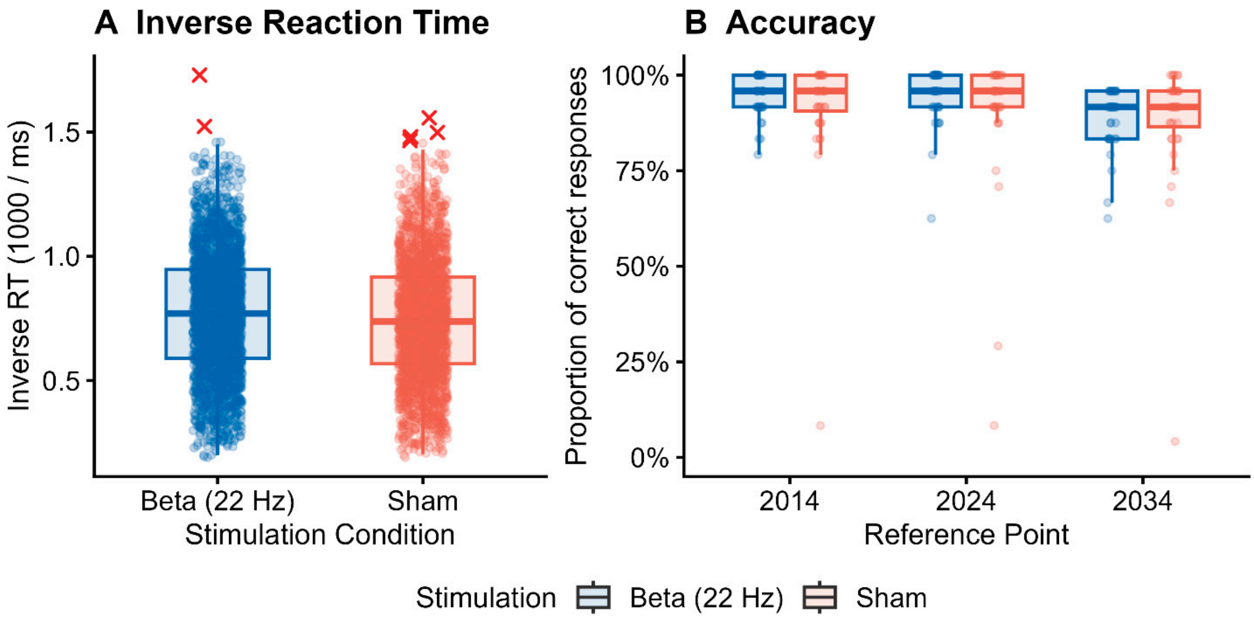

Supplementary Figure S1. *Mental Time Travel task: distribution of observations by stimulation condition.* Panel A) Boxplots of inverse reaction times for each stimulation condition. Individual trial-level observations are overlaid and horizontally jittered. Red crosses denote trials excluded as outliers before analysis, as described in Section 2.5.2. Panel B) Boxplots of participant-level proportions of correct responses by stimulation condition and reference point (2014, 2024, and 2034). Individual participant-level observations are overlaid and horizontally jittered.

Supplementary Figure S2

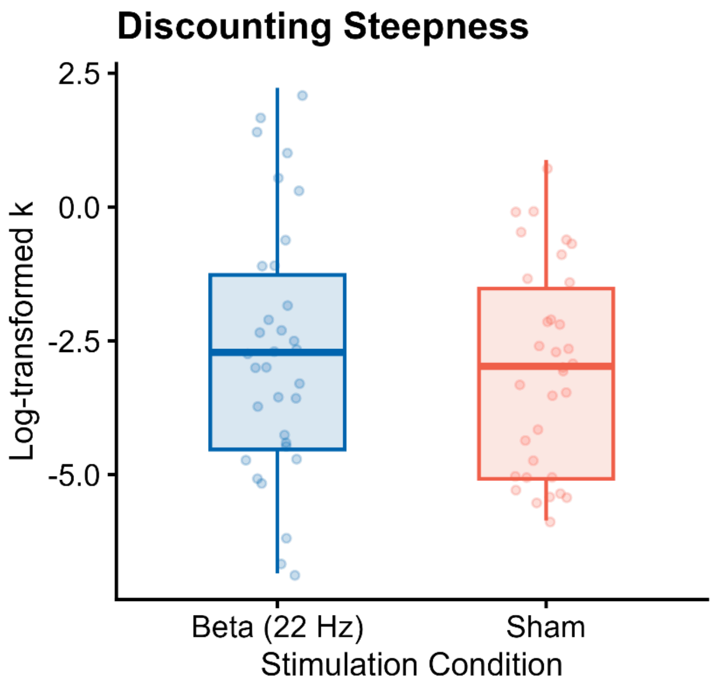

Supplementary Figure S2. *Delay Discounting task: distribution of individual discounting parameters by stimulation condition.* Boxplots show participant-level *k* parameter estimates, averaged across maximum-reward conditions and log-transformed, for each stimulation condition. Individual participant-level observations are overlaid and horizontally jittered.

Supplementary Figure S3

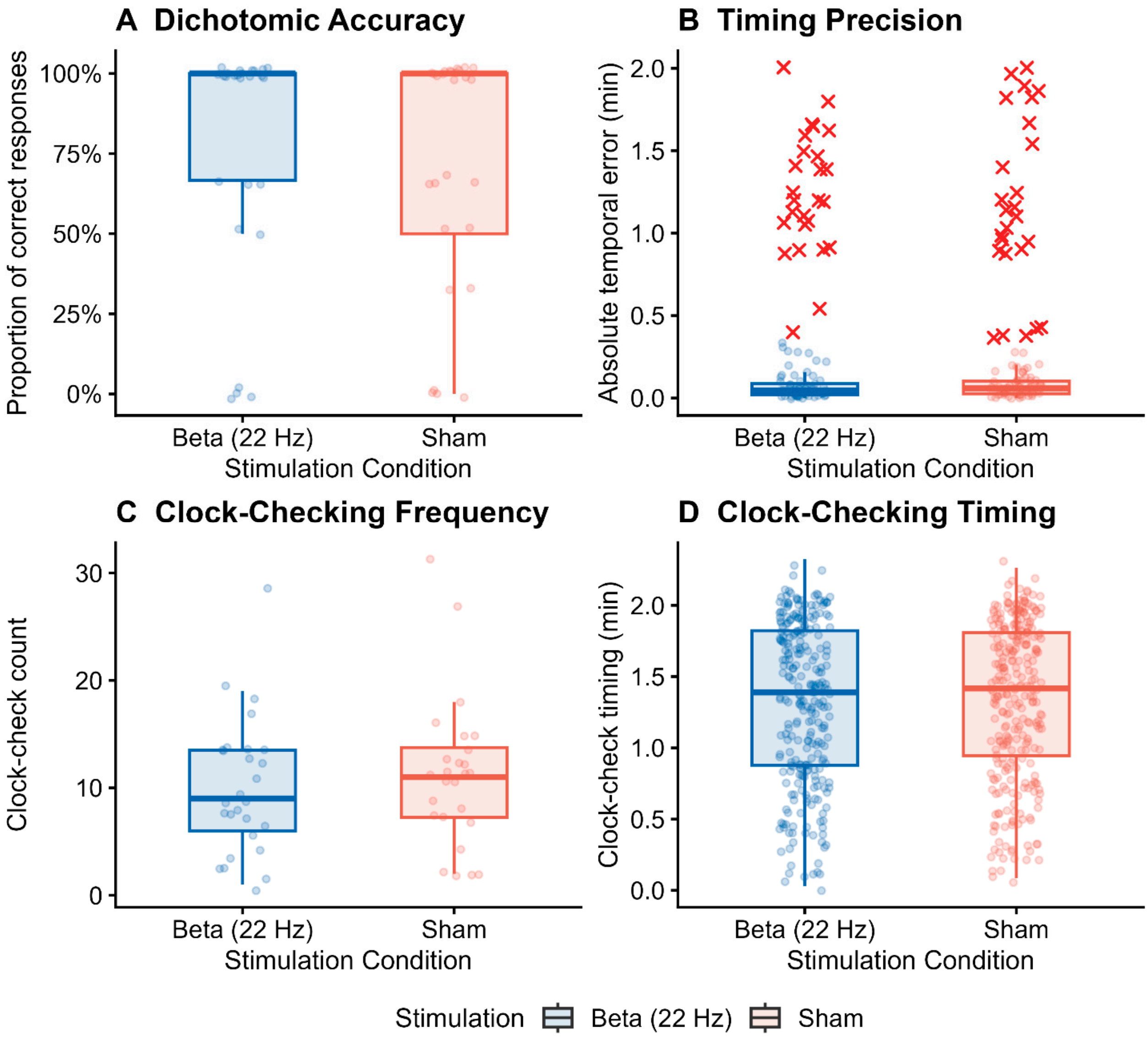

Supplementary Figure S3. *Prospective Memory task: distribution of observations by stimulation condition.* Panel A) Boxplots of participant-level proportions of correct responses in each stimulation condition. Individual participant-level observations are overlaid and horizontally jittered. Panel B) Boxplots of trial-level absolute temporal errors in each stimulation condition. Individual observations are overlaid and horizontally jittered; red crosses denote trials excluded as outliers before analysis, as described in Section 2.5.5. Panel C) Boxplots of participant-level clock-check counts in each stimulation condition. Individual participant-level observations are overlaid and horizontally jittered. Panel D) Boxplots of clock-check timing observations in each stimulation condition. Individual observations are overlaid and horizontally jittered.

Supplementary Figure S4

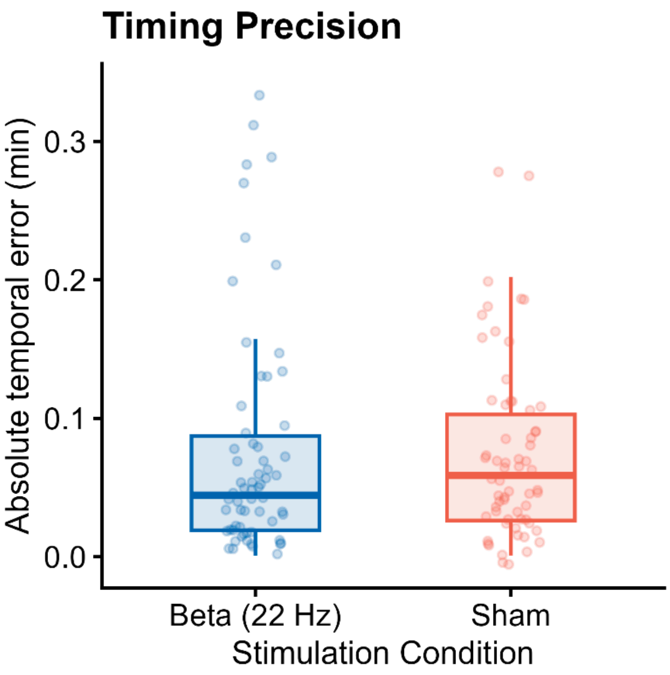

Supplementary Figure S4. *Prospective Memory task: timing precision after outlier exclusion.* Boxplots show trial-level absolute temporal errors in each stimulation condition after removal of outlying observations, as described in Section 2.5.5. Individual observations are overlaid and horizontally jittered. This single-panel version is provided to facilitate visualization of the distribution after outlier exclusion; the corresponding plot including excluded outliers is shown in Supplementary Figure 3B.
